# Bacterial communities found in placental tissues are associated with severe chorioamnionitis and adverse birth outcomes

**DOI:** 10.1371/journal.pone.0180167

**Published:** 2017-07-12

**Authors:** Ronan M. Doyle, Kathryn Harris, Steve Kamiza, Ulla Harjunmaa, Ulla Ashorn, Minyanga Nkhoma, Kathryn G. Dewey, Kenneth Maleta, Per Ashorn, Nigel Klein

**Affiliations:** 1 UCL Great Ormond Street Institute of Child Health, University College London, London, United Kingdom; 2 Department of Microbiology, Virology and Infection Control, Great Ormond Street Hospital NHS Foundation Trust, London, United Kingdom; 3 Department of Pathology, University of Malawi College of Medicine, Blantyre, Malawi; 4 Center for Child Health Research, University of Tampere Faculty of Medicine and Life Sciences, and Tampere University Hospital, Tampere, Finland; 5 Department for International Health, University of Tampere School of Medicine, Tampere, Finland; 6 Department of Nutrition, University of California Davis, Davis, California, United States of America; 7 Department of Community Health, University of Malawi College of Medicine, Blantyre, Malawi; 8 Department of Paediatrics, University of Tampere School of Medicine, Tampere, Finland; 9 Department of Paediatrics, Tampere University Hospital, Tampere, Finland; BC Children's Hospital, CANADA

## Abstract

Preterm birth is a major cause of neonatal mortality and morbidity worldwide. Bacterial infection and the subsequent inflammatory response are recognised as an important cause of preterm birth. It is hypothesised that these organisms ascend the cervical canal, colonise placental tissues, cause chorioamnionitis and in severe cases infect amniotic fluid and the foetus. However, the presence of bacteria within the intrauterine cavity does not always precede chorioamnionitis or preterm birth. Whereas previous studies observing the types of bacteria present have been limited in size and the specificity of a few predetermined organisms, in this study we characterised bacteria found in placental tissues from a cohort of 1391 women in rural Malawi using 16S ribosomal RNA gene sequencing. We found that specific bacteria found concurrently on placental tissues associate with chorioamnionitis and delivery of a smaller newborn. Severe chorioamnionitis was associated with a distinct difference in community members, a higher bacterial load and lower species richness. Furthermore, *Sneathia sanguinengens* and *Peptostreptococcus anaerobius* found in both matched participant vaginal and placental samples were associated with a lower newborn length-for-age Z-score. This is the largest study to date to examine the placental microbiome and its impact of birth outcomes. Our results provide data on the role of the vaginal microbiome as a source of placental infection as well as the possibility of therapeutic interventions against targeted organisms during pregnancy.

## Introduction

Preterm birth is the largest cause of neonatal deaths in the world [1]. Death rates are particularly high in developing countries. Compared with other regions, sub-Saharan Africa has had consistently higher incidence of preterm deliveries [2] with recent estimates of between 10.0% [3] and 16.3% [4] of all births in Malawi. Even in the developed world, incidences of preterm birth are increasing and it remains the major cause of perinatal mortality [2]. Of the children who survive extreme prematurity (less than 26 weeks), 19% will develop severe disability [5].

While the aetiology of spontaneous preterm labour remains elusive, a role for bacterial infection and colonisation of foetal tissue and the associated maternal inflammatory immune response is now recognized as the probable trigger in some cases. Spontaneous preterm birth (SPTB) is distinguished by a higher frequency of bacterial colonisation and by the nature and diversity of bacterial species [6]. There is increasing evidence to suggest that it is the type of bacteria present in preterm deliveries, rather than simply the presence of bacteria, that differs from term deliveries [7–9]. While there are hypotheses that bacteria can infect foetal tissues through haematogenous spread, in the majority of cases the common route is thought to be ascending infection from the vagina into the cervical canal [10]. It appears that it is these bacteria that lead to inflammation of the chorioamniotic tissues (chorioamnionitis), well recognised as being highly associated with earlier delivery [10,11].

Most studies to date have been conducted in Europe and North America. In this study we aimed to describe the microbiota found in placental tissue and fetal membranes (chorion and amnion) from a cohort of women in rural southern Malawi. We examined the microbial community structure and explored if this differed in placental tissue exhibiting chorioamnionitis. We also determined the relationship of the placental microbiome with birth weight, newborn length, duration of pregnancy and head circumference. We also collected oral and vaginal swabs from the same individuals to study the potential source of organisms found in the microbiomes of the placenta and fetal membranes.

## Materials and methods

### Study design and enrolment

This cross-sectional study was part of a larger clinical trial assessing whether providing Lipid-based Nutrient Supplements (LNS) to mothers during pregnancy and for 6 months postpartum, and to the infant from 6 to 18 months of age, improves child growth to a greater extent than prenatal iron and folic acid (IFA) or multiple micronutrient (MMN) tablets [3]. Participants were enrolled prospectively into the main trial before 20 weeks gestation and followed throughout pregnancy, childbirth and beyond. The main intervention outcomes in the trial were birth weight and newborn length. After delivery we studied the association between placental and fetal membrane microbiota, inflammation and birth outcomes. All women enrolled as part of the main trial who delivered were included in these analyses and all analyses were adjusted for intervention group. Further methodological details around study design, setting, logistics and data management are provided in the full trial results [12–14].

### Study setting

Recruitment took place in 4 centres in Mangochi District, Southern Malawi: Mangochi district hospital, Malindi hospital, Lungwena health centre and Namwera health centre.

### Collection of birth outcome and baseline data

At enrolment, participants’ weight, height and haemoglobin concentration were measured and obstetric history was recorded. Duration of pregnancy was measured using ultrasound. All participants were tested for malarial infection and HIV (unless they were already known to be HIV positive or opted out). At the first home visit to participants 1–2 weeks post-enrolment, information was gathered on demographic, social and economic background. Birth weight was measured as soon as possible after delivery while newborn length and head circumference were measured at the infant’s first clinic visit at 1–2 weeks old.

### Sample collection

After delivery the placenta was transferred to a sterile container in the hospital, health centre or home (wherever delivery took place), where it was kept in a covered container at temperatures ranging between 20°C-40°C. Tissue sampling of the placenta occurred immediately after delivery, unless delivery occurred overnight (in which case the placenta was sampled the following morning), or at home (in which case the container had to be transported to the nearest study clinic first). Two 5 cm x 1 cm pieces of the fetal (chorionic and amniotic) membrane were taken from the edge of the rupture site and two 0.5 cm x 0.5 cm pieces of placental tissue at full thickness were taken from near the umbilical cord insertion. One fetal membrane and one placenta sample were placed in separate cryovials. If the sample collection took place in Mangochi district hospital, the cryovials were placed at -80°C. If sample collection took place at an outlying health centre or Malindi hospital, the samples were stored at -20°C for a maximum of two days before being transferred to -80°C storage at Mangochi district hospital. Vaginal mucus samples were taken at a postnatal visit (approx. 1 week after delivery). Swabs were inserted approximately 7 cm deep into the participant’s vagina, without a visual control, and rotated before withdrawal. After the sample collection, swabs were stored at -80°C. Dental swabs were collected one week after delivery or as soon as possible by rubbing the gingival margin of each tooth with the swab, avoiding skin contact. Swabs were placed into a -20°C freezer and as soon as possible moved the swab into a -80°C freezer. Timing of each stage of the collection procedure was recorded.

### Histologic examination

The remaining placenta and fetal membrane samples not in cryovials were placed in 10% neutral buffered formalin fixative, processed and embedded in paraffin wax. These were sectioned at 3–5 micron thick and stained with haematoxylin and eosin before being read. All placenta and fetal membrane tissue samples were scored for histologic chorioamnionitis by SK who was blinded to all clinical information and samples were only identifiable by study number. Chorioamnionitis was defined as the presence of inflammation (specifically, the presence of neutrophils) in either the chorion or the amnion (fetal membranes) tissues that surround the fetus. Chorioamnionitis can differ greatly in scale, so we defined chorioamnionitis as ≥5 neutrophil granulocytes on average per 10 high power fields present in either the chorionic plate or the amniotic membrane, and severe chorioamnionitis was defined as >25 neutrophil granulocytes, which was adapted from a previous study [15]. We included premature rupture of membranes as a cause of preterm birth and excluded other causes such as preterm labour (no known cause), antepartum haemorrhage, hypertensive disorders and an incompetent cervix.

### DNA extraction

DNA extraction was carried out for all sample types using the QIAmp DNA mini kit (Qiagen, Germany) as per the manufacturer's protocol with an additional cell disruption step after lysis with Proteinase K. In the additional step, 0.1mm glass beads (Lysing Matrix B, MP Biomedicals) were added to each sample and the 2ml tubes were shaken on a cell disrupter (Vortex Genie 2, Scientific Industries) for 10 minutes at the highest speed. For every 10 extractions, a negative extraction control was included (200μl buffer AE).

### 16S rDNA broad-range qPCR

All DNA samples purified from placenta and fetal membrane samples were screened for bacteria using a quantitative PCR (qPCR) SYBR green fluorescent dye assay. The following primer pair targeted the V5-7 regions of the 16S rRNA gene, 785F: 5′-GGATTAGATACCCBRGTAGTC-3′, 1175R: 5′-ACGTCRTCCCCDCCTTCCTC-3′ [7]. Each PCR reaction was carried out with the following, 1x Power SYBR Green master mix (Life technologies), 0.4*μ*M of forward and reverse primers, 1μl of template DNA and molecular grade water (Bioline) to give a final volume of 25μl. Amplification took place in an ABI 7300 Real-Time system (Life technologies) under the following conditions: 95°C×10 min, 40 cycles of 95°C×15 sec and 60°C×1 min. Each PCR run included three negative PCR controls (1μl buffer AE from QIAmp DNA mini kit), and a serial dilution of a known amount of positive control from a pure *Escherichia coli* culture for quantification used to calculate the bacterial load for each sample. 16S rRNA copy number for each *E*. *coli* dilution was calculated from the genome molecular weight and DNA concentration as measured by a Qubit 2.0 (Life technologies) and corrected for the seven 16S rRNA copies within the *E*. *coli* genome. Samples were defined as positive for bacterial DNA if their Ct value was lower than the lower limit of detection for this assay. This was determined to be 28 ± 3 cycles or equivalent to 40 CFU/μl depending on variation between runs and was determined using calibration curves generated from serial dilution of a known amount of positive control from a pure *Escherichia coli* culture.

### 16S rDNA amplicon high-throughput sequencing

Placental and fetal membrane samples positive for bacterial DNA by qPCR were selected for sequencing. Library preparation was carried out using dual-indexed forward and reverse primers, with barcodes taken from a previous study [16]. The full list and combinations of adapted primers used can be found in S1 Table. Each library preparation PCR was carried out with 1X Molzym PCR Buffer, 200 μM dNTPs (Bioline), 0.4 μM forward and reverse primer, 25 mM Moltaq, 5μl template DNA and molecular grade water (Bioline) to give a final reaction volume of 25μl. The reaction was amplified under the following conditions depending on the sample type. Placenta and fetal membrane samples were amplified under the following conditions: 94°C×3 min, 32 cycles of 94°C×30 sec, 60°C×40 sec and 72°C×90 sec, with a final extension cycle of 72°C×10 min. Vaginal and oral swab samples were amplified under the following conditions: 94°C×3 min, 30 cycles of 94°C×30 sec, 60°C×40 sec and 72°C×90 sec, with a final extension cycle of 72°C×10 min. The resulting amplicon was cleaned and pooled using SequalPrep normalization plate kits (Invitrogen) and AMPure XP beads (Beckman Coulter) both as per manufacturer’s protocol. Each plate was pooled into an equimolar final library after quantification using a Qubit 2.0 (Life technologies). Library was loaded onto a MiSeq (Illumina) as per manufacturer’s protocol for 500 cycle V2 kits with the addition of custom sequencing primers for read 1 (TACCGGGACTTAGGATTAGATACCCBRGTAGTC), read 2 (AACACGTTTTAACGTCRTCCCCDCCTTCCTC) and index 1 (GAGGAAGGHGGGGAYGACGTTAAAACGTGTT).

### Bioinformatics and statistical analysis

Paired-end sequenced reads from each MiSeq run were merged using FLASH [17] and demultiplexed, pooled and assigned OTUs (Operational Taxonomic Units) using QIIME v1.8.0 [18] by clustering sequences at 97% similarity against a small custom database of full length 16S rDNA sequences. Any sequences that failed to match at 97% were assigned against the full Greengenes database. Mock communities of known quantities of bacterial DNA from a mix of species were sequenced alongside samples and were used to filter the dataset for error. Negative sequencing and extraction controls were also sequenced and contaminating taxonomy were filtered from the dataset. After filtering steps, any samples with less than 1000 reads were removed. Alpha diversity (number of unique OTUs) and beta diversity (intra-individual unweighted UniFrac distances) were both calculated as implemented in QIIME after random subsampling without replacement to 1000 reads per sample. Diversity of microbes within an individual’s body site was summarised by taking the median value of their intra-individual UniFrac distances. This value is calculated through a pairwise comparison between the bacterial communities of all samples, with a high value representing a variable community compared to other individuals and a lower value representing a stable community [19]. The bacterial load of particular phyla, families or species was calculated by multiplying the overall bacteria load generated by qPCR by the proportion of their entire bacterial community that was occupied by that particular phylum. Where bacterial loads of different individual species were calculated, the 16S rRNA copy number was adjusted for those species according to how many 16S rRNA genes are known to be found in the genome. Birth weight as measured was used if recorded within 48 h of delivery; if not, birth weight was back calculated from weight measured at 6 or 13 days. If the weight was first measured within 2 to 5 days after delivery, when infants usually lose weight, birth weight was estimated by applying an age-dependent multiplicative factor to the measured weight [20]. Mean proxy for socioeconomic status was calculated as previously stated [21]. We defined preterm birth as delivery occurring before 37 full week’s gestation and low birth weight as <2500g. Length-for-age and head circumference-for-age z scores (LAZ and HCZ) were calculated using the WHO Child Growth Standards [22] and defined stunting as < -2.0 LAZ and small head circumference as < -2.0 HCZ. Statistical analysis was carried out with Stata v13 and R v3.1.0. For bivariate analyses we used either independent t-tests or chi-squared tests of independence as appropriate. Adjusted linear regression models were used to relate bacterial load, alpha and beta diversity to chorioamnionitis and birth outcomes. All regression models used to examine associations between OTUs and birth outcomes were adjusted for the possible effects of the intervention, maternal BMI at enrolment, maternal age, proxy for socioeconomic status, number of previous pregnancies, anaemia at enrolment, site of enrolment, mode of delivery and time between delivery and placenta sampling. Covariates entered into adjusted models were entered into the model in a one step, forced entry method. For comparisons between abundances of individual bacterial species and birth outcomes, the q-value was calculated using the Benjamini-Hochberg correction to control for the false discovery rate.

### Ethical approval and consent

Written consent was obtained from the mother at enrolment by either signature or thumbprint if not literate. If a thumbprint was obtained an impartial witness also attended and signed. The institutional review board approved this consent procedure. Ethical approval was obtained from College of Medicine Research and Ethics Committee (COMREC), Malawi (Protocol number: P.08/10/972). The trial was registered at clinicaltrials.gov as NCT01239693.

## Results

### Sample collection

**Fig 1:**
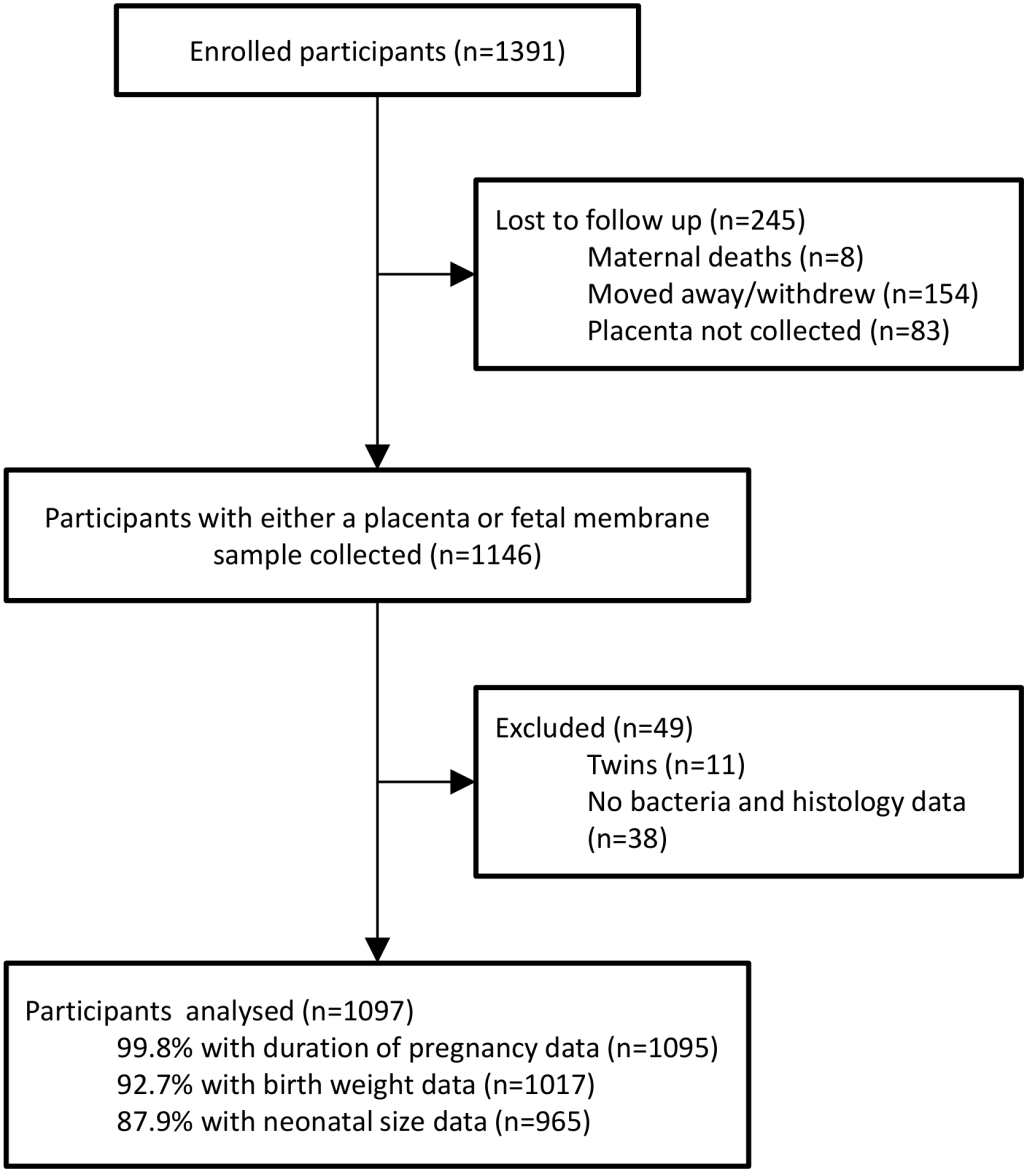
Study participant flow diagram.

A total of 1391 participants were recruited into the iLiNS-DYAD-M trial and enrolment began in February 2011 with the last delivery taking place in February 2013. 1097 (78.9%) participants had at least one placental or fetal membrane tissue analysed for bacteria and histology. Fig 1 shows a flow diagram that documents reasons for the loss to follow-up of the 21.1% of participants who were not included in this microbiome study. Of these, 49 were excluded because they had twins, a placental sample wasn’t collected or no histological result was obtained. Included participants had a lower BMI (22.1 vs 22.6 kg/m2, p = 0.005), were older (25 vs 24 years, p = 0.025), had completed less education (3.9 vs 4.7 years, p<0.001), had a lower score for socioeconomic status (-0.07 vs 0.36, p<0.001) and were less likely to be primiparous (20.4% vs 28.1%, p = 0.006) than those that were excluded (Table 1).

**Table 1:** Baseline characteristics of included and excluded participants (n = 1391).

Characteristic	Included (n = 1097)	Excluded (n = 294)	*P* value
Mean (SD) BMI, kg/m^2^	22.1 (2.8)	22.6 (3.0)	0.005
Mean (SD) maternal age, years	25.1 (6.1)	24.2 (6.3)	0.025
Mean (SD) maternal education, completed years at school	3.9 (3.4)	4.7 (3.8)	<0.001
Mean (SD) proxy for socioeconomic status	-0.07 (0.9)	0.36 (1.2)	<0.001
Proportion of aenemic women (Hb < 110 g/l)	19.9%	24.2%	0.118
Proportion of primiparous women	20.4%	28.1%	0.006
Proportion of women with a low BMI (< 18.5 kg/m^2^)	5.6%	4.7%	0.656
Proportion of women with a positive HIV test	13.3%	15.0%	0.469
Number (%) of women with a positive malaria test (RDT)	23.4%	22.7%	0.874

### Detection of bacterial DNA

Bacterial DNA was detected in 738 (68·1%) of fetal membranes and in 476 (46·8%) of placental samples. Of those participants who had detectable bacteria the mean (SD) bacterial load was 5.22 (0.84) Log^10^ 16S rDNA copies/μl in the fetal membranes and 4.80 (0.66) Log^10^ 16S rDNA/μl copies in the placenta. The median sequencing depths for placental tissue and fetal membranes were 11,803 and 21,040 reads per sample respectively (S2 Table).

### Impact of delay in freezing samples

**Fig 2:**
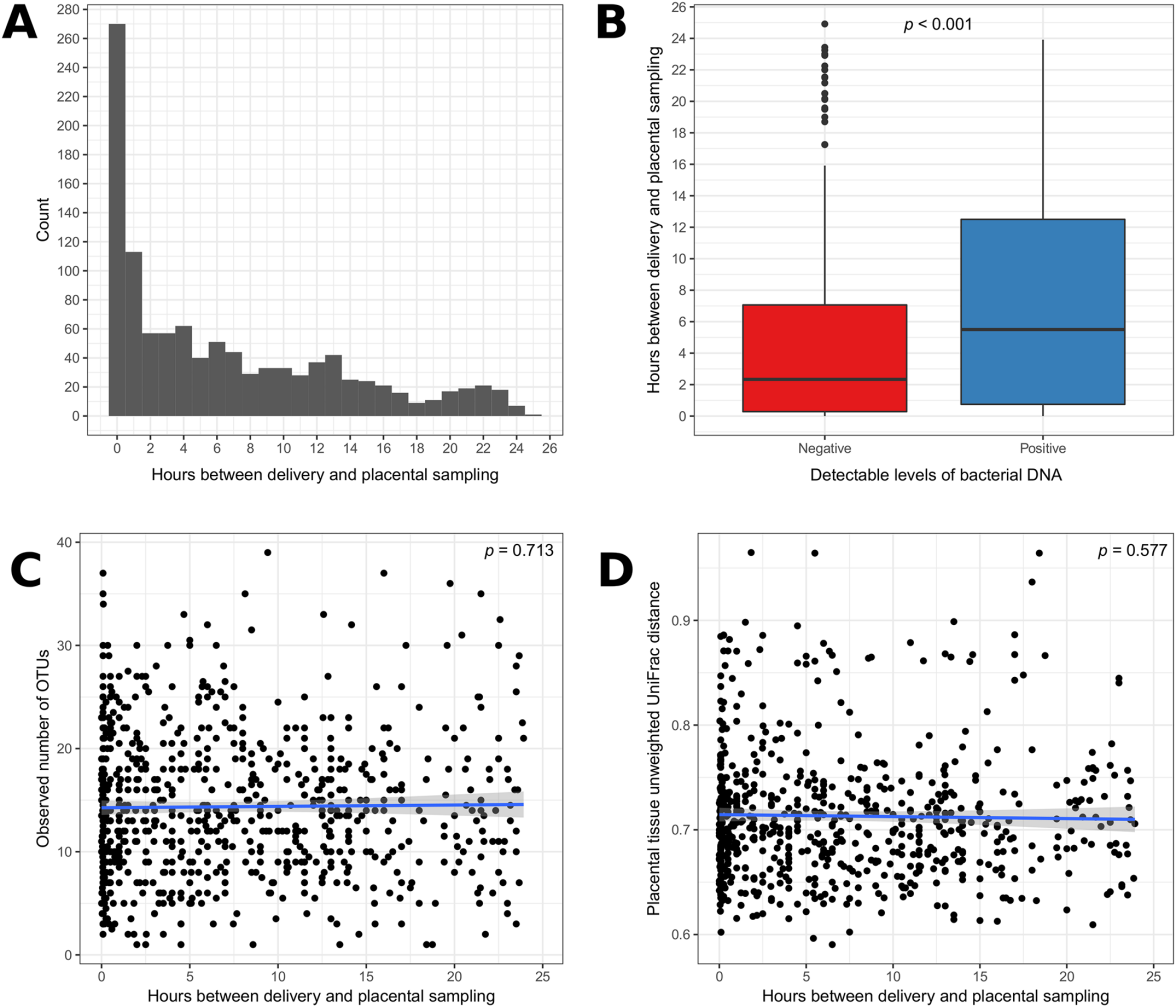
The effect of sample collection on detection of bacterial DNA. (A) Histogram of the number of hours between delivery and the placenta being sampled. (B) Box-and-whisker plot showing the association between the number of hours after delivery sampling took place and whether the placental tissue had detectable levels of bacterial DNA. Correlation between the number of hours after delivery the placenta was sampled (C), and the observed number of OTUs and median inter-individual unweighted UniFrac distance (D).

As described in the methods, the low-income setting of the population recruited to this study were largely rural, subsisting on farming and fishing, and as such many did not have easy access to healthcare facilities. As anticipated, this influenced the time between delivery, collection of placental samples and time to freezing. These delays could influence both the ability to detect bacterial DNA, as well as the bacterial content and relative abundance. The duration of storage at room temperature was recorded and we analysed the potential for cofounding associations. We found that while the majority of samples were taken within the first hour of delivery, the range of timings extended until more than 24 hours (Fig 2A). This delay in sampling influenced the chances of detecting 16S rRNA amplicon. Analysis of samples positive for bacterial DNA were collected at a mean difference of 3.2 hours later than those that had undetectable levels (p<0.001, Fig 2B). This enhanced detection of bacterial DNA may be because of growth of bacteria within/on tissues at birth, contaminating bacteria at the time of delivery that then had time to grow, or at the time of subsequent sample collection and processing. We investigated to see if we could identify which of these scenarios was most likely to be operating in this study by comparing the types of bacteria found on both placenta and fetal membrane tissues in relation to the delay in processing. We found no statistically significant difference in either in species richness or diversity in relation to the time after delivery that samples were taken (Fig 2C and 2D). We also examined the correlation between the time interval and each individual OTU identified in both placenta and fetal membranes. There was no impact of time to sampling on the OTUs found in placental tissue. There was a positive correlation between the relative abundance of two OTUs found in fetal membranes and the length of time between delivery and processing, after adjusting for multiple comparisons (S3 and S4 Tables). These two OTUs were of low abundance and therefore were not included in analyses of subsequent associations with chorioamnionitis or birth outcomes. We found no significant associations between home delivery and bacterial diversity and no associations between the time after delivery the placenta was sampled and any of the primary outcome measures (S1 Fig). However, as these data do not rule out possible associations between these variables and the outcome measures, we entered time after the delivery the placenta was sampled as a covariate in all subsequent regression analyses.

### The microbiome found in the placental tissues at delivery is distinct

**Fig 3:**
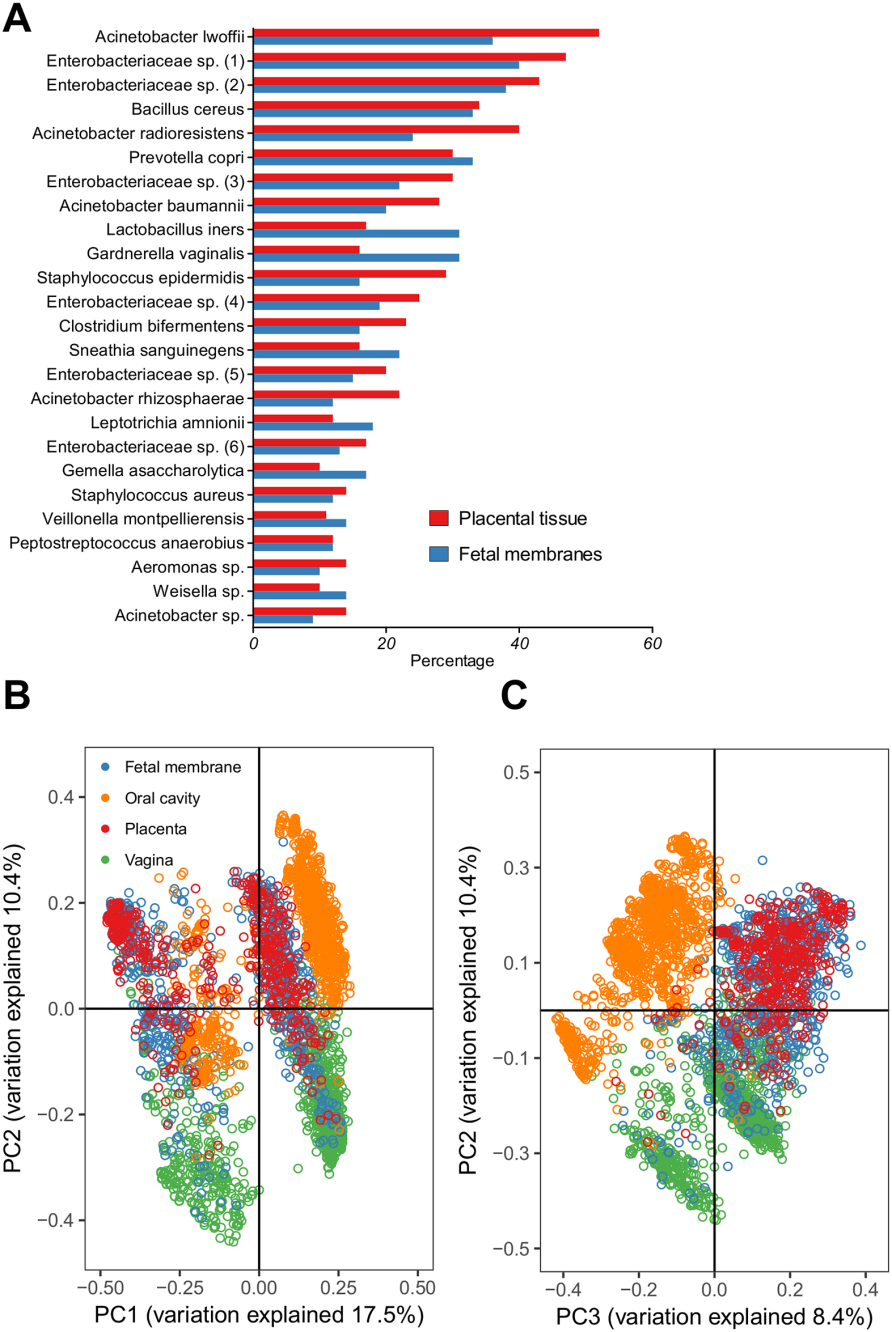
Identification of a core, distinct placental microbiome. (A) Rank abundance curve showing the 25 most common organisms recovered from participants’ placental tissue (n = 476) and fetal membranes (n = 738) that were positive for bacterial DNA. (B) Principal coordinate analysis of unweighted UniFrac distances computed for matched participant placenta (n = 445), fetal membrane (n = 719), oral (n = 725) and vaginal (n = 747) samples from 1107 participants. PC1 and PC2 refer to principal coordinate two and principal coordinate three respectively. Each individual point refers to a single participant’s microbiome for that body site, with samples in similar positions on each axis assumed to have similar microbiomes and samples further apart in the plot are assumed to have more divergent microbiomes. (C) Principal coordinate analysis of unweighted UniFrac distances using the same participants as (B) but plotting axes PC2 and PC3.

Fig 3A shows, across the population analysed, the 25 commonest organisms detected within placental tissues. Bacterial patterns were similar in fetal membranes and placental tissue. Fetal membranes had a higher incidence of *Lactobacillus iners*, *Gardnerella vaginalis* and *Sneathia sanguinegens* whereas the placental tissues had higher incidences of *Acinetobacter* spp. and *Enterobacteriaceae* spp. We compared UniFrac distances between individual’s microbiomes from each body site. The UniFrac distance would be greater if fewer OTUs were shared between each individual and smaller if both communities contained the same OTUs. The OTUs detected within the placental and fetal membrane tissues were indistinguishable, suggesting in many cases a large overlap of similar taxa (Fig 3B and 3C). Comparison of bacteria in placental and fetal tissues with bacteria in vaginal and oral samples from the same individuals revealed some overlap with the microbiota within the vagina, but very little overlap with the microbiota from the oral cavity (Fig 3B and 3C). We found that principal components two and three distinguished samples by body site relatively accurately. The placental and fetal membrane microbial communities had high variability when compared to the vaginal and oral microbiomes. Intra-individual unweighted UniFrac distances in the placenta and fetal membranes were higher than in the oral cavity and vagina. Placenta and fetal membranes had a mean (SD) UniFrac distance across all individuals of 0.69 (0.06) and 0.71 (0.06) respectively, compared to 0.50 (0.09) in the oral cavity and 0.59 (0.11) in the vagina.

### Variation in the placenta microbiome is determined by the abundance of a limited range of species

**Fig 4:**
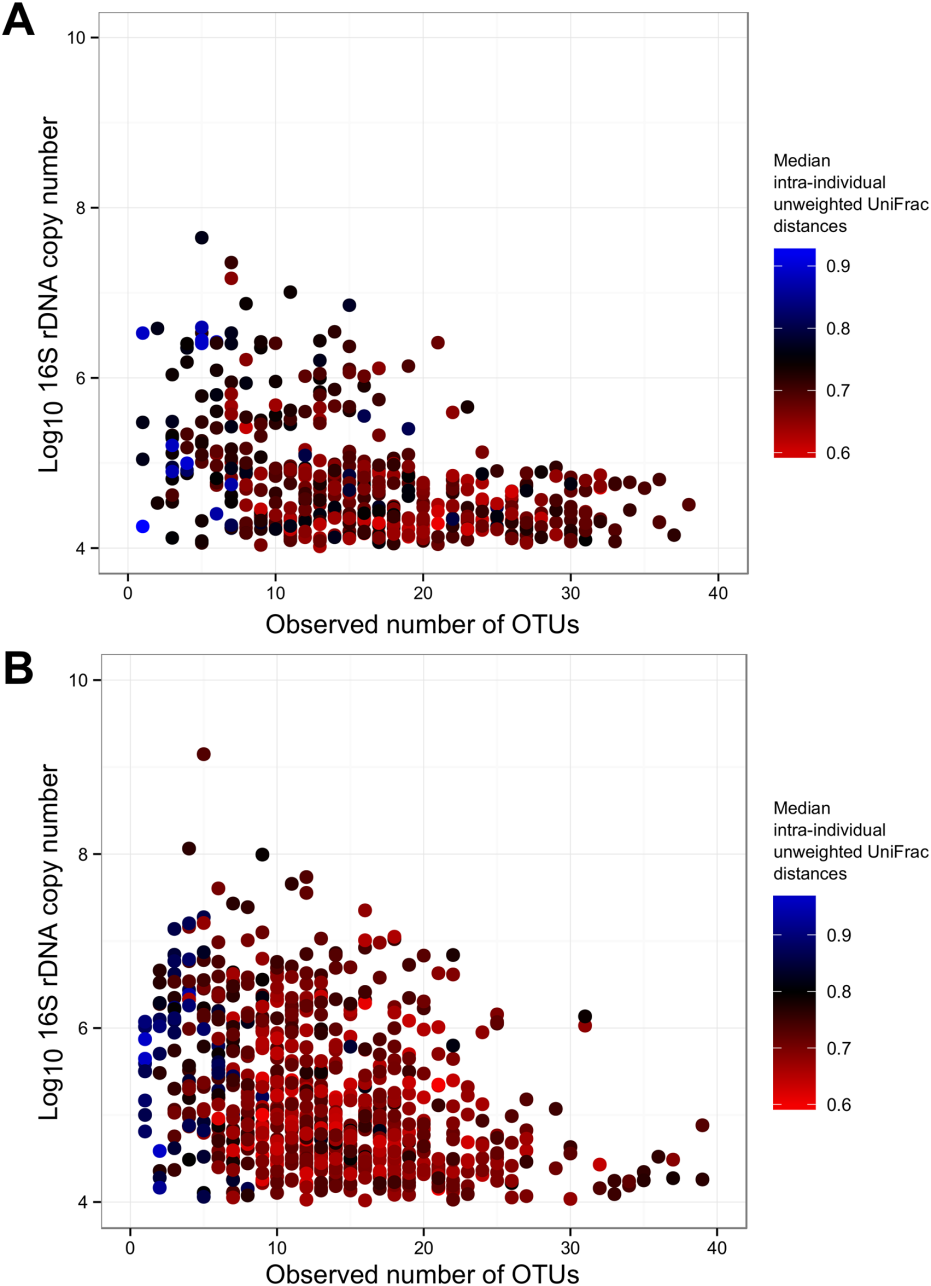
High bacterial load in placental tissues associated with a restricted number of phylogenetically diverse organisms. Multivariate plot of participant’s (A) placental and (B) fetal membrane bacterial load, observed number of OTUs and median intra-individual unweighted UniFrac distances.

Fig 4 shows that higher bacterial loads, as determined by qPCR, were observed when there were fewer OTUs, as determined by 16S rDNA amplicon sequencing. In the placental tissue, a drop in observed OTUs of one was associated with a mean (95%CI) increase in bacterial load of 0·03 (0·03, 0·04) log 10 16S rDNA copies / μl, p<0·001. A decrease in observed OTUs of one in fetal membranes was associated with a mean (95%CI) increase in bacterial load of 0·04 (0·03, 0·05), p<0·001. This indicates that a high bacterial load is linked to the expansion of a limited number of organisms. However, the high median intra-individual unweighted UniFrac distances indicate that the composition of the microbial communities is highly variable between individual women. A decrease in observed OTUs of one in placental tissue was also associated with a rise of the mean (95%CI) intra-individual unweighted UniFrac distances of 0·02 (0·02, 0·03), p<0·001 (Fig 4A). In fetal membranes a decrease in observed OTUs of one was associated with a rise of the mean intra-individual unweighted UniFrac distance of 0·03 (0·03, 0·04), p<0·001 (Fig 4B).

### Specific combinations of bacteria associate with each other

**Fig 5:**
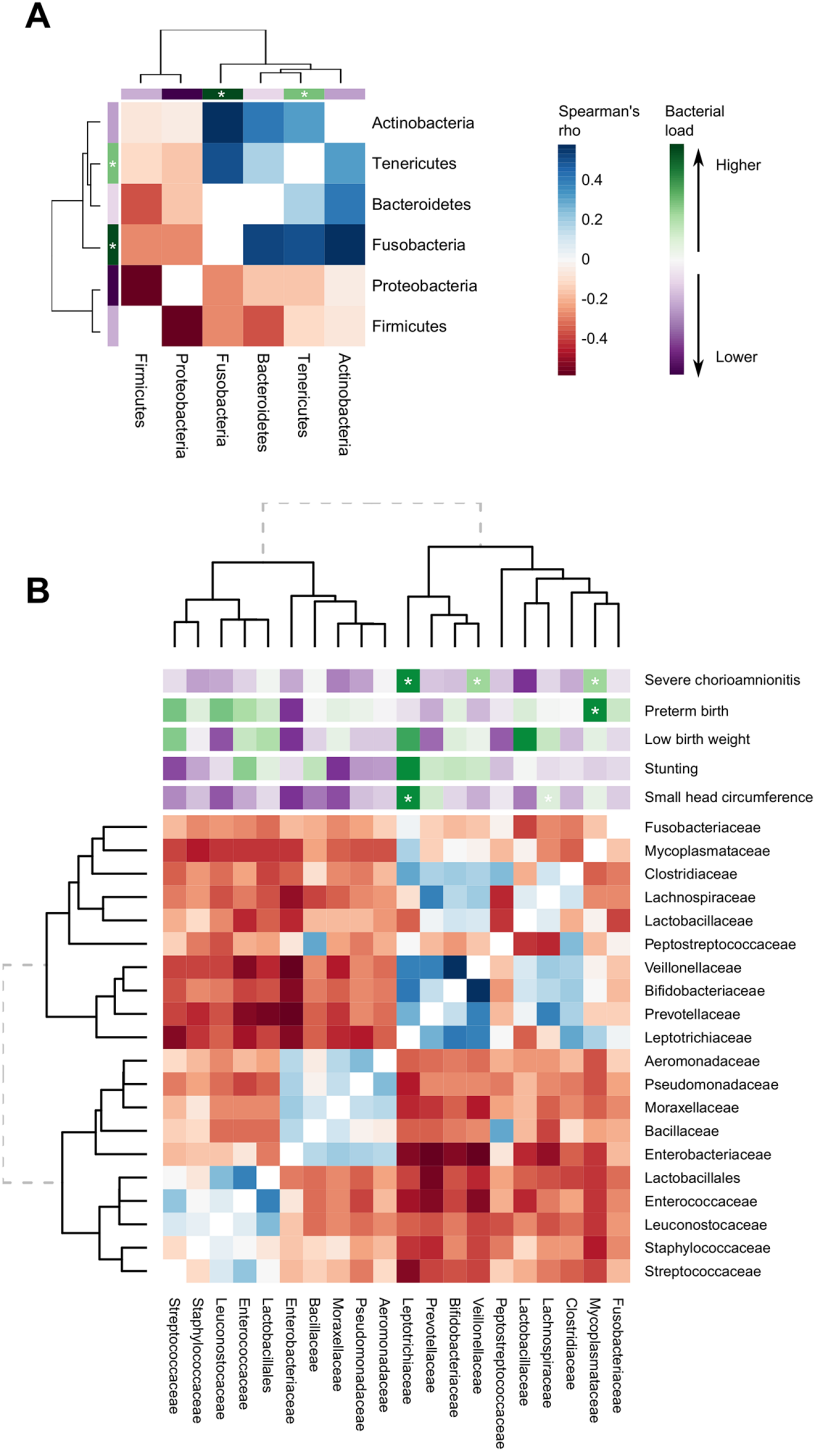
Specific combinations of bacteria found in fetal membranes that associate with each other, severe chorioamnionitis and adverse birth outcomes. Heat map of Spearman’s correlations between the 6 most abundant bacterial phyla with each other (A) and 20 most abundant bacterial families with each other (B) recovered from fetal membranes. Hierarchical clustering was computed by complete linkage of Euclidean distances. Heat map is annotated with mean difference in bacterial load between participants with and without severe chorioamnionitis, preterm birth (<37 weeks), low birth weight (<2500g) and neonatal stunting (LAZ < -2) and small head circumference (HCZ < -2) for each bacterial phyla or family. Asterisks indicate p<0·05 association between higher load of that bacterial phyla or family and prevalence of severe chorioamnionitis, preterm birth, low birth weight, neonatal stunting or small head circumference. The mean different and confidence intervals of these associations can be found in the main results text. Adjusted model *P* values were calculated using linear regression adjusting for the nutritional intervention, maternal BMI at enrolment, maternal age, proxy for socioeconomic status, number of previous pregnancies, anaemia, site of enrolment, mode of delivery and time between delivery and placenta sampling.

The fetal membrane microbial community structure was probed using the associations between the abundances of different OTUs. The abundances of a distinct group of phyla containing Fusobacteria, Tenericutes, Bacteroidetes and Actinobacteria were found to be positively correlated (Fig 5A). Proteobacteria and Firmucutes were rarely found together and were negatively correlated with all other phyla. To explore these interactions in more detail we examined correlations among the 20 most abundant bacterial families and using hierarchical clustering this split the taxa into two major groups (Fig 5B). One group, in which *Fusobacteriaceae*, *Mycoplasmataceae*, *Bifidobacteriaceae*, *Prevotellaceae* and *Leptotrichiaceae* abundances clustered together, matched the same correlations seen at the phyla level. However, the group also included many families from the Firmicutes phyla such as *Clostridiaceae*, *Lachnospiraceae*, *Lactobacillaceae* and *Peptostreptococcaceae*. The second group of organisms that clustered together included a group of families from the phyla Firmicutes that included *Streptococcaceae*, *Staphylococcaceae*, *Leuconostocaceae*, *Enterococcaceae* and *Lactobacillales*, whose abundances were positively correlated with each other. The other organisms in that cluster were overwhelmingly from the phyla Proteobacteria and included *Enterobacteriaceae*, *Aeromonadaceae*, *Pseudomonadaceae* and *Moraxellaceae*. Abundances for all families in the phyla Proteobacteria were positively correlated with each other and with the abundance of *Bacillaceae*. Similar patterns were seen in full-thickness placental tissue samples at both the phylum and family level (S2 Fig).

### Specific combinations of bacteria associate with severe chorioamnionitis

**Fig 6:**
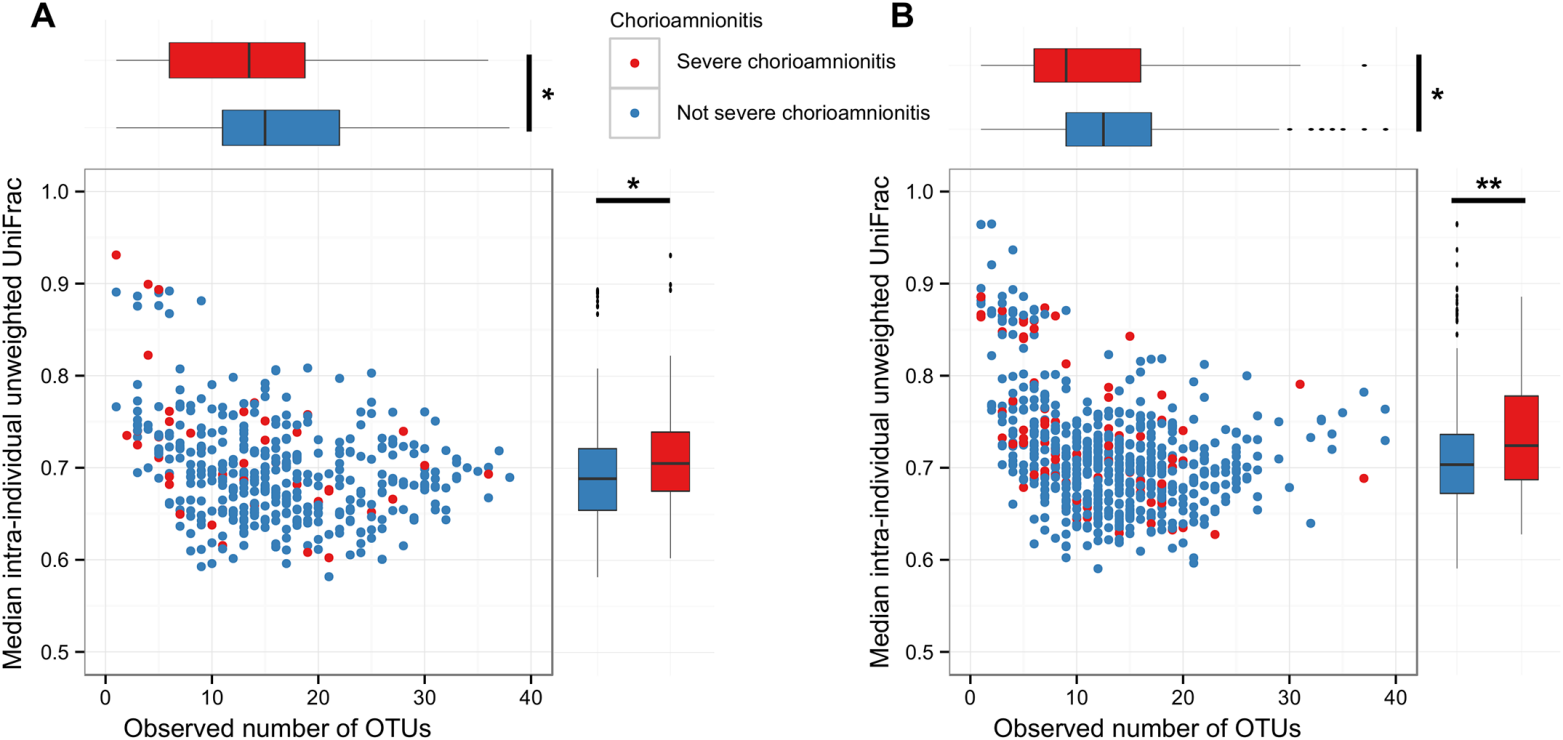
Severe chorioamnionitis is associated with lower species richness and higher phylogenetic diversity. Comparison between observed number of OTUs and median intra-individual unweighted UniFrac distances in placental tissue (A) and fetal membranes (B) and presence of severe chorioamnionitis. Adjusted model *P* values were calculated using linear regression adjusting for the intervention, maternal BMI at enrolment, maternal age, proxy for socioeconomic status, number of previous pregnancies, anaemia, site of enrolment, mode of delivery and time between delivery and placenta sampling (*p<0.05, **p<0.01).

A total of 258 (26·1%) participants had histologically determined chorioamnionitis with 120 (12·1%) participants classified as having severe chorioamnionitis (Table 2). Presence of chorioamnionitis was not significantly associated with birth outcomes, however, participants with severe chorioamnionitis delivered on average (95%CI) -0·4 (-0.8,-0·1) gestational weeks earlier (p = 0·019) than those without severe chorioamnionitis. Those with severe chorioamnionitis also had a lower observed number of OTUs in the placenta (p = 0·029, Fig 6A) and fetal membranes (p = 0·025, Fig 6B) and increased intra-individual unweighted UniFrac distances in the placenta (p = 0·034, Fig 6A) and fetal membranes (p = 0·003, Fig 6B).

**Table 2:** Placental examination of chorioamnionitis and sever chorioamnionitis (n = 989).

Characteristic	With (%)	Without (%)
≥5 neutrophil granulocytes on average per 10 high power fields present in either the chorionic plate or the amniotic membrane (Chorioamnionitis)	258 (26.1)	731 (73.9)
≥25 neutrophil granulocytes on average per 10 high power fields present in either the chorionic plate or the amniotic membrane (Severe chorioamnionitis)	120 (12.1)	869 (87.9)

Severe chorioamnionitis was also associated with distinct phyla. A higher difference between mean loads of Fusobacteria in fetal membranes of participants with severe chorioamnionitis compared to no chorioamnionitis of 0·41 (0·17, 0·64) Log10 16S rDNA copies/μl (p = 0·001) and higher mean Tenericutes load of 0·18 (0·05, 0·30) Log10 16S rDNA copies/μl (p = 0·006) (Fig 5A). This was also reflected at a family level with a significantly higher mean bacterial load of *Mycoplasmataceae* (p = 0·010), *Leptotrichiaceae* (p = 0·001), and *Veillonaceae* (p = 0·001) in placental tissues with severe chorioamnionitis (Fig 5B). Abundances of *Mycoplasmataceae*, *Leptotrichiaceae*, and *Veillonaceae* were also all positively intercorrelated.

### Microbial composition recovered from fetal membranes is associated with birth outcomes

31 OTUs from fetal membranes were identified whose abundances were positively correlated with or belonging to bacterial families that were significantly associated with adverse birth outcomes (denoted with an asterisk in Fig 5B). Comparisons between bacterial loads of individual OTUs adjusted for bacterial load and 16S rRNA copy number showed that *Fusobacterium nucleatum*, *Ureaplasma sp*. and *Gemella asaccharolytica* were associated with a shorter duration of pregnancy (S5 Table). Higher loads of *Sneathia sanguinegens*, *Prevotella copri*, *Lachnospiraceae* sp. and *Phascolarctobacterium succinatutens* were all significantly associated with smaller newborn size (Table 2, S6 and S7 Tables). After controlling for the false discovery rate, a higher mean bacterial load of an unidentified *Lachnospiraceae* sp., *Sneathia sanguinegens* and *Phascolarctobacterium succinatutens* were significantly associated with a lower newborn head circumference-for-age z-score (Table 3).

**Table 3:** OTUs isolated from fetal membranes significantly associated with differences in head circumference-for-age Z-score.

			Unadjusted analysis			Adjusted analysis		
O.T.U ID (Custom database and greengenes)	Greengenes Taxonomy	Genbank BLASTN result	Correlation coefficient	*P* value^1^	N	Regression coefficient (95%CI)	*P* value^2^	*q* value
2386814	f__Lachnospiraceae; g__; s__	*Lachnospiraceae sp*.	-0.11	0.001	951	-0.4 (-0.6, -0.2)	0.000	0.003
302279	g__Phascolarctobacterium; s__	*Phascolarctobacterium succinatutens*	-0.09	0.004	951	-0.2 (-0.3, -0.1)	0.002	0.031
645321357	g__Sneathia; s__	*Sneathia sanguinegens*	-0.11	0.001	951	-0.1 (-0.1, -0.0)	0.002	0.031
288932	g__Prevotella; s__copri	*Prevotella copri*	-0.08	0.014	951	-0.1 (-0.2, -0.0)	0.031	0.320
631251895	g__Prevotella; s__	*Prevotella amnii*	-0.06	0.007	951	-0.1 (-0.2, 0.0)	0.064	0.496

^1^ P value calculated using Pearson’s correlation.

^2^ Adjusted p-values were calculated using linear regression models. Regression coefficient shows the change in head circumference-for-age Z-score in relation to an increase in bacterial load of one Log10 genome/μl. All models were adjusted for the intervention, maternal BMI at enrolment, maternal age, proxy for socioeconomic status, number of previous pregnancies, anaemia, site of enrolment, mode of delivery and time between delivery and placenta sampling.

### The source of bacteria in placental tissues

**Fig 7:**
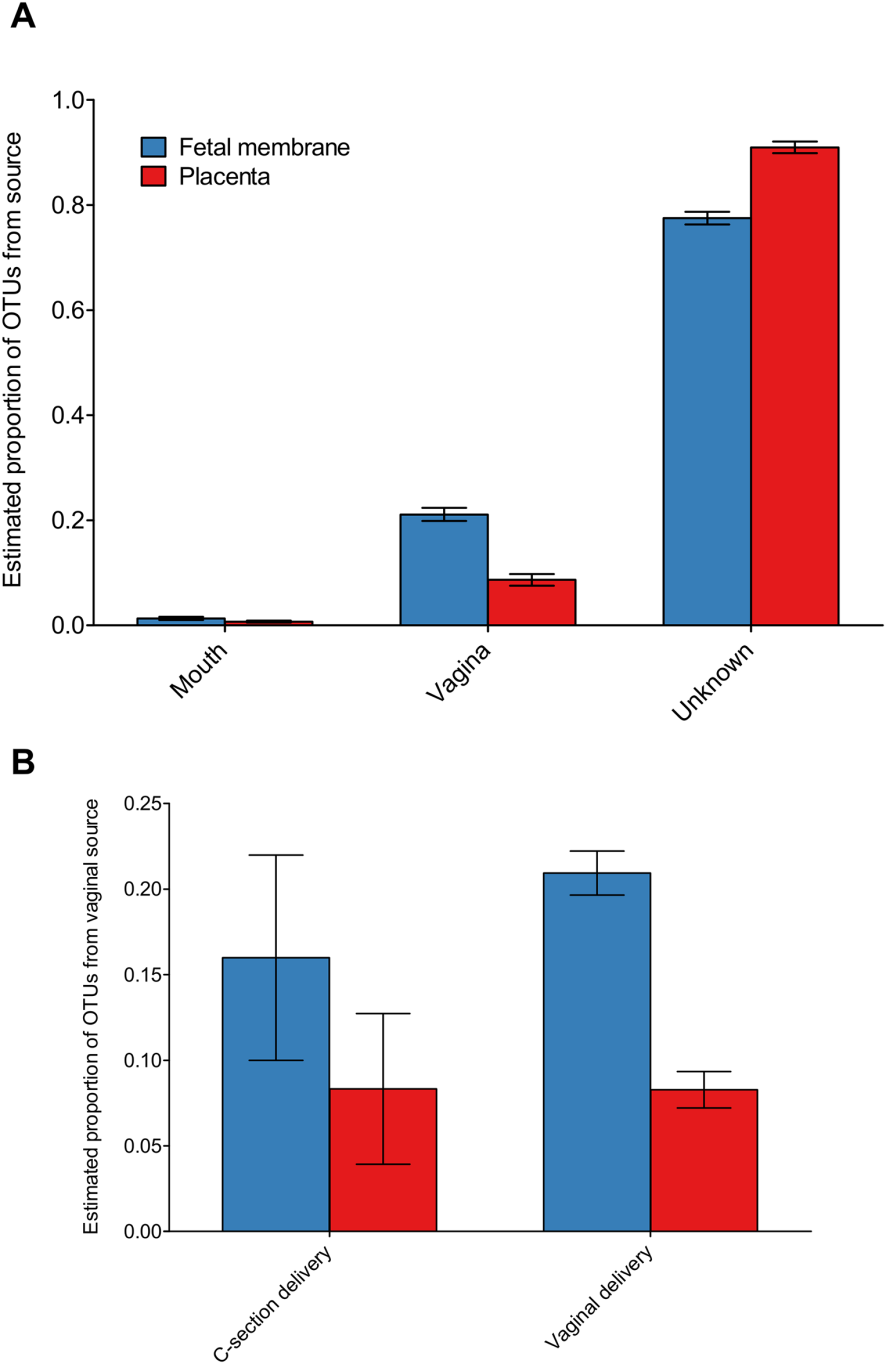
OTUs identified in placental tissues are also identified in participant’s mouth and vagina. (A) The mean ± SEM estimated proportion of OTUs in placenta and fetal membranes sourced from vaginal, oral cavity or an unknown source. (B) Comparison between the proportion of OTUs found in placenta and fetal membranes sourced from the vagina stratified by participants who delivered vaginally or by caesarean section.

SourceTracker [23] was used to identify the likely source for the bacteria detected in the placental and fetal membrane tissues. We identified 20 OTUs that were detectable from at least one participant’s placental tissue or fetal membrane and were also present in a matched individual sample from either the vagina or oral cavity. We compared their abundances with the proportion of OTUs identified from SourceTracker as being from either the vagina or the oral cavity. 16 OTUs were positively associated with only a single source site using SouceTracker’s predictions, with 14 OTUs from the vagina and two from the oral cavity (S3 Fig). Four bacteria could not be positively correlated with either source and so were not taken forward into subsequent analysis. A larger proportion of OTUs were identified as being sourced from the vagina than from the oral cavity (Fig 7A). Also, similar proportions of organisms from the vagina were found in vaginally delivered and C-section delivered placenta and fetal membranes (Fig 7B). A high proportion of organisms found within the placenta and fetal membranes did not have an obvious source (Fig 7A).

**Fig 8:**
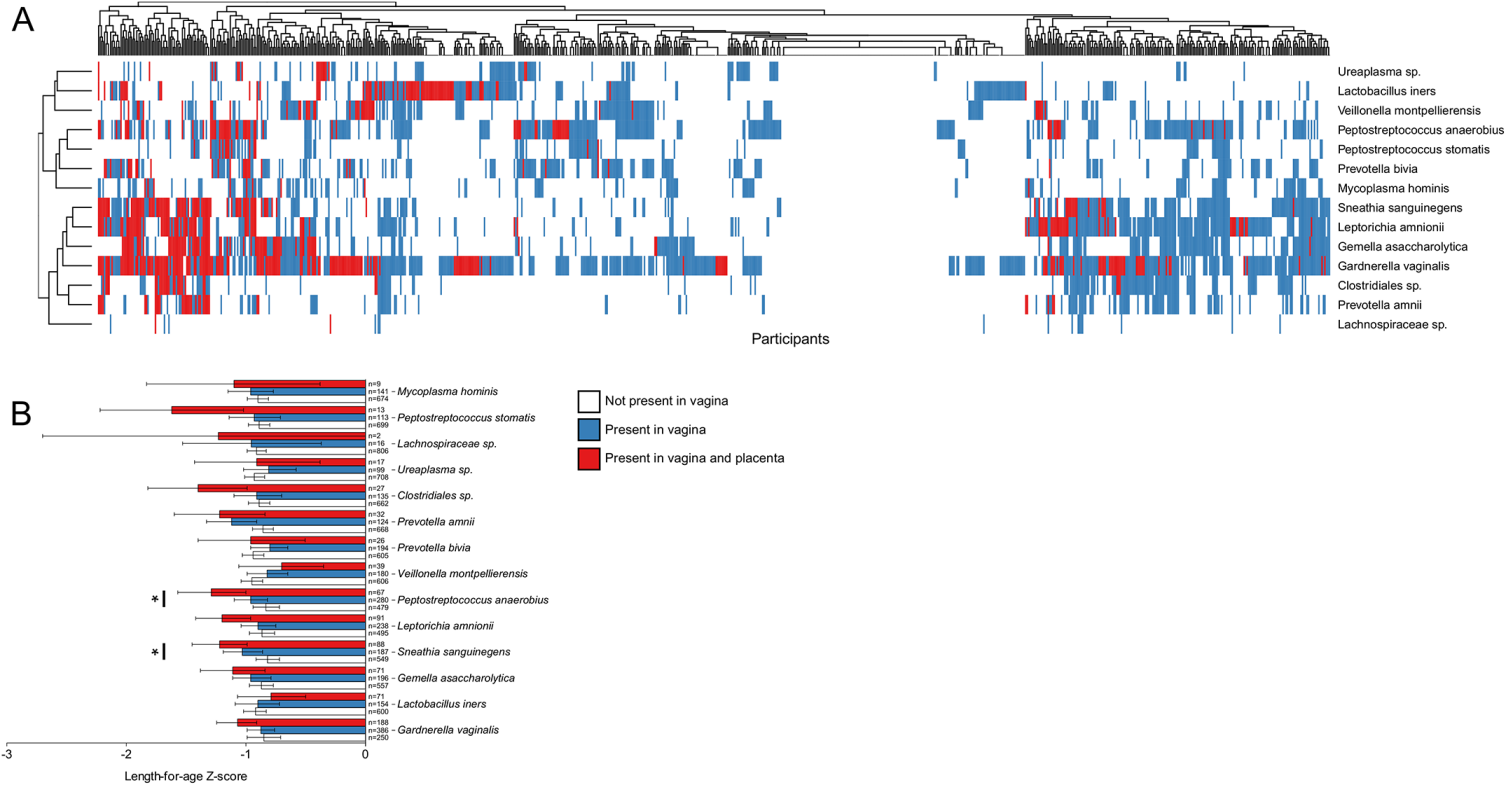
Presence of OTUs in both vagina and placental tissue associated with a lower length-for-age z-score. (A) Heat map showing presence of vaginal organisms in all individuals’ vaginal and placental samples. Hierarchical clustering was computed using average linkage of Euclidean distances. (B) Mean ± 95%CI LAZ score for participants with individual bacterium not present in their vagina, present in their vagina only and present in both vagina and placental tissue (*q<0.05).

Participants were hierarchically clustered by presence of the 14 vaginally sourced OTUs within either their placenta or fetal membrane tissues (Fig 8A). In one cluster, participants had evidence of multiple OTUs present in the vagina and in the placenta. In a second cluster participants had no evidence of these organisms within the vagina or placenta and a third cluster consisted of participants who had a combination of these 14 organisms in the vagina but not in the placenta. Individuals were ranked by whether they had a detectable presence of these OTUs in their vagina and placenta and the mean LAZ score was compared for each group (Fig 8B). The mean LAZ score was lowest in individuals when the organisms were present in both the vagina and placenta, with the highest mean LAZ score when the organisms were not present at all. This trend was statistically significant when *Sneathia sanguinengens* and *Peptostreptococcus anaerobius* were detected in the vagina and placental tissues (q<0.05). There were no significant associations between the presence of any of the same organisms and duration of pregnancy, birth weight or neonatal head circumference.

## Discussion

This is the largest study to date of placental microbiota with over 1000 individuals with tissue available. We detected bacterial DNA in more than 50% of placental tissues and the core microbiome was distinct from the oral and vaginal microbiome. The structures of these placental microbial communities were altered in individuals with severe chorioamnionitis. These altered taxa were predominantly sourced from the vagina and were associated with adverse birth outcomes.

In contrast to our results, the microbial composition of 320 placental samples in another 16S rRNA gene sequencing study was shown to resemble that found on the tonsils and tongue rather than the vagina [8]. The genera that were altered between term and preterm delivery (*Burkholderia*, *Anaeromyxobacter*, *Streptosporangium* and *Roseovarius*), have not been previously associated with preterm birth and are recognised environmental organisms that have been associated with reagent contamination of sequencing studies [24].

Samples containing low levels of bacteria, such as placental tissue, are incredibly vulnerable to bacterial DNA contamination during processing, which can lead to the erroneous conclusion that contaminating bacteria were present in the original placental sample. We have optimised our 16S rRNA gene PCR assay for use in clinical microbiology settings, and adjusted the assays to be able to detect a lower limit of 10–100 cfu /μl [6,25]. This is less sensitive than some other study methodologies, and largely explains why bacterial DNA was not detected in more than 50% of full-thickness placental tissue sections. Another study focusing on placentas from healthy deliveries also concluded that the placental microbiome could not be differentiated from laboratory reagent contamination [26], therefore further attempts at sequencing these negative samples would only result in an increase in the detection of contaminating bacterial species.

Due to this study taking place in a low-income setting, there were many challenges in the collection of clinical data and biological samples. These were anticipated, and where possible, the impact of confounders was limited by instituting a number of procedural steps to optimise the chances of detecting bacteria on placental tissues deposited prior to or at delivery. These included sampling and freezing tissues as soon after delivery as possible, and even in remote settings this was relatively successful (see Fig 2). Similarly, placental samples used for histological analysis were fixed in formalin at the same time as sampling before storage at +4°C until embedded in paraffin and used for histology. We ensured data collection quality through detailed visit guides, regular staff training and monitoring and instructions about the use of data collection forms. Anthropometric measurements were taken only by trained personnel (birth weight could also be measured by study nurses or study coordinators) whose measurement reliability was verified at the start of the study and at 6-month intervals thereafter. All anthropometry equipment was calibrated daily and an external monitor appointed by the study team did one site monitoring visit during data collection.

In spite of the procedures instituted to minimise microbial contamination, the majority of placental tissues in this study contained OTUs that were not found in either matched participant vaginal or oral samples. It is likely that these reflect contaminating bacteria form the environment and maternal faeces. We therefore focused on individuals that had a relatively small number of phylogenetically diverse OTUs that were also found in matched vaginal samples and were associated with both severe chorioamnionitis and adverse birth outcomes. It seems likely that these features represent ‘true’ infection of the placental tissue.

Interestingly, we found high abundances of *Fusobacterium nucleatum* and *Ureaplasma* spp. that were inversely correlated with duration of pregnancy. This finding was reported in another study using similar techniques [27]. These species have also frequently been found in the amniotic fluid of women who deliver preterm [28–30], indicating a pathogenic role in preterm birth. Recent molecular studies have found *Sneathia sanguinegens*, *Prevotella* spp., *Peptostreptococcus* spp. and *Gardnerella vaginalis* in both amniotic fluid and placental tissues of women who have delivered prematurely [6,31] and is consistent with our results. These organisms were not found in the study by Prince et al [27], which may reflect that the larger size of our study, (1097 compared to 71 participants), can identify even a low prevalence of potentially pathogenic bacteria. Importantly, several OTUs associated with a shorter duration of pregnancy and smaller newborn size in our study have been previously associated with bacterial vaginosis such as *Gardnerella* spp., *Ureaplasma* spp., *Sneathia* spp., *Prevotella* spp. and *Lachnospiraceae* spp. [32,33]. This includes a recent study that found a higher abundance of *Gardnerella* spp. and *Ureaplasma* spp. in the vaginas of women during pregnancy who subsequently delivered preterm [34].

Further evidence that the approaches we have taken to identify potentially pathogenic bacterial colonisation/infection comes from finding comparable levels of bacteria from the placental tissue and vagina in both caesarean sections and vaginally delivered samples. Our data supports the origin of pathogenic bacteria detected in placental tissue as originating from the vagina. Using samples from the same participant we could show OTUs on placental tissue were of vaginal or oral origin. When *Peptostreptococcus anaerobious and Sneathia sanguinegens* were found in both the vagina and placenta, this was associated with smaller newborn size. This provides further evidence that ascending infection, and hence the vaginal microbiome, may play a role in birth outcomes. It is not possible to exclude a role for the oral microbiome in seeding placental tissue [35], as we did find the same OTUs in the oral cavity and placenta in some individuals. Furthermore, the oral sample collected in this study was a swab of the gingival margins and may not detect bacteria found in deep periodontal pockets or periapical tissues that could have an association with adverse birth outcomes in this cohort [36,37].

Inevitably with a study of this type and in this setting, there are a number of potential limitations in addition to contamination already discussed. Sampling of the oral and vaginal samples occurred one week after the delivery. Bacteria in the vaginal and oral cavities may be influenced by changes occurring in human microbiomes immediately after delivery, especially in the vaginal tract [34]. This may impact on any associations with placental microbiota and birth outcomes. We also recognise that using relative abundances adjusted for 16S qPCR load is not a standard approach. We developed this approach because as many placental samples did not have a detectable resident microbiome, comparing just relative abundances would be skewed by samples that were negative or of low diversity. While this approach cannot correct the intrinsic biases using these PCR-based methodologies, in our view it remains the best available method for analysis of samples with a low bacterial load.

There are no comparable data from Africa, but the organisms we detected are similar to those identified in amniotic fluid [38,39] or placental tissues [7,40] from studies across Europe and North America. This may indicate that placental microbiome we observed may be relatively preserved in multiple geographical settings. It may also provide a rationale for treating pregnant women with antibiotics. A trial in Malawi prospectively treated women with antibiotics during pregnancy showed a protective effect on the incidence of preterm birth and low birth weight [41]. This could be explained by the clearing of potential pathogens responsible for ascending infection that lead to preterm birth or intrauterine growth restriction. Other microbiomes, such as that of the gut, are known to differ significantly between western and African populations [42] and we found a minority of OTUs likely to originate from the GI tract, such as a *Blautia sp*., *Phascolarctobacterium succinatutens* and a *Lachnospiraceae sp*. that have not previously been associated with adverse birth outcomes. It is unknown at the moment whether these are faecal contaminants or reflect regional differences between this study and previous studies.

In Malawi approximately 21% of pregnant women carry group B streptococcus (GBS) [43] and has also been found to involved with placental infection and chorioamnionitis [44,45]. GBS was not identified in this cohort by our methods. This may be due to a limitation in species identification of short reads sequenced from the 16S rRNA gene, rather than a genuine lack of GBS in this study cohort. We found approximately 10% of participants had unidentified Streptococcus spp. sequences that did not have enough variation between them to confidently assign a species name but may well be GBS.

In summary, we report the largest study to date to examine the placental microbiome at birth. While the setting rural Malawi was challenging, we have identified a distinct microbial community in the placenta and fetal membranes associated with severe chorioamnionitis and adverse birth outcomes. Bacteria associated with both severe chorioamnionitis and poor birth outcomes were phylogenetically diverse and not the most abundant taxa recovered in the placenta or fetal membranes. Interestingly, the species associated with preterm birth were distinct from those associated with the three measures of growth restriction. Further studies are needed to elucidate mechanisms by which bacteria restrict fetal growth without triggering chorioamnionitis or preterm labour. Strategic control of the microbiome resident in the vagina or oral cavity with proven efficacy against organisms identified in this study could control potential etiologic agents that spread to the placenta. While the use of antibiotics to reduce rates of preterm delivery have had limited success [46,47], targeting bacteria associated with poor birth outcomes may prove more successful. In this respect, there is a logic to using an antibiotic such as clindamycin, and indeed this has been shown to reduce the risk of preterm birth and late miscarriage [48]. Screening of the vaginal flora and/or the presence of cervical inflammation during pregnancy could identify at risk groups who could be targeted for treatment with selective and limited antibiotics or vaginal probiotics to limit adverse birth outcomes in the future.

## Supporting information

S1 Fig Associations between time after delivery the placenta was sampled, birth outcomes and whether a participant delivered at home. Box-and-whisker plots showing the association between the time after delivery the placenta was sampled and prevalence of **(A)** preterm birth, **(B)** low birth weight, **(C)** stunting and **(D)** small-head circumference. As well as the association between the whether the participant delivered at home and the **(E)** observed number of OTUs and **(F)** median inter-individual unweighted UniFrac distance.
(TIFF)

S2 Fig Specific combinations of bacteria found in placental tissues that associate with each other, severe chorioamnionitis and adverse birth outcomes. Heat map of Spearman’s correlations between the 6 most abundant bacterial phyla **(A)** and 20 most abundant bacterial families **(B)** recovered from fetal membranes. Hierarchical clustering was computed by complete linkage of Euclidean distances. Heat map is annotated with mean difference in bacterial load between participants with and without severe chorioamnionitis, preterm birth (<37 weeks), low birth weight (<2500g) and neonatal stunting (LAZ < -2) and small head circumference (HCZ < -2) for each bacterial phyla or family. Asterisks indicate p<0·05 association between higher load of that bacterial phyla or family and prevalence of severe chorioamnionitis. Adjusted model *P* values were calculated using linear regression adjusting for the nutritional intervention, maternal BMI at enrolment, maternal age, proxy for socioeconomic status, number of previous pregnancies, anaemia, site of enrolment, mode of delivery and time between delivery and placenta sampling.
(TIFF)

S3 Fig OTUs identified from either a vaginal or oral source. Heat map of Spearman’s correlations between the relative abundance of an OTU that is present in both the placenta and either the vagina or oral cavity and the estimated proportion of OTUs from either a vaginal or oral source as calculated by SourceTracker (*q<0.05).
(TIFF)

S1 Table Combination of 16S rRNA V5-V7 library preparation primers to multiplex 384 samples. (DOCX)

S2 Table Number of sequenced reads generated from placental and fetal membrane samples. (DOCX)

S3 Table Associations between OTUs isolated from placental tissue and the time between delivery and processing of sample. (DOCX)

S4 Table Associations between OTUs isolated from fetal membranes and the time between delivery and processing of sample. (DOCX)

S5 Table OTUs isolated from fetal membranes significantly associated with differences in duration of pregnancy. (DOCX)

S6 Table OTUs isolated from fetal membranes significantly associated with differences in birth weight. (DOCX)

S7 Table OTUs isolated from fetal membranes significantly associated with differences in length-for-age Z-score. (DOCX)
