# Supplementary figures and images for: Bacterial communities found in placental tissues are associated with severe chorioamnionitis and adverse birth outcomes
Source: PLoS One. 2017 Jul 12;12(7):e0180167. doi: 10.1371/journal.pone.0180167 (PMC5507499; doi:10.1371/journal.pone.0180167)

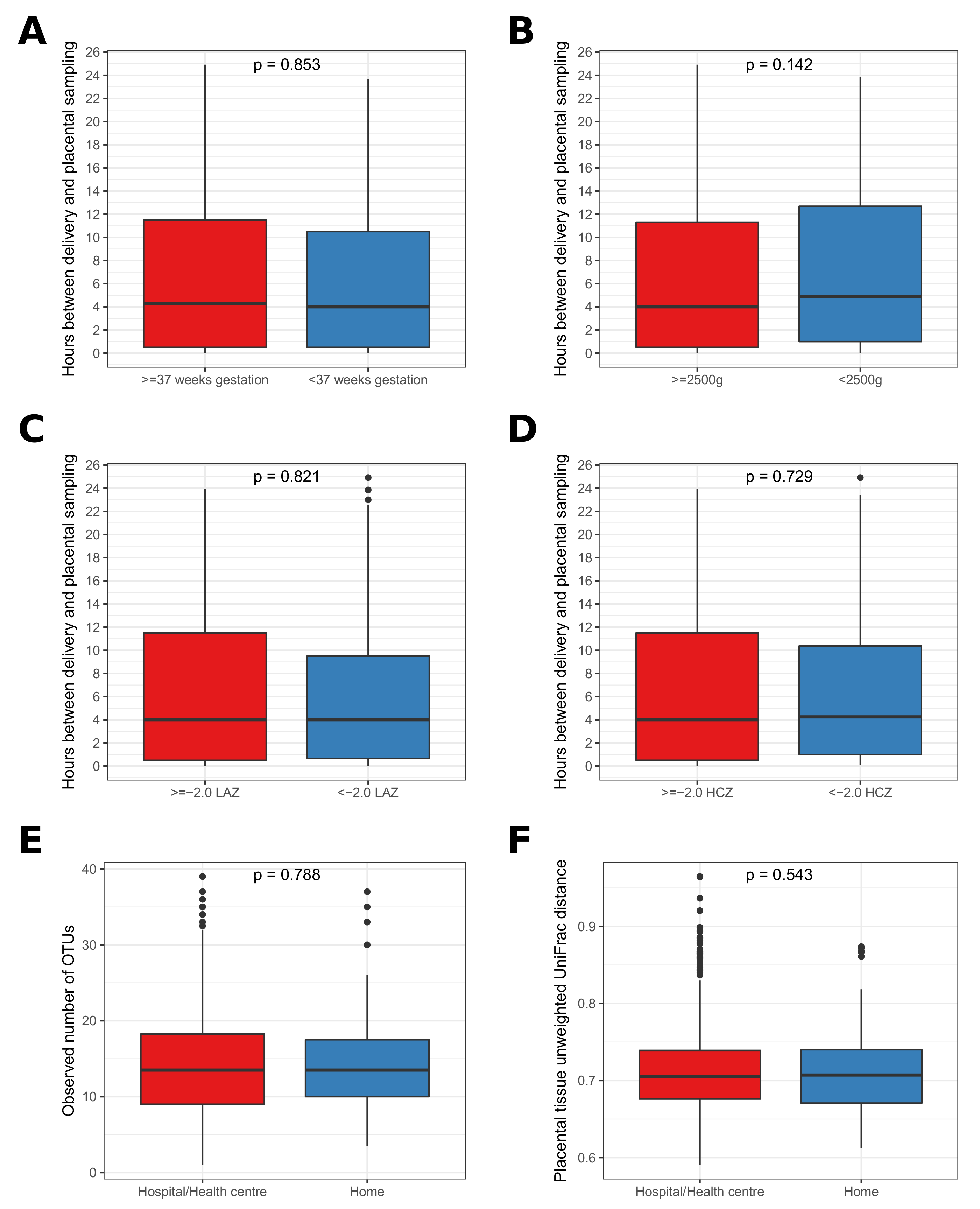

Supplement: S1 Fig — Box-and-whisker plots showing the association between the time after delivery the placenta was sampled and prevalence of (A) preterm birth, (B) low birth weight, (C) stunting and (D) small-head circumference. As well as the association between the whether the participant delivered at home and the (E) observed number of OTUs and (F) median inter-individual unweighted UniFrac distance. (TIFF) [file pone.0180167.s001.tiff]

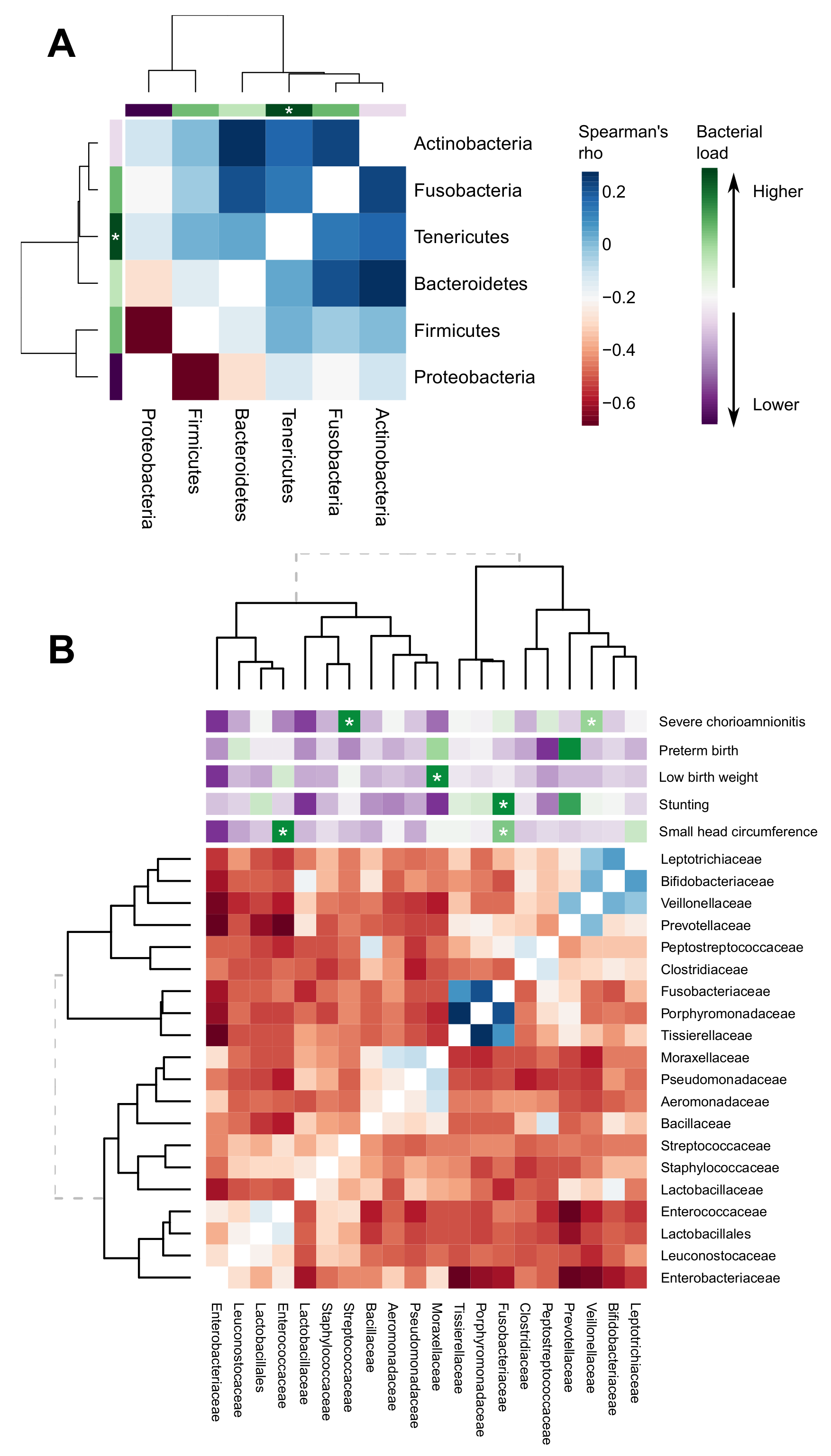

Supplement: S2 Fig — Heat map of Spearman’s correlations between the 6 most abundant bacterial phyla (A) and 20 most abundant bacterial families (B) recovered from fetal membranes. Hierarchical clustering was computed by complete linkage of Euclidean distances. Heat map is annotated with mean difference in bacterial load between participants with and without severe chorioamnionitis, preterm birth (<37 weeks), low birth weight (<2500g) and neonatal stunting (LAZ < -2) and small head circumference (HCZ < -2) for each bacterial phyla or family. Asterisks indicate p<0·05 association between higher load of that bacterial phyla or family and prevalence of severe chorioamnionitis. Adjusted model P values were calculated using linear regression adjusting for the nutritional intervention, maternal BMI at enrolment, maternal age, proxy for socioeconomic status, number of previous pregnancies, anaemia, site of enrolment, mode of delivery and time between delivery and placenta sampling. (TIFF) [file pone.0180167.s002.tiff]

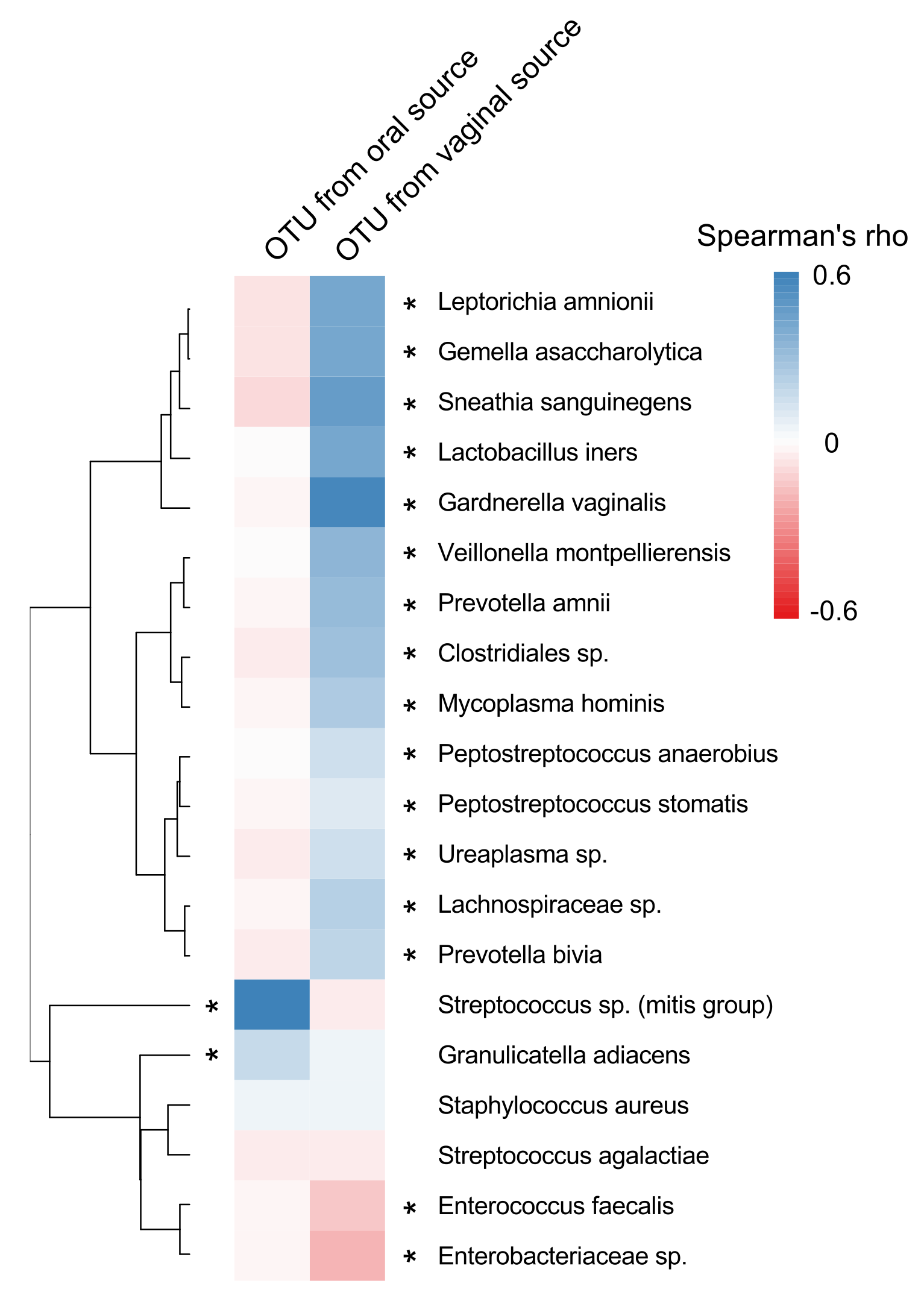

Supplement: S3 Fig — Heat map of Spearman’s correlations between the relative abundance of an OTU that is present in both the placenta and either the vagina or oral cavity and the estimated proportion of OTUs from either a vaginal or oral source as calculated by SourceTracker (*q<0.05). (TIFF) [file pone.0180167.s003.tiff]
