# Supplementary material for: Bacterial communities found in placental tissues are associated with severe chorioamnionitis and adverse birth outcomes
Source: PLoS One. 2017 Jul 12;12(7):e0180167. doi: 10.1371/journal.pone.0180167 (PMC5507499; doi:10.1371/journal.pone.0180167)
Supplement: S1 Table — (DOCX) [file pone.0180167.s004.docx]

**Table S1. Combination of 16S rRNA V5-V7 library preparation primers to multiplex 384 samples**

| **Sample number** | **Reverse primer name** | **Reverse primer sequence** | **Forward primer name** | **Forward primer sequence** |
| --- | --- | --- | --- | --- |
| 1 | Rrcbc32 | CAAGCAGAAGACGGCATACGAGATGCATATGCACTGAACACGTTTTAACGTCRTCCCCDCCTTCCTC | Fbc0 | AATGATACGGCGACCACCGAGATCTACACTCCCTTGTCTCCTACCGGGACTTAGGATTAGATACCCBRGTAGTC |
| 2 | Rrcbc33 | CAAGCAGAAGACGGCATACGAGATCAACTCCCGTGAAACACGTTTTAACGTCRTCCCCDCCTTCCTC | Fbc0 | AATGATACGGCGACCACCGAGATCTACACTCCCTTGTCTCCTACCGGGACTTAGGATTAGATACCCBRGTAGTC |
| 3 | Rrcbc34 | CAAGCAGAAGACGGCATACGAGATTTGCGTTAGCAGAACACGTTTTAACGTCRTCCCCDCCTTCCTC | Fbc0 | AATGATACGGCGACCACCGAGATCTACACTCCCTTGTCTCCTACCGGGACTTAGGATTAGATACCCBRGTAGTC |
| 4 | Rrcbc35 | CAAGCAGAAGACGGCATACGAGATTACGAGCCCTAAAACACGTTTTAACGTCRTCCCCDCCTTCCTC | Fbc0 | AATGATACGGCGACCACCGAGATCTACACTCCCTTGTCTCCTACCGGGACTTAGGATTAGATACCCBRGTAGTC |
| 5 | Rrcbc36 | CAAGCAGAAGACGGCATACGAGATCACTACGCTAGAAACACGTTTTAACGTCRTCCCCDCCTTCCTC | Fbc0 | AATGATACGGCGACCACCGAGATCTACACTCCCTTGTCTCCTACCGGGACTTAGGATTAGATACCCBRGTAGTC |
| 6 | Rrcbc37 | CAAGCAGAAGACGGCATACGAGATTGCAGTCCTCGAAACACGTTTTAACGTCRTCCCCDCCTTCCTC | Fbc0 | AATGATACGGCGACCACCGAGATCTACACTCCCTTGTCTCCTACCGGGACTTAGGATTAGATACCCBRGTAGTC |
| 7 | Rrcbc38 | CAAGCAGAAGACGGCATACGAGATACCATAGCTCCGAACACGTTTTAACGTCRTCCCCDCCTTCCTC | Fbc0 | AATGATACGGCGACCACCGAGATCTACACTCCCTTGTCTCCTACCGGGACTTAGGATTAGATACCCBRGTAGTC |
| 8 | Rrcbc39 | CAAGCAGAAGACGGCATACGAGATTCGACATCTCTTAACACGTTTTAACGTCRTCCCCDCCTTCCTC | Fbc0 | AATGATACGGCGACCACCGAGATCTACACTCCCTTGTCTCCTACCGGGACTTAGGATTAGATACCCBRGTAGTC |
| 9 | Rrcbc40 | CAAGCAGAAGACGGCATACGAGATGAACACTTTGGAAACACGTTTTAACGTCRTCCCCDCCTTCCTC | Fbc0 | AATGATACGGCGACCACCGAGATCTACACTCCCTTGTCTCCTACCGGGACTTAGGATTAGATACCCBRGTAGTC |
| 10 | Rrcbc41 | CAAGCAGAAGACGGCATACGAGATGAGCCATCTGTAAACACGTTTTAACGTCRTCCCCDCCTTCCTC | Fbc0 | AATGATACGGCGACCACCGAGATCTACACTCCCTTGTCTCCTACCGGGACTTAGGATTAGATACCCBRGTAGTC |
| 11 | Rrcbc42 | CAAGCAGAAGACGGCATACGAGATTTGGGTACACGTAACACGTTTTAACGTCRTCCCCDCCTTCCTC | Fbc0 | AATGATACGGCGACCACCGAGATCTACACTCCCTTGTCTCCTACCGGGACTTAGGATTAGATACCCBRGTAGTC |
| 12 | Rrcbc43 | CAAGCAGAAGACGGCATACGAGATAAGGCGCTCCTTAACACGTTTTAACGTCRTCCCCDCCTTCCTC | Fbc0 | AATGATACGGCGACCACCGAGATCTACACTCCCTTGTCTCCTACCGGGACTTAGGATTAGATACCCBRGTAGTC |
| 13 | Rrcbc32 | CAAGCAGAAGACGGCATACGAGATGCATATGCACTGAACACGTTTTAACGTCRTCCCCDCCTTCCTC | Fbc1 | AATGATACGGCGACCACCGAGATCTACACACGAGACTGATTTACCGGGACTTAGGATTAGATACCCBRGTAGTC |
| 14 | Rrcbc33 | CAAGCAGAAGACGGCATACGAGATCAACTCCCGTGAAACACGTTTTAACGTCRTCCCCDCCTTCCTC | Fbc1 | AATGATACGGCGACCACCGAGATCTACACACGAGACTGATTTACCGGGACTTAGGATTAGATACCCBRGTAGTC |
| 15 | Rrcbc34 | CAAGCAGAAGACGGCATACGAGATTTGCGTTAGCAGAACACGTTTTAACGTCRTCCCCDCCTTCCTC | Fbc1 | AATGATACGGCGACCACCGAGATCTACACACGAGACTGATTTACCGGGACTTAGGATTAGATACCCBRGTAGTC |
| 16 | Rrcbc35 | CAAGCAGAAGACGGCATACGAGATTACGAGCCCTAAAACACGTTTTAACGTCRTCCCCDCCTTCCTC | Fbc1 | AATGATACGGCGACCACCGAGATCTACACACGAGACTGATTTACCGGGACTTAGGATTAGATACCCBRGTAGTC |
| 17 | Rrcbc36 | CAAGCAGAAGACGGCATACGAGATCACTACGCTAGAAACACGTTTTAACGTCRTCCCCDCCTTCCTC | Fbc1 | AATGATACGGCGACCACCGAGATCTACACACGAGACTGATTTACCGGGACTTAGGATTAGATACCCBRGTAGTC |
| 18 | Rrcbc37 | CAAGCAGAAGACGGCATACGAGATTGCAGTCCTCGAAACACGTTTTAACGTCRTCCCCDCCTTCCTC | Fbc1 | AATGATACGGCGACCACCGAGATCTACACACGAGACTGATTTACCGGGACTTAGGATTAGATACCCBRGTAGTC |
| 19 | Rrcbc38 | CAAGCAGAAGACGGCATACGAGATACCATAGCTCCGAACACGTTTTAACGTCRTCCCCDCCTTCCTC | Fbc1 | AATGATACGGCGACCACCGAGATCTACACACGAGACTGATTTACCGGGACTTAGGATTAGATACCCBRGTAGTC |
| 20 | Rrcbc39 | CAAGCAGAAGACGGCATACGAGATTCGACATCTCTTAACACGTTTTAACGTCRTCCCCDCCTTCCTC | Fbc1 | AATGATACGGCGACCACCGAGATCTACACACGAGACTGATTTACCGGGACTTAGGATTAGATACCCBRGTAGTC |
| 21 | Rrcbc40 | CAAGCAGAAGACGGCATACGAGATGAACACTTTGGAAACACGTTTTAACGTCRTCCCCDCCTTCCTC | Fbc1 | AATGATACGGCGACCACCGAGATCTACACACGAGACTGATTTACCGGGACTTAGGATTAGATACCCBRGTAGTC |
| 22 | Rrcbc41 | CAAGCAGAAGACGGCATACGAGATGAGCCATCTGTAAACACGTTTTAACGTCRTCCCCDCCTTCCTC | Fbc1 | AATGATACGGCGACCACCGAGATCTACACACGAGACTGATTTACCGGGACTTAGGATTAGATACCCBRGTAGTC |
| 23 | Rrcbc42 | CAAGCAGAAGACGGCATACGAGATTTGGGTACACGTAACACGTTTTAACGTCRTCCCCDCCTTCCTC | Fbc1 | AATGATACGGCGACCACCGAGATCTACACACGAGACTGATTTACCGGGACTTAGGATTAGATACCCBRGTAGTC |
| 24 | Rrcbc43 | CAAGCAGAAGACGGCATACGAGATAAGGCGCTCCTTAACACGTTTTAACGTCRTCCCCDCCTTCCTC | Fbc1 | AATGATACGGCGACCACCGAGATCTACACACGAGACTGATTTACCGGGACTTAGGATTAGATACCCBRGTAGTC |
| 25 | Rrcbc32 | CAAGCAGAAGACGGCATACGAGATGCATATGCACTGAACACGTTTTAACGTCRTCCCCDCCTTCCTC | Fbc2 | AATGATACGGCGACCACCGAGATCTACACGCTGTACGGATTTACCGGGACTTAGGATTAGATACCCBRGTAGTC |
| 26 | Rrcbc33 | CAAGCAGAAGACGGCATACGAGATCAACTCCCGTGAAACACGTTTTAACGTCRTCCCCDCCTTCCTC | Fbc2 | AATGATACGGCGACCACCGAGATCTACACGCTGTACGGATTTACCGGGACTTAGGATTAGATACCCBRGTAGTC |
| 27 | Rrcbc34 | CAAGCAGAAGACGGCATACGAGATTTGCGTTAGCAGAACACGTTTTAACGTCRTCCCCDCCTTCCTC | Fbc2 | AATGATACGGCGACCACCGAGATCTACACGCTGTACGGATTTACCGGGACTTAGGATTAGATACCCBRGTAGTC |
| 28 | Rrcbc35 | CAAGCAGAAGACGGCATACGAGATTACGAGCCCTAAAACACGTTTTAACGTCRTCCCCDCCTTCCTC | Fbc2 | AATGATACGGCGACCACCGAGATCTACACGCTGTACGGATTTACCGGGACTTAGGATTAGATACCCBRGTAGTC |
| 29 | Rrcbc36 | CAAGCAGAAGACGGCATACGAGATCACTACGCTAGAAACACGTTTTAACGTCRTCCCCDCCTTCCTC | Fbc2 | AATGATACGGCGACCACCGAGATCTACACGCTGTACGGATTTACCGGGACTTAGGATTAGATACCCBRGTAGTC |
| 30 | Rrcbc37 | CAAGCAGAAGACGGCATACGAGATTGCAGTCCTCGAAACACGTTTTAACGTCRTCCCCDCCTTCCTC | Fbc2 | AATGATACGGCGACCACCGAGATCTACACGCTGTACGGATTTACCGGGACTTAGGATTAGATACCCBRGTAGTC |
| 31 | Rrcbc38 | CAAGCAGAAGACGGCATACGAGATACCATAGCTCCGAACACGTTTTAACGTCRTCCCCDCCTTCCTC | Fbc2 | AATGATACGGCGACCACCGAGATCTACACGCTGTACGGATTTACCGGGACTTAGGATTAGATACCCBRGTAGTC |
| 32 | Rrcbc39 | CAAGCAGAAGACGGCATACGAGATTCGACATCTCTTAACACGTTTTAACGTCRTCCCCDCCTTCCTC | Fbc2 | AATGATACGGCGACCACCGAGATCTACACGCTGTACGGATTTACCGGGACTTAGGATTAGATACCCBRGTAGTC |
| 33 | Rrcbc40 | CAAGCAGAAGACGGCATACGAGATGAACACTTTGGAAACACGTTTTAACGTCRTCCCCDCCTTCCTC | Fbc2 | AATGATACGGCGACCACCGAGATCTACACGCTGTACGGATTTACCGGGACTTAGGATTAGATACCCBRGTAGTC |
| 34 | Rrcbc41 | CAAGCAGAAGACGGCATACGAGATGAGCCATCTGTAAACACGTTTTAACGTCRTCCCCDCCTTCCTC | Fbc2 | AATGATACGGCGACCACCGAGATCTACACGCTGTACGGATTTACCGGGACTTAGGATTAGATACCCBRGTAGTC |
| 35 | Rrcbc42 | CAAGCAGAAGACGGCATACGAGATTTGGGTACACGTAACACGTTTTAACGTCRTCCCCDCCTTCCTC | Fbc2 | AATGATACGGCGACCACCGAGATCTACACGCTGTACGGATTTACCGGGACTTAGGATTAGATACCCBRGTAGTC |
| 36 | Rrcbc43 | CAAGCAGAAGACGGCATACGAGATAAGGCGCTCCTTAACACGTTTTAACGTCRTCCCCDCCTTCCTC | Fbc2 | AATGATACGGCGACCACCGAGATCTACACGCTGTACGGATTTACCGGGACTTAGGATTAGATACCCBRGTAGTC |
| 37 | Rrcbc32 | CAAGCAGAAGACGGCATACGAGATGCATATGCACTGAACACGTTTTAACGTCRTCCCCDCCTTCCTC | Fbc3 | AATGATACGGCGACCACCGAGATCTACACATCACCAGGTGTTACCGGGACTTAGGATTAGATACCCBRGTAGTC |
| 38 | Rrcbc33 | CAAGCAGAAGACGGCATACGAGATCAACTCCCGTGAAACACGTTTTAACGTCRTCCCCDCCTTCCTC | Fbc3 | AATGATACGGCGACCACCGAGATCTACACATCACCAGGTGTTACCGGGACTTAGGATTAGATACCCBRGTAGTC |
| 39 | Rrcbc34 | CAAGCAGAAGACGGCATACGAGATTTGCGTTAGCAGAACACGTTTTAACGTCRTCCCCDCCTTCCTC | Fbc3 | AATGATACGGCGACCACCGAGATCTACACATCACCAGGTGTTACCGGGACTTAGGATTAGATACCCBRGTAGTC |
| 40 | Rrcbc35 | CAAGCAGAAGACGGCATACGAGATTACGAGCCCTAAAACACGTTTTAACGTCRTCCCCDCCTTCCTC | Fbc3 | AATGATACGGCGACCACCGAGATCTACACATCACCAGGTGTTACCGGGACTTAGGATTAGATACCCBRGTAGTC |
| 41 | Rrcbc36 | CAAGCAGAAGACGGCATACGAGATCACTACGCTAGAAACACGTTTTAACGTCRTCCCCDCCTTCCTC | Fbc3 | AATGATACGGCGACCACCGAGATCTACACATCACCAGGTGTTACCGGGACTTAGGATTAGATACCCBRGTAGTC |
| 42 | Rrcbc37 | CAAGCAGAAGACGGCATACGAGATTGCAGTCCTCGAAACACGTTTTAACGTCRTCCCCDCCTTCCTC | Fbc3 | AATGATACGGCGACCACCGAGATCTACACATCACCAGGTGTTACCGGGACTTAGGATTAGATACCCBRGTAGTC |
| 43 | Rrcbc38 | CAAGCAGAAGACGGCATACGAGATACCATAGCTCCGAACACGTTTTAACGTCRTCCCCDCCTTCCTC | Fbc3 | AATGATACGGCGACCACCGAGATCTACACATCACCAGGTGTTACCGGGACTTAGGATTAGATACCCBRGTAGTC |
| 44 | Rrcbc39 | CAAGCAGAAGACGGCATACGAGATTCGACATCTCTTAACACGTTTTAACGTCRTCCCCDCCTTCCTC | Fbc3 | AATGATACGGCGACCACCGAGATCTACACATCACCAGGTGTTACCGGGACTTAGGATTAGATACCCBRGTAGTC |
| 45 | Rrcbc40 | CAAGCAGAAGACGGCATACGAGATGAACACTTTGGAAACACGTTTTAACGTCRTCCCCDCCTTCCTC | Fbc3 | AATGATACGGCGACCACCGAGATCTACACATCACCAGGTGTTACCGGGACTTAGGATTAGATACCCBRGTAGTC |
| 46 | Rrcbc41 | CAAGCAGAAGACGGCATACGAGATGAGCCATCTGTAAACACGTTTTAACGTCRTCCCCDCCTTCCTC | Fbc3 | AATGATACGGCGACCACCGAGATCTACACATCACCAGGTGTTACCGGGACTTAGGATTAGATACCCBRGTAGTC |
| 47 | Rrcbc42 | CAAGCAGAAGACGGCATACGAGATTTGGGTACACGTAACACGTTTTAACGTCRTCCCCDCCTTCCTC | Fbc3 | AATGATACGGCGACCACCGAGATCTACACATCACCAGGTGTTACCGGGACTTAGGATTAGATACCCBRGTAGTC |
| 48 | Rrcbc43 | CAAGCAGAAGACGGCATACGAGATAAGGCGCTCCTTAACACGTTTTAACGTCRTCCCCDCCTTCCTC | Fbc3 | AATGATACGGCGACCACCGAGATCTACACATCACCAGGTGTTACCGGGACTTAGGATTAGATACCCBRGTAGTC |
| 49 | Rrcbc32 | CAAGCAGAAGACGGCATACGAGATGCATATGCACTGAACACGTTTTAACGTCRTCCCCDCCTTCCTC | Fbc4 | AATGATACGGCGACCACCGAGATCTACACTGGTCAACGATATACCGGGACTTAGGATTAGATACCCBRGTAGTC |
| 50 | Rrcbc33 | CAAGCAGAAGACGGCATACGAGATCAACTCCCGTGAAACACGTTTTAACGTCRTCCCCDCCTTCCTC | Fbc4 | AATGATACGGCGACCACCGAGATCTACACTGGTCAACGATATACCGGGACTTAGGATTAGATACCCBRGTAGTC |
| 51 | Rrcbc34 | CAAGCAGAAGACGGCATACGAGATTTGCGTTAGCAGAACACGTTTTAACGTCRTCCCCDCCTTCCTC | Fbc4 | AATGATACGGCGACCACCGAGATCTACACTGGTCAACGATATACCGGGACTTAGGATTAGATACCCBRGTAGTC |
| 52 | Rrcbc35 | CAAGCAGAAGACGGCATACGAGATTACGAGCCCTAAAACACGTTTTAACGTCRTCCCCDCCTTCCTC | Fbc4 | AATGATACGGCGACCACCGAGATCTACACTGGTCAACGATATACCGGGACTTAGGATTAGATACCCBRGTAGTC |
| 53 | Rrcbc36 | CAAGCAGAAGACGGCATACGAGATCACTACGCTAGAAACACGTTTTAACGTCRTCCCCDCCTTCCTC | Fbc4 | AATGATACGGCGACCACCGAGATCTACACTGGTCAACGATATACCGGGACTTAGGATTAGATACCCBRGTAGTC |
| 54 | Rrcbc37 | CAAGCAGAAGACGGCATACGAGATTGCAGTCCTCGAAACACGTTTTAACGTCRTCCCCDCCTTCCTC | Fbc4 | AATGATACGGCGACCACCGAGATCTACACTGGTCAACGATATACCGGGACTTAGGATTAGATACCCBRGTAGTC |
| 55 | Rrcbc38 | CAAGCAGAAGACGGCATACGAGATACCATAGCTCCGAACACGTTTTAACGTCRTCCCCDCCTTCCTC | Fbc4 | AATGATACGGCGACCACCGAGATCTACACTGGTCAACGATATACCGGGACTTAGGATTAGATACCCBRGTAGTC |
| 56 | Rrcbc39 | CAAGCAGAAGACGGCATACGAGATTCGACATCTCTTAACACGTTTTAACGTCRTCCCCDCCTTCCTC | Fbc4 | AATGATACGGCGACCACCGAGATCTACACTGGTCAACGATATACCGGGACTTAGGATTAGATACCCBRGTAGTC |
| 57 | Rrcbc40 | CAAGCAGAAGACGGCATACGAGATGAACACTTTGGAAACACGTTTTAACGTCRTCCCCDCCTTCCTC | Fbc4 | AATGATACGGCGACCACCGAGATCTACACTGGTCAACGATATACCGGGACTTAGGATTAGATACCCBRGTAGTC |
| 58 | Rrcbc41 | CAAGCAGAAGACGGCATACGAGATGAGCCATCTGTAAACACGTTTTAACGTCRTCCCCDCCTTCCTC | Fbc4 | AATGATACGGCGACCACCGAGATCTACACTGGTCAACGATATACCGGGACTTAGGATTAGATACCCBRGTAGTC |
| 59 | Rrcbc42 | CAAGCAGAAGACGGCATACGAGATTTGGGTACACGTAACACGTTTTAACGTCRTCCCCDCCTTCCTC | Fbc4 | AATGATACGGCGACCACCGAGATCTACACTGGTCAACGATATACCGGGACTTAGGATTAGATACCCBRGTAGTC |
| 60 | Rrcbc43 | CAAGCAGAAGACGGCATACGAGATAAGGCGCTCCTTAACACGTTTTAACGTCRTCCCCDCCTTCCTC | Fbc4 | AATGATACGGCGACCACCGAGATCTACACTGGTCAACGATATACCGGGACTTAGGATTAGATACCCBRGTAGTC |
| 61 | Rrcbc32 | CAAGCAGAAGACGGCATACGAGATGCATATGCACTGAACACGTTTTAACGTCRTCCCCDCCTTCCTC | Fbc5 | AATGATACGGCGACCACCGAGATCTACACATCGCACAGTAATACCGGGACTTAGGATTAGATACCCBRGTAGTC |
| 62 | Rrcbc33 | CAAGCAGAAGACGGCATACGAGATCAACTCCCGTGAAACACGTTTTAACGTCRTCCCCDCCTTCCTC | Fbc5 | AATGATACGGCGACCACCGAGATCTACACATCGCACAGTAATACCGGGACTTAGGATTAGATACCCBRGTAGTC |
| 63 | Rrcbc34 | CAAGCAGAAGACGGCATACGAGATTTGCGTTAGCAGAACACGTTTTAACGTCRTCCCCDCCTTCCTC | Fbc5 | AATGATACGGCGACCACCGAGATCTACACATCGCACAGTAATACCGGGACTTAGGATTAGATACCCBRGTAGTC |
| 64 | Rrcbc35 | CAAGCAGAAGACGGCATACGAGATTACGAGCCCTAAAACACGTTTTAACGTCRTCCCCDCCTTCCTC | Fbc5 | AATGATACGGCGACCACCGAGATCTACACATCGCACAGTAATACCGGGACTTAGGATTAGATACCCBRGTAGTC |
| 65 | Rrcbc36 | CAAGCAGAAGACGGCATACGAGATCACTACGCTAGAAACACGTTTTAACGTCRTCCCCDCCTTCCTC | Fbc5 | AATGATACGGCGACCACCGAGATCTACACATCGCACAGTAATACCGGGACTTAGGATTAGATACCCBRGTAGTC |
| 66 | Rrcbc37 | CAAGCAGAAGACGGCATACGAGATTGCAGTCCTCGAAACACGTTTTAACGTCRTCCCCDCCTTCCTC | Fbc5 | AATGATACGGCGACCACCGAGATCTACACATCGCACAGTAATACCGGGACTTAGGATTAGATACCCBRGTAGTC |
| 67 | Rrcbc38 | CAAGCAGAAGACGGCATACGAGATACCATAGCTCCGAACACGTTTTAACGTCRTCCCCDCCTTCCTC | Fbc5 | AATGATACGGCGACCACCGAGATCTACACATCGCACAGTAATACCGGGACTTAGGATTAGATACCCBRGTAGTC |
| 68 | Rrcbc39 | CAAGCAGAAGACGGCATACGAGATTCGACATCTCTTAACACGTTTTAACGTCRTCCCCDCCTTCCTC | Fbc5 | AATGATACGGCGACCACCGAGATCTACACATCGCACAGTAATACCGGGACTTAGGATTAGATACCCBRGTAGTC |
| 69 | Rrcbc40 | CAAGCAGAAGACGGCATACGAGATGAACACTTTGGAAACACGTTTTAACGTCRTCCCCDCCTTCCTC | Fbc5 | AATGATACGGCGACCACCGAGATCTACACATCGCACAGTAATACCGGGACTTAGGATTAGATACCCBRGTAGTC |
| 70 | Rrcbc41 | CAAGCAGAAGACGGCATACGAGATGAGCCATCTGTAAACACGTTTTAACGTCRTCCCCDCCTTCCTC | Fbc5 | AATGATACGGCGACCACCGAGATCTACACATCGCACAGTAATACCGGGACTTAGGATTAGATACCCBRGTAGTC |
| 71 | Rrcbc42 | CAAGCAGAAGACGGCATACGAGATTTGGGTACACGTAACACGTTTTAACGTCRTCCCCDCCTTCCTC | Fbc5 | AATGATACGGCGACCACCGAGATCTACACATCGCACAGTAATACCGGGACTTAGGATTAGATACCCBRGTAGTC |
| 72 | Rrcbc43 | CAAGCAGAAGACGGCATACGAGATAAGGCGCTCCTTAACACGTTTTAACGTCRTCCCCDCCTTCCTC | Fbc5 | AATGATACGGCGACCACCGAGATCTACACATCGCACAGTAATACCGGGACTTAGGATTAGATACCCBRGTAGTC |
| 73 | Rrcbc32 | CAAGCAGAAGACGGCATACGAGATGCATATGCACTGAACACGTTTTAACGTCRTCCCCDCCTTCCTC | Fbc6 | AATGATACGGCGACCACCGAGATCTACACGTCGTGTAGCCTTACCGGGACTTAGGATTAGATACCCBRGTAGTC |
| 74 | Rrcbc33 | CAAGCAGAAGACGGCATACGAGATCAACTCCCGTGAAACACGTTTTAACGTCRTCCCCDCCTTCCTC | Fbc6 | AATGATACGGCGACCACCGAGATCTACACGTCGTGTAGCCTTACCGGGACTTAGGATTAGATACCCBRGTAGTC |
| 75 | Rrcbc34 | CAAGCAGAAGACGGCATACGAGATTTGCGTTAGCAGAACACGTTTTAACGTCRTCCCCDCCTTCCTC | Fbc6 | AATGATACGGCGACCACCGAGATCTACACGTCGTGTAGCCTTACCGGGACTTAGGATTAGATACCCBRGTAGTC |
| 76 | Rrcbc35 | CAAGCAGAAGACGGCATACGAGATTACGAGCCCTAAAACACGTTTTAACGTCRTCCCCDCCTTCCTC | Fbc6 | AATGATACGGCGACCACCGAGATCTACACGTCGTGTAGCCTTACCGGGACTTAGGATTAGATACCCBRGTAGTC |
| 77 | Rrcbc36 | CAAGCAGAAGACGGCATACGAGATCACTACGCTAGAAACACGTTTTAACGTCRTCCCCDCCTTCCTC | Fbc6 | AATGATACGGCGACCACCGAGATCTACACGTCGTGTAGCCTTACCGGGACTTAGGATTAGATACCCBRGTAGTC |
| 78 | Rrcbc37 | CAAGCAGAAGACGGCATACGAGATTGCAGTCCTCGAAACACGTTTTAACGTCRTCCCCDCCTTCCTC | Fbc6 | AATGATACGGCGACCACCGAGATCTACACGTCGTGTAGCCTTACCGGGACTTAGGATTAGATACCCBRGTAGTC |
| 79 | Rrcbc38 | CAAGCAGAAGACGGCATACGAGATACCATAGCTCCGAACACGTTTTAACGTCRTCCCCDCCTTCCTC | Fbc6 | AATGATACGGCGACCACCGAGATCTACACGTCGTGTAGCCTTACCGGGACTTAGGATTAGATACCCBRGTAGTC |
| 80 | Rrcbc39 | CAAGCAGAAGACGGCATACGAGATTCGACATCTCTTAACACGTTTTAACGTCRTCCCCDCCTTCCTC | Fbc6 | AATGATACGGCGACCACCGAGATCTACACGTCGTGTAGCCTTACCGGGACTTAGGATTAGATACCCBRGTAGTC |
| 81 | Rrcbc40 | CAAGCAGAAGACGGCATACGAGATGAACACTTTGGAAACACGTTTTAACGTCRTCCCCDCCTTCCTC | Fbc6 | AATGATACGGCGACCACCGAGATCTACACGTCGTGTAGCCTTACCGGGACTTAGGATTAGATACCCBRGTAGTC |
| 82 | Rrcbc41 | CAAGCAGAAGACGGCATACGAGATGAGCCATCTGTAAACACGTTTTAACGTCRTCCCCDCCTTCCTC | Fbc6 | AATGATACGGCGACCACCGAGATCTACACGTCGTGTAGCCTTACCGGGACTTAGGATTAGATACCCBRGTAGTC |
| 83 | Rrcbc42 | CAAGCAGAAGACGGCATACGAGATTTGGGTACACGTAACACGTTTTAACGTCRTCCCCDCCTTCCTC | Fbc6 | AATGATACGGCGACCACCGAGATCTACACGTCGTGTAGCCTTACCGGGACTTAGGATTAGATACCCBRGTAGTC |
| 84 | Rrcbc43 | CAAGCAGAAGACGGCATACGAGATAAGGCGCTCCTTAACACGTTTTAACGTCRTCCCCDCCTTCCTC | Fbc6 | AATGATACGGCGACCACCGAGATCTACACGTCGTGTAGCCTTACCGGGACTTAGGATTAGATACCCBRGTAGTC |
| 85 | Rrcbc32 | CAAGCAGAAGACGGCATACGAGATGCATATGCACTGAACACGTTTTAACGTCRTCCCCDCCTTCCTC | Fbc7 | AATGATACGGCGACCACCGAGATCTACACAGCGGAGGTTAGTACCGGGACTTAGGATTAGATACCCBRGTAGTC |
| 86 | Rrcbc33 | CAAGCAGAAGACGGCATACGAGATCAACTCCCGTGAAACACGTTTTAACGTCRTCCCCDCCTTCCTC | Fbc7 | AATGATACGGCGACCACCGAGATCTACACAGCGGAGGTTAGTACCGGGACTTAGGATTAGATACCCBRGTAGTC |
| 87 | Rrcbc34 | CAAGCAGAAGACGGCATACGAGATTTGCGTTAGCAGAACACGTTTTAACGTCRTCCCCDCCTTCCTC | Fbc7 | AATGATACGGCGACCACCGAGATCTACACAGCGGAGGTTAGTACCGGGACTTAGGATTAGATACCCBRGTAGTC |
| 88 | Rrcbc35 | CAAGCAGAAGACGGCATACGAGATTACGAGCCCTAAAACACGTTTTAACGTCRTCCCCDCCTTCCTC | Fbc7 | AATGATACGGCGACCACCGAGATCTACACAGCGGAGGTTAGTACCGGGACTTAGGATTAGATACCCBRGTAGTC |
| 89 | Rrcbc36 | CAAGCAGAAGACGGCATACGAGATCACTACGCTAGAAACACGTTTTAACGTCRTCCCCDCCTTCCTC | Fbc7 | AATGATACGGCGACCACCGAGATCTACACAGCGGAGGTTAGTACCGGGACTTAGGATTAGATACCCBRGTAGTC |
| 90 | Rrcbc37 | CAAGCAGAAGACGGCATACGAGATTGCAGTCCTCGAAACACGTTTTAACGTCRTCCCCDCCTTCCTC | Fbc7 | AATGATACGGCGACCACCGAGATCTACACAGCGGAGGTTAGTACCGGGACTTAGGATTAGATACCCBRGTAGTC |
| 91 | Rrcbc38 | CAAGCAGAAGACGGCATACGAGATACCATAGCTCCGAACACGTTTTAACGTCRTCCCCDCCTTCCTC | Fbc7 | AATGATACGGCGACCACCGAGATCTACACAGCGGAGGTTAGTACCGGGACTTAGGATTAGATACCCBRGTAGTC |
| 92 | Rrcbc39 | CAAGCAGAAGACGGCATACGAGATTCGACATCTCTTAACACGTTTTAACGTCRTCCCCDCCTTCCTC | Fbc7 | AATGATACGGCGACCACCGAGATCTACACAGCGGAGGTTAGTACCGGGACTTAGGATTAGATACCCBRGTAGTC |
| 93 | Rrcbc40 | CAAGCAGAAGACGGCATACGAGATGAACACTTTGGAAACACGTTTTAACGTCRTCCCCDCCTTCCTC | Fbc7 | AATGATACGGCGACCACCGAGATCTACACAGCGGAGGTTAGTACCGGGACTTAGGATTAGATACCCBRGTAGTC |
| 94 | Rrcbc41 | CAAGCAGAAGACGGCATACGAGATGAGCCATCTGTAAACACGTTTTAACGTCRTCCCCDCCTTCCTC | Fbc7 | AATGATACGGCGACCACCGAGATCTACACAGCGGAGGTTAGTACCGGGACTTAGGATTAGATACCCBRGTAGTC |
| 95 | Rrcbc42 | CAAGCAGAAGACGGCATACGAGATTTGGGTACACGTAACACGTTTTAACGTCRTCCCCDCCTTCCTC | Fbc7 | AATGATACGGCGACCACCGAGATCTACACAGCGGAGGTTAGTACCGGGACTTAGGATTAGATACCCBRGTAGTC |
| 96 | Rrcbc43 | CAAGCAGAAGACGGCATACGAGATAAGGCGCTCCTTAACACGTTTTAACGTCRTCCCCDCCTTCCTC | Fbc7 | AATGATACGGCGACCACCGAGATCTACACAGCGGAGGTTAGTACCGGGACTTAGGATTAGATACCCBRGTAGTC |
| 97 | Rrcbc44 | CAAGCAGAAGACGGCATACGAGATTAATACGGATCGAACACGTTTTAACGTCRTCCCCDCCTTCCTC | Fbc0 | AATGATACGGCGACCACCGAGATCTACACTCCCTTGTCTCCTACCGGGACTTAGGATTAGATACCCBRGTAGTC |
| 98 | Rrcbc45 | CAAGCAGAAGACGGCATACGAGATTCGGAATTAGACAACACGTTTTAACGTCRTCCCCDCCTTCCTC | Fbc0 | AATGATACGGCGACCACCGAGATCTACACTCCCTTGTCTCCTACCGGGACTTAGGATTAGATACCCBRGTAGTC |
| 99 | Rrcbc46 | CAAGCAGAAGACGGCATACGAGATTGTGAATTCGGAAACACGTTTTAACGTCRTCCCCDCCTTCCTC | Fbc0 | AATGATACGGCGACCACCGAGATCTACACTCCCTTGTCTCCTACCGGGACTTAGGATTAGATACCCBRGTAGTC |
| 100 | Rrcbc47 | CAAGCAGAAGACGGCATACGAGATCATTCGTGGCGTAACACGTTTTAACGTCRTCCCCDCCTTCCTC | Fbc0 | AATGATACGGCGACCACCGAGATCTACACTCCCTTGTCTCCTACCGGGACTTAGGATTAGATACCCBRGTAGTC |
| 101 | Rrcbc48 | CAAGCAGAAGACGGCATACGAGATTACTACGTGGCCAACACGTTTTAACGTCRTCCCCDCCTTCCTC | Fbc0 | AATGATACGGCGACCACCGAGATCTACACTCCCTTGTCTCCTACCGGGACTTAGGATTAGATACCCBRGTAGTC |
| 102 | Rrcbc49 | CAAGCAGAAGACGGCATACGAGATGGCCAGTTCCTAAACACGTTTTAACGTCRTCCCCDCCTTCCTC | Fbc0 | AATGATACGGCGACCACCGAGATCTACACTCCCTTGTCTCCTACCGGGACTTAGGATTAGATACCCBRGTAGTC |
| 103 | Rrcbc50 | CAAGCAGAAGACGGCATACGAGATGATGTTCGCTAGAACACGTTTTAACGTCRTCCCCDCCTTCCTC | Fbc0 | AATGATACGGCGACCACCGAGATCTACACTCCCTTGTCTCCTACCGGGACTTAGGATTAGATACCCBRGTAGTC |
| 104 | Rrcbc51 | CAAGCAGAAGACGGCATACGAGATCTATCTCCTGTCAACACGTTTTAACGTCRTCCCCDCCTTCCTC | Fbc0 | AATGATACGGCGACCACCGAGATCTACACTCCCTTGTCTCCTACCGGGACTTAGGATTAGATACCCBRGTAGTC |
| 105 | Rrcbc52 | CAAGCAGAAGACGGCATACGAGATACTCACAGGAATAACACGTTTTAACGTCRTCCCCDCCTTCCTC | Fbc0 | AATGATACGGCGACCACCGAGATCTACACTCCCTTGTCTCCTACCGGGACTTAGGATTAGATACCCBRGTAGTC |
| 106 | Rrcbc53 | CAAGCAGAAGACGGCATACGAGATATGATGAGCCTCAACACGTTTTAACGTCRTCCCCDCCTTCCTC | Fbc0 | AATGATACGGCGACCACCGAGATCTACACTCCCTTGTCTCCTACCGGGACTTAGGATTAGATACCCBRGTAGTC |
| 107 | Rrcbc54 | CAAGCAGAAGACGGCATACGAGATGTCGACAGAGGAAACACGTTTTAACGTCRTCCCCDCCTTCCTC | Fbc0 | AATGATACGGCGACCACCGAGATCTACACTCCCTTGTCTCCTACCGGGACTTAGGATTAGATACCCBRGTAGTC |
| 108 | Rrcbc55 | CAAGCAGAAGACGGCATACGAGATTGTCGCAAATAGAACACGTTTTAACGTCRTCCCCDCCTTCCTC | Fbc0 | AATGATACGGCGACCACCGAGATCTACACTCCCTTGTCTCCTACCGGGACTTAGGATTAGATACCCBRGTAGTC |
| 109 | Rrcbc44 | CAAGCAGAAGACGGCATACGAGATTAATACGGATCGAACACGTTTTAACGTCRTCCCCDCCTTCCTC | Fbc1 | AATGATACGGCGACCACCGAGATCTACACACGAGACTGATTTACCGGGACTTAGGATTAGATACCCBRGTAGTC |
| 110 | Rrcbc45 | CAAGCAGAAGACGGCATACGAGATTCGGAATTAGACAACACGTTTTAACGTCRTCCCCDCCTTCCTC | Fbc1 | AATGATACGGCGACCACCGAGATCTACACACGAGACTGATTTACCGGGACTTAGGATTAGATACCCBRGTAGTC |
| 111 | Rrcbc46 | CAAGCAGAAGACGGCATACGAGATTGTGAATTCGGAAACACGTTTTAACGTCRTCCCCDCCTTCCTC | Fbc1 | AATGATACGGCGACCACCGAGATCTACACACGAGACTGATTTACCGGGACTTAGGATTAGATACCCBRGTAGTC |
| 112 | Rrcbc47 | CAAGCAGAAGACGGCATACGAGATCATTCGTGGCGTAACACGTTTTAACGTCRTCCCCDCCTTCCTC | Fbc1 | AATGATACGGCGACCACCGAGATCTACACACGAGACTGATTTACCGGGACTTAGGATTAGATACCCBRGTAGTC |
| 113 | Rrcbc48 | CAAGCAGAAGACGGCATACGAGATTACTACGTGGCCAACACGTTTTAACGTCRTCCCCDCCTTCCTC | Fbc1 | AATGATACGGCGACCACCGAGATCTACACACGAGACTGATTTACCGGGACTTAGGATTAGATACCCBRGTAGTC |
| 114 | Rrcbc49 | CAAGCAGAAGACGGCATACGAGATGGCCAGTTCCTAAACACGTTTTAACGTCRTCCCCDCCTTCCTC | Fbc1 | AATGATACGGCGACCACCGAGATCTACACACGAGACTGATTTACCGGGACTTAGGATTAGATACCCBRGTAGTC |
| 115 | Rrcbc50 | CAAGCAGAAGACGGCATACGAGATGATGTTCGCTAGAACACGTTTTAACGTCRTCCCCDCCTTCCTC | Fbc1 | AATGATACGGCGACCACCGAGATCTACACACGAGACTGATTTACCGGGACTTAGGATTAGATACCCBRGTAGTC |
| 116 | Rrcbc51 | CAAGCAGAAGACGGCATACGAGATCTATCTCCTGTCAACACGTTTTAACGTCRTCCCCDCCTTCCTC | Fbc1 | AATGATACGGCGACCACCGAGATCTACACACGAGACTGATTTACCGGGACTTAGGATTAGATACCCBRGTAGTC |
| 117 | Rrcbc52 | CAAGCAGAAGACGGCATACGAGATACTCACAGGAATAACACGTTTTAACGTCRTCCCCDCCTTCCTC | Fbc1 | AATGATACGGCGACCACCGAGATCTACACACGAGACTGATTTACCGGGACTTAGGATTAGATACCCBRGTAGTC |
| 118 | Rrcbc53 | CAAGCAGAAGACGGCATACGAGATATGATGAGCCTCAACACGTTTTAACGTCRTCCCCDCCTTCCTC | Fbc1 | AATGATACGGCGACCACCGAGATCTACACACGAGACTGATTTACCGGGACTTAGGATTAGATACCCBRGTAGTC |
| 119 | Rrcbc54 | CAAGCAGAAGACGGCATACGAGATGTCGACAGAGGAAACACGTTTTAACGTCRTCCCCDCCTTCCTC | Fbc1 | AATGATACGGCGACCACCGAGATCTACACACGAGACTGATTTACCGGGACTTAGGATTAGATACCCBRGTAGTC |
| 120 | Rrcbc55 | CAAGCAGAAGACGGCATACGAGATTGTCGCAAATAGAACACGTTTTAACGTCRTCCCCDCCTTCCTC | Fbc1 | AATGATACGGCGACCACCGAGATCTACACACGAGACTGATTTACCGGGACTTAGGATTAGATACCCBRGTAGTC |
| 121 | Rrcbc44 | CAAGCAGAAGACGGCATACGAGATTAATACGGATCGAACACGTTTTAACGTCRTCCCCDCCTTCCTC | Fbc2 | AATGATACGGCGACCACCGAGATCTACACGCTGTACGGATTTACCGGGACTTAGGATTAGATACCCBRGTAGTC |
| 122 | Rrcbc45 | CAAGCAGAAGACGGCATACGAGATTCGGAATTAGACAACACGTTTTAACGTCRTCCCCDCCTTCCTC | Fbc2 | AATGATACGGCGACCACCGAGATCTACACGCTGTACGGATTTACCGGGACTTAGGATTAGATACCCBRGTAGTC |
| 123 | Rrcbc46 | CAAGCAGAAGACGGCATACGAGATTGTGAATTCGGAAACACGTTTTAACGTCRTCCCCDCCTTCCTC | Fbc2 | AATGATACGGCGACCACCGAGATCTACACGCTGTACGGATTTACCGGGACTTAGGATTAGATACCCBRGTAGTC |
| 124 | Rrcbc47 | CAAGCAGAAGACGGCATACGAGATCATTCGTGGCGTAACACGTTTTAACGTCRTCCCCDCCTTCCTC | Fbc2 | AATGATACGGCGACCACCGAGATCTACACGCTGTACGGATTTACCGGGACTTAGGATTAGATACCCBRGTAGTC |
| 125 | Rrcbc48 | CAAGCAGAAGACGGCATACGAGATTACTACGTGGCCAACACGTTTTAACGTCRTCCCCDCCTTCCTC | Fbc2 | AATGATACGGCGACCACCGAGATCTACACGCTGTACGGATTTACCGGGACTTAGGATTAGATACCCBRGTAGTC |
| 126 | Rrcbc49 | CAAGCAGAAGACGGCATACGAGATGGCCAGTTCCTAAACACGTTTTAACGTCRTCCCCDCCTTCCTC | Fbc2 | AATGATACGGCGACCACCGAGATCTACACGCTGTACGGATTTACCGGGACTTAGGATTAGATACCCBRGTAGTC |
| 127 | Rrcbc50 | CAAGCAGAAGACGGCATACGAGATGATGTTCGCTAGAACACGTTTTAACGTCRTCCCCDCCTTCCTC | Fbc2 | AATGATACGGCGACCACCGAGATCTACACGCTGTACGGATTTACCGGGACTTAGGATTAGATACCCBRGTAGTC |
| 128 | Rrcbc51 | CAAGCAGAAGACGGCATACGAGATCTATCTCCTGTCAACACGTTTTAACGTCRTCCCCDCCTTCCTC | Fbc2 | AATGATACGGCGACCACCGAGATCTACACGCTGTACGGATTTACCGGGACTTAGGATTAGATACCCBRGTAGTC |
| 129 | Rrcbc52 | CAAGCAGAAGACGGCATACGAGATACTCACAGGAATAACACGTTTTAACGTCRTCCCCDCCTTCCTC | Fbc2 | AATGATACGGCGACCACCGAGATCTACACGCTGTACGGATTTACCGGGACTTAGGATTAGATACCCBRGTAGTC |
| 130 | Rrcbc53 | CAAGCAGAAGACGGCATACGAGATATGATGAGCCTCAACACGTTTTAACGTCRTCCCCDCCTTCCTC | Fbc2 | AATGATACGGCGACCACCGAGATCTACACGCTGTACGGATTTACCGGGACTTAGGATTAGATACCCBRGTAGTC |
| 131 | Rrcbc54 | CAAGCAGAAGACGGCATACGAGATGTCGACAGAGGAAACACGTTTTAACGTCRTCCCCDCCTTCCTC | Fbc2 | AATGATACGGCGACCACCGAGATCTACACGCTGTACGGATTTACCGGGACTTAGGATTAGATACCCBRGTAGTC |
| 132 | Rrcbc55 | CAAGCAGAAGACGGCATACGAGATTGTCGCAAATAGAACACGTTTTAACGTCRTCCCCDCCTTCCTC | Fbc2 | AATGATACGGCGACCACCGAGATCTACACGCTGTACGGATTTACCGGGACTTAGGATTAGATACCCBRGTAGTC |
| 133 | Rrcbc44 | CAAGCAGAAGACGGCATACGAGATTAATACGGATCGAACACGTTTTAACGTCRTCCCCDCCTTCCTC | Fbc3 | AATGATACGGCGACCACCGAGATCTACACATCACCAGGTGTTACCGGGACTTAGGATTAGATACCCBRGTAGTC |
| 134 | Rrcbc45 | CAAGCAGAAGACGGCATACGAGATTCGGAATTAGACAACACGTTTTAACGTCRTCCCCDCCTTCCTC | Fbc3 | AATGATACGGCGACCACCGAGATCTACACATCACCAGGTGTTACCGGGACTTAGGATTAGATACCCBRGTAGTC |
| 135 | Rrcbc46 | CAAGCAGAAGACGGCATACGAGATTGTGAATTCGGAAACACGTTTTAACGTCRTCCCCDCCTTCCTC | Fbc3 | AATGATACGGCGACCACCGAGATCTACACATCACCAGGTGTTACCGGGACTTAGGATTAGATACCCBRGTAGTC |
| 136 | Rrcbc47 | CAAGCAGAAGACGGCATACGAGATCATTCGTGGCGTAACACGTTTTAACGTCRTCCCCDCCTTCCTC | Fbc3 | AATGATACGGCGACCACCGAGATCTACACATCACCAGGTGTTACCGGGACTTAGGATTAGATACCCBRGTAGTC |
| 137 | Rrcbc48 | CAAGCAGAAGACGGCATACGAGATTACTACGTGGCCAACACGTTTTAACGTCRTCCCCDCCTTCCTC | Fbc3 | AATGATACGGCGACCACCGAGATCTACACATCACCAGGTGTTACCGGGACTTAGGATTAGATACCCBRGTAGTC |
| 138 | Rrcbc49 | CAAGCAGAAGACGGCATACGAGATGGCCAGTTCCTAAACACGTTTTAACGTCRTCCCCDCCTTCCTC | Fbc3 | AATGATACGGCGACCACCGAGATCTACACATCACCAGGTGTTACCGGGACTTAGGATTAGATACCCBRGTAGTC |
| 139 | Rrcbc50 | CAAGCAGAAGACGGCATACGAGATGATGTTCGCTAGAACACGTTTTAACGTCRTCCCCDCCTTCCTC | Fbc3 | AATGATACGGCGACCACCGAGATCTACACATCACCAGGTGTTACCGGGACTTAGGATTAGATACCCBRGTAGTC |
| 140 | Rrcbc51 | CAAGCAGAAGACGGCATACGAGATCTATCTCCTGTCAACACGTTTTAACGTCRTCCCCDCCTTCCTC | Fbc3 | AATGATACGGCGACCACCGAGATCTACACATCACCAGGTGTTACCGGGACTTAGGATTAGATACCCBRGTAGTC |
| 141 | Rrcbc52 | CAAGCAGAAGACGGCATACGAGATACTCACAGGAATAACACGTTTTAACGTCRTCCCCDCCTTCCTC | Fbc3 | AATGATACGGCGACCACCGAGATCTACACATCACCAGGTGTTACCGGGACTTAGGATTAGATACCCBRGTAGTC |
| 142 | Rrcbc53 | CAAGCAGAAGACGGCATACGAGATATGATGAGCCTCAACACGTTTTAACGTCRTCCCCDCCTTCCTC | Fbc3 | AATGATACGGCGACCACCGAGATCTACACATCACCAGGTGTTACCGGGACTTAGGATTAGATACCCBRGTAGTC |
| 143 | Rrcbc54 | CAAGCAGAAGACGGCATACGAGATGTCGACAGAGGAAACACGTTTTAACGTCRTCCCCDCCTTCCTC | Fbc3 | AATGATACGGCGACCACCGAGATCTACACATCACCAGGTGTTACCGGGACTTAGGATTAGATACCCBRGTAGTC |
| 144 | Rrcbc55 | CAAGCAGAAGACGGCATACGAGATTGTCGCAAATAGAACACGTTTTAACGTCRTCCCCDCCTTCCTC | Fbc3 | AATGATACGGCGACCACCGAGATCTACACATCACCAGGTGTTACCGGGACTTAGGATTAGATACCCBRGTAGTC |
| 145 | Rrcbc44 | CAAGCAGAAGACGGCATACGAGATTAATACGGATCGAACACGTTTTAACGTCRTCCCCDCCTTCCTC | Fbc4 | AATGATACGGCGACCACCGAGATCTACACTGGTCAACGATATACCGGGACTTAGGATTAGATACCCBRGTAGTC |
| 146 | Rrcbc45 | CAAGCAGAAGACGGCATACGAGATTCGGAATTAGACAACACGTTTTAACGTCRTCCCCDCCTTCCTC | Fbc4 | AATGATACGGCGACCACCGAGATCTACACTGGTCAACGATATACCGGGACTTAGGATTAGATACCCBRGTAGTC |
| 147 | Rrcbc46 | CAAGCAGAAGACGGCATACGAGATTGTGAATTCGGAAACACGTTTTAACGTCRTCCCCDCCTTCCTC | Fbc4 | AATGATACGGCGACCACCGAGATCTACACTGGTCAACGATATACCGGGACTTAGGATTAGATACCCBRGTAGTC |
| 148 | Rrcbc47 | CAAGCAGAAGACGGCATACGAGATCATTCGTGGCGTAACACGTTTTAACGTCRTCCCCDCCTTCCTC | Fbc4 | AATGATACGGCGACCACCGAGATCTACACTGGTCAACGATATACCGGGACTTAGGATTAGATACCCBRGTAGTC |
| 149 | Rrcbc48 | CAAGCAGAAGACGGCATACGAGATTACTACGTGGCCAACACGTTTTAACGTCRTCCCCDCCTTCCTC | Fbc4 | AATGATACGGCGACCACCGAGATCTACACTGGTCAACGATATACCGGGACTTAGGATTAGATACCCBRGTAGTC |
| 150 | Rrcbc49 | CAAGCAGAAGACGGCATACGAGATGGCCAGTTCCTAAACACGTTTTAACGTCRTCCCCDCCTTCCTC | Fbc4 | AATGATACGGCGACCACCGAGATCTACACTGGTCAACGATATACCGGGACTTAGGATTAGATACCCBRGTAGTC |
| 151 | Rrcbc50 | CAAGCAGAAGACGGCATACGAGATGATGTTCGCTAGAACACGTTTTAACGTCRTCCCCDCCTTCCTC | Fbc4 | AATGATACGGCGACCACCGAGATCTACACTGGTCAACGATATACCGGGACTTAGGATTAGATACCCBRGTAGTC |
| 152 | Rrcbc51 | CAAGCAGAAGACGGCATACGAGATCTATCTCCTGTCAACACGTTTTAACGTCRTCCCCDCCTTCCTC | Fbc4 | AATGATACGGCGACCACCGAGATCTACACTGGTCAACGATATACCGGGACTTAGGATTAGATACCCBRGTAGTC |
| 153 | Rrcbc52 | CAAGCAGAAGACGGCATACGAGATACTCACAGGAATAACACGTTTTAACGTCRTCCCCDCCTTCCTC | Fbc4 | AATGATACGGCGACCACCGAGATCTACACTGGTCAACGATATACCGGGACTTAGGATTAGATACCCBRGTAGTC |
| 154 | Rrcbc53 | CAAGCAGAAGACGGCATACGAGATATGATGAGCCTCAACACGTTTTAACGTCRTCCCCDCCTTCCTC | Fbc4 | AATGATACGGCGACCACCGAGATCTACACTGGTCAACGATATACCGGGACTTAGGATTAGATACCCBRGTAGTC |
| 155 | Rrcbc54 | CAAGCAGAAGACGGCATACGAGATGTCGACAGAGGAAACACGTTTTAACGTCRTCCCCDCCTTCCTC | Fbc4 | AATGATACGGCGACCACCGAGATCTACACTGGTCAACGATATACCGGGACTTAGGATTAGATACCCBRGTAGTC |
| 156 | Rrcbc55 | CAAGCAGAAGACGGCATACGAGATTGTCGCAAATAGAACACGTTTTAACGTCRTCCCCDCCTTCCTC | Fbc4 | AATGATACGGCGACCACCGAGATCTACACTGGTCAACGATATACCGGGACTTAGGATTAGATACCCBRGTAGTC |
| 157 | Rrcbc44 | CAAGCAGAAGACGGCATACGAGATTAATACGGATCGAACACGTTTTAACGTCRTCCCCDCCTTCCTC | Fbc5 | AATGATACGGCGACCACCGAGATCTACACATCGCACAGTAATACCGGGACTTAGGATTAGATACCCBRGTAGTC |
| 158 | Rrcbc45 | CAAGCAGAAGACGGCATACGAGATTCGGAATTAGACAACACGTTTTAACGTCRTCCCCDCCTTCCTC | Fbc5 | AATGATACGGCGACCACCGAGATCTACACATCGCACAGTAATACCGGGACTTAGGATTAGATACCCBRGTAGTC |
| 159 | Rrcbc46 | CAAGCAGAAGACGGCATACGAGATTGTGAATTCGGAAACACGTTTTAACGTCRTCCCCDCCTTCCTC | Fbc5 | AATGATACGGCGACCACCGAGATCTACACATCGCACAGTAATACCGGGACTTAGGATTAGATACCCBRGTAGTC |
| 160 | Rrcbc47 | CAAGCAGAAGACGGCATACGAGATCATTCGTGGCGTAACACGTTTTAACGTCRTCCCCDCCTTCCTC | Fbc5 | AATGATACGGCGACCACCGAGATCTACACATCGCACAGTAATACCGGGACTTAGGATTAGATACCCBRGTAGTC |
| 161 | Rrcbc48 | CAAGCAGAAGACGGCATACGAGATTACTACGTGGCCAACACGTTTTAACGTCRTCCCCDCCTTCCTC | Fbc5 | AATGATACGGCGACCACCGAGATCTACACATCGCACAGTAATACCGGGACTTAGGATTAGATACCCBRGTAGTC |
| 162 | Rrcbc49 | CAAGCAGAAGACGGCATACGAGATGGCCAGTTCCTAAACACGTTTTAACGTCRTCCCCDCCTTCCTC | Fbc5 | AATGATACGGCGACCACCGAGATCTACACATCGCACAGTAATACCGGGACTTAGGATTAGATACCCBRGTAGTC |
| 163 | Rrcbc50 | CAAGCAGAAGACGGCATACGAGATGATGTTCGCTAGAACACGTTTTAACGTCRTCCCCDCCTTCCTC | Fbc5 | AATGATACGGCGACCACCGAGATCTACACATCGCACAGTAATACCGGGACTTAGGATTAGATACCCBRGTAGTC |
| 164 | Rrcbc51 | CAAGCAGAAGACGGCATACGAGATCTATCTCCTGTCAACACGTTTTAACGTCRTCCCCDCCTTCCTC | Fbc5 | AATGATACGGCGACCACCGAGATCTACACATCGCACAGTAATACCGGGACTTAGGATTAGATACCCBRGTAGTC |
| 165 | Rrcbc52 | CAAGCAGAAGACGGCATACGAGATACTCACAGGAATAACACGTTTTAACGTCRTCCCCDCCTTCCTC | Fbc5 | AATGATACGGCGACCACCGAGATCTACACATCGCACAGTAATACCGGGACTTAGGATTAGATACCCBRGTAGTC |
| 166 | Rrcbc53 | CAAGCAGAAGACGGCATACGAGATATGATGAGCCTCAACACGTTTTAACGTCRTCCCCDCCTTCCTC | Fbc5 | AATGATACGGCGACCACCGAGATCTACACATCGCACAGTAATACCGGGACTTAGGATTAGATACCCBRGTAGTC |
| 167 | Rrcbc54 | CAAGCAGAAGACGGCATACGAGATGTCGACAGAGGAAACACGTTTTAACGTCRTCCCCDCCTTCCTC | Fbc5 | AATGATACGGCGACCACCGAGATCTACACATCGCACAGTAATACCGGGACTTAGGATTAGATACCCBRGTAGTC |
| 168 | Rrcbc55 | CAAGCAGAAGACGGCATACGAGATTGTCGCAAATAGAACACGTTTTAACGTCRTCCCCDCCTTCCTC | Fbc5 | AATGATACGGCGACCACCGAGATCTACACATCGCACAGTAATACCGGGACTTAGGATTAGATACCCBRGTAGTC |
| 169 | Rrcbc44 | CAAGCAGAAGACGGCATACGAGATTAATACGGATCGAACACGTTTTAACGTCRTCCCCDCCTTCCTC | Fbc6 | AATGATACGGCGACCACCGAGATCTACACGTCGTGTAGCCTTACCGGGACTTAGGATTAGATACCCBRGTAGTC |
| 170 | Rrcbc45 | CAAGCAGAAGACGGCATACGAGATTCGGAATTAGACAACACGTTTTAACGTCRTCCCCDCCTTCCTC | Fbc6 | AATGATACGGCGACCACCGAGATCTACACGTCGTGTAGCCTTACCGGGACTTAGGATTAGATACCCBRGTAGTC |
| 171 | Rrcbc46 | CAAGCAGAAGACGGCATACGAGATTGTGAATTCGGAAACACGTTTTAACGTCRTCCCCDCCTTCCTC | Fbc6 | AATGATACGGCGACCACCGAGATCTACACGTCGTGTAGCCTTACCGGGACTTAGGATTAGATACCCBRGTAGTC |
| 172 | Rrcbc47 | CAAGCAGAAGACGGCATACGAGATCATTCGTGGCGTAACACGTTTTAACGTCRTCCCCDCCTTCCTC | Fbc6 | AATGATACGGCGACCACCGAGATCTACACGTCGTGTAGCCTTACCGGGACTTAGGATTAGATACCCBRGTAGTC |
| 173 | Rrcbc48 | CAAGCAGAAGACGGCATACGAGATTACTACGTGGCCAACACGTTTTAACGTCRTCCCCDCCTTCCTC | Fbc6 | AATGATACGGCGACCACCGAGATCTACACGTCGTGTAGCCTTACCGGGACTTAGGATTAGATACCCBRGTAGTC |
| 174 | Rrcbc49 | CAAGCAGAAGACGGCATACGAGATGGCCAGTTCCTAAACACGTTTTAACGTCRTCCCCDCCTTCCTC | Fbc6 | AATGATACGGCGACCACCGAGATCTACACGTCGTGTAGCCTTACCGGGACTTAGGATTAGATACCCBRGTAGTC |
| 175 | Rrcbc50 | CAAGCAGAAGACGGCATACGAGATGATGTTCGCTAGAACACGTTTTAACGTCRTCCCCDCCTTCCTC | Fbc6 | AATGATACGGCGACCACCGAGATCTACACGTCGTGTAGCCTTACCGGGACTTAGGATTAGATACCCBRGTAGTC |
| 176 | Rrcbc51 | CAAGCAGAAGACGGCATACGAGATCTATCTCCTGTCAACACGTTTTAACGTCRTCCCCDCCTTCCTC | Fbc6 | AATGATACGGCGACCACCGAGATCTACACGTCGTGTAGCCTTACCGGGACTTAGGATTAGATACCCBRGTAGTC |
| 177 | Rrcbc52 | CAAGCAGAAGACGGCATACGAGATACTCACAGGAATAACACGTTTTAACGTCRTCCCCDCCTTCCTC | Fbc6 | AATGATACGGCGACCACCGAGATCTACACGTCGTGTAGCCTTACCGGGACTTAGGATTAGATACCCBRGTAGTC |
| 178 | Rrcbc53 | CAAGCAGAAGACGGCATACGAGATATGATGAGCCTCAACACGTTTTAACGTCRTCCCCDCCTTCCTC | Fbc6 | AATGATACGGCGACCACCGAGATCTACACGTCGTGTAGCCTTACCGGGACTTAGGATTAGATACCCBRGTAGTC |
| 179 | Rrcbc54 | CAAGCAGAAGACGGCATACGAGATGTCGACAGAGGAAACACGTTTTAACGTCRTCCCCDCCTTCCTC | Fbc6 | AATGATACGGCGACCACCGAGATCTACACGTCGTGTAGCCTTACCGGGACTTAGGATTAGATACCCBRGTAGTC |
| 180 | Rrcbc55 | CAAGCAGAAGACGGCATACGAGATTGTCGCAAATAGAACACGTTTTAACGTCRTCCCCDCCTTCCTC | Fbc6 | AATGATACGGCGACCACCGAGATCTACACGTCGTGTAGCCTTACCGGGACTTAGGATTAGATACCCBRGTAGTC |
| 181 | Rrcbc44 | CAAGCAGAAGACGGCATACGAGATTAATACGGATCGAACACGTTTTAACGTCRTCCCCDCCTTCCTC | Fbc7 | AATGATACGGCGACCACCGAGATCTACACAGCGGAGGTTAGTACCGGGACTTAGGATTAGATACCCBRGTAGTC |
| 182 | Rrcbc45 | CAAGCAGAAGACGGCATACGAGATTCGGAATTAGACAACACGTTTTAACGTCRTCCCCDCCTTCCTC | Fbc7 | AATGATACGGCGACCACCGAGATCTACACAGCGGAGGTTAGTACCGGGACTTAGGATTAGATACCCBRGTAGTC |
| 183 | Rrcbc46 | CAAGCAGAAGACGGCATACGAGATTGTGAATTCGGAAACACGTTTTAACGTCRTCCCCDCCTTCCTC | Fbc7 | AATGATACGGCGACCACCGAGATCTACACAGCGGAGGTTAGTACCGGGACTTAGGATTAGATACCCBRGTAGTC |
| 184 | Rrcbc47 | CAAGCAGAAGACGGCATACGAGATCATTCGTGGCGTAACACGTTTTAACGTCRTCCCCDCCTTCCTC | Fbc7 | AATGATACGGCGACCACCGAGATCTACACAGCGGAGGTTAGTACCGGGACTTAGGATTAGATACCCBRGTAGTC |
| 185 | Rrcbc48 | CAAGCAGAAGACGGCATACGAGATTACTACGTGGCCAACACGTTTTAACGTCRTCCCCDCCTTCCTC | Fbc7 | AATGATACGGCGACCACCGAGATCTACACAGCGGAGGTTAGTACCGGGACTTAGGATTAGATACCCBRGTAGTC |
| 186 | Rrcbc49 | CAAGCAGAAGACGGCATACGAGATGGCCAGTTCCTAAACACGTTTTAACGTCRTCCCCDCCTTCCTC | Fbc7 | AATGATACGGCGACCACCGAGATCTACACAGCGGAGGTTAGTACCGGGACTTAGGATTAGATACCCBRGTAGTC |
| 187 | Rrcbc50 | CAAGCAGAAGACGGCATACGAGATGATGTTCGCTAGAACACGTTTTAACGTCRTCCCCDCCTTCCTC | Fbc7 | AATGATACGGCGACCACCGAGATCTACACAGCGGAGGTTAGTACCGGGACTTAGGATTAGATACCCBRGTAGTC |
| 188 | Rrcbc51 | CAAGCAGAAGACGGCATACGAGATCTATCTCCTGTCAACACGTTTTAACGTCRTCCCCDCCTTCCTC | Fbc7 | AATGATACGGCGACCACCGAGATCTACACAGCGGAGGTTAGTACCGGGACTTAGGATTAGATACCCBRGTAGTC |
| 189 | Rrcbc52 | CAAGCAGAAGACGGCATACGAGATACTCACAGGAATAACACGTTTTAACGTCRTCCCCDCCTTCCTC | Fbc7 | AATGATACGGCGACCACCGAGATCTACACAGCGGAGGTTAGTACCGGGACTTAGGATTAGATACCCBRGTAGTC |
| 190 | Rrcbc53 | CAAGCAGAAGACGGCATACGAGATATGATGAGCCTCAACACGTTTTAACGTCRTCCCCDCCTTCCTC | Fbc7 | AATGATACGGCGACCACCGAGATCTACACAGCGGAGGTTAGTACCGGGACTTAGGATTAGATACCCBRGTAGTC |
| 191 | Rrcbc54 | CAAGCAGAAGACGGCATACGAGATGTCGACAGAGGAAACACGTTTTAACGTCRTCCCCDCCTTCCTC | Fbc7 | AATGATACGGCGACCACCGAGATCTACACAGCGGAGGTTAGTACCGGGACTTAGGATTAGATACCCBRGTAGTC |
| 192 | Rrcbc55 | CAAGCAGAAGACGGCATACGAGATTGTCGCAAATAGAACACGTTTTAACGTCRTCCCCDCCTTCCTC | Fbc7 | AATGATACGGCGACCACCGAGATCTACACAGCGGAGGTTAGTACCGGGACTTAGGATTAGATACCCBRGTAGTC |
| 193 | Rrcbc32 | CAAGCAGAAGACGGCATACGAGATGCATATGCACTGAACACGTTTTAACGTCRTCCCCDCCTTCCTC | Fbc8 | AATGATACGGCGACCACCGAGATCTACACATCCTTTGGTTCTACCGGGACTTAGGATTAGATACCCBRGTAGTC |
| 194 | Rrcbc33 | CAAGCAGAAGACGGCATACGAGATCAACTCCCGTGAAACACGTTTTAACGTCRTCCCCDCCTTCCTC | Fbc8 | AATGATACGGCGACCACCGAGATCTACACATCCTTTGGTTCTACCGGGACTTAGGATTAGATACCCBRGTAGTC |
| 195 | Rrcbc34 | CAAGCAGAAGACGGCATACGAGATTTGCGTTAGCAGAACACGTTTTAACGTCRTCCCCDCCTTCCTC | Fbc8 | AATGATACGGCGACCACCGAGATCTACACATCCTTTGGTTCTACCGGGACTTAGGATTAGATACCCBRGTAGTC |
| 196 | Rrcbc35 | CAAGCAGAAGACGGCATACGAGATTACGAGCCCTAAAACACGTTTTAACGTCRTCCCCDCCTTCCTC | Fbc8 | AATGATACGGCGACCACCGAGATCTACACATCCTTTGGTTCTACCGGGACTTAGGATTAGATACCCBRGTAGTC |
| 197 | Rrcbc36 | CAAGCAGAAGACGGCATACGAGATCACTACGCTAGAAACACGTTTTAACGTCRTCCCCDCCTTCCTC | Fbc8 | AATGATACGGCGACCACCGAGATCTACACATCCTTTGGTTCTACCGGGACTTAGGATTAGATACCCBRGTAGTC |
| 198 | Rrcbc37 | CAAGCAGAAGACGGCATACGAGATTGCAGTCCTCGAAACACGTTTTAACGTCRTCCCCDCCTTCCTC | Fbc8 | AATGATACGGCGACCACCGAGATCTACACATCCTTTGGTTCTACCGGGACTTAGGATTAGATACCCBRGTAGTC |
| 199 | Rrcbc38 | CAAGCAGAAGACGGCATACGAGATACCATAGCTCCGAACACGTTTTAACGTCRTCCCCDCCTTCCTC | Fbc8 | AATGATACGGCGACCACCGAGATCTACACATCCTTTGGTTCTACCGGGACTTAGGATTAGATACCCBRGTAGTC |
| 200 | Rrcbc39 | CAAGCAGAAGACGGCATACGAGATTCGACATCTCTTAACACGTTTTAACGTCRTCCCCDCCTTCCTC | Fbc8 | AATGATACGGCGACCACCGAGATCTACACATCCTTTGGTTCTACCGGGACTTAGGATTAGATACCCBRGTAGTC |
| 201 | Rrcbc40 | CAAGCAGAAGACGGCATACGAGATGAACACTTTGGAAACACGTTTTAACGTCRTCCCCDCCTTCCTC | Fbc8 | AATGATACGGCGACCACCGAGATCTACACATCCTTTGGTTCTACCGGGACTTAGGATTAGATACCCBRGTAGTC |
| 202 | Rrcbc41 | CAAGCAGAAGACGGCATACGAGATGAGCCATCTGTAAACACGTTTTAACGTCRTCCCCDCCTTCCTC | Fbc8 | AATGATACGGCGACCACCGAGATCTACACATCCTTTGGTTCTACCGGGACTTAGGATTAGATACCCBRGTAGTC |
| 203 | Rrcbc42 | CAAGCAGAAGACGGCATACGAGATTTGGGTACACGTAACACGTTTTAACGTCRTCCCCDCCTTCCTC | Fbc8 | AATGATACGGCGACCACCGAGATCTACACATCCTTTGGTTCTACCGGGACTTAGGATTAGATACCCBRGTAGTC |
| 204 | Rrcbc43 | CAAGCAGAAGACGGCATACGAGATAAGGCGCTCCTTAACACGTTTTAACGTCRTCCCCDCCTTCCTC | Fbc8 | AATGATACGGCGACCACCGAGATCTACACATCCTTTGGTTCTACCGGGACTTAGGATTAGATACCCBRGTAGTC |
| 205 | Rrcbc32 | CAAGCAGAAGACGGCATACGAGATGCATATGCACTGAACACGTTTTAACGTCRTCCCCDCCTTCCTC | Fbc9 | AATGATACGGCGACCACCGAGATCTACACTACAGCGCATACTACCGGGACTTAGGATTAGATACCCBRGTAGTC |
| 206 | Rrcbc33 | CAAGCAGAAGACGGCATACGAGATCAACTCCCGTGAAACACGTTTTAACGTCRTCCCCDCCTTCCTC | Fbc9 | AATGATACGGCGACCACCGAGATCTACACTACAGCGCATACTACCGGGACTTAGGATTAGATACCCBRGTAGTC |
| 207 | Rrcbc34 | CAAGCAGAAGACGGCATACGAGATTTGCGTTAGCAGAACACGTTTTAACGTCRTCCCCDCCTTCCTC | Fbc9 | AATGATACGGCGACCACCGAGATCTACACTACAGCGCATACTACCGGGACTTAGGATTAGATACCCBRGTAGTC |
| 208 | Rrcbc35 | CAAGCAGAAGACGGCATACGAGATTACGAGCCCTAAAACACGTTTTAACGTCRTCCCCDCCTTCCTC | Fbc9 | AATGATACGGCGACCACCGAGATCTACACTACAGCGCATACTACCGGGACTTAGGATTAGATACCCBRGTAGTC |
| 209 | Rrcbc36 | CAAGCAGAAGACGGCATACGAGATCACTACGCTAGAAACACGTTTTAACGTCRTCCCCDCCTTCCTC | Fbc9 | AATGATACGGCGACCACCGAGATCTACACTACAGCGCATACTACCGGGACTTAGGATTAGATACCCBRGTAGTC |
| 210 | Rrcbc37 | CAAGCAGAAGACGGCATACGAGATTGCAGTCCTCGAAACACGTTTTAACGTCRTCCCCDCCTTCCTC | Fbc9 | AATGATACGGCGACCACCGAGATCTACACTACAGCGCATACTACCGGGACTTAGGATTAGATACCCBRGTAGTC |
| 211 | Rrcbc38 | CAAGCAGAAGACGGCATACGAGATACCATAGCTCCGAACACGTTTTAACGTCRTCCCCDCCTTCCTC | Fbc9 | AATGATACGGCGACCACCGAGATCTACACTACAGCGCATACTACCGGGACTTAGGATTAGATACCCBRGTAGTC |
| 212 | Rrcbc39 | CAAGCAGAAGACGGCATACGAGATTCGACATCTCTTAACACGTTTTAACGTCRTCCCCDCCTTCCTC | Fbc9 | AATGATACGGCGACCACCGAGATCTACACTACAGCGCATACTACCGGGACTTAGGATTAGATACCCBRGTAGTC |
| 213 | Rrcbc40 | CAAGCAGAAGACGGCATACGAGATGAACACTTTGGAAACACGTTTTAACGTCRTCCCCDCCTTCCTC | Fbc9 | AATGATACGGCGACCACCGAGATCTACACTACAGCGCATACTACCGGGACTTAGGATTAGATACCCBRGTAGTC |
| 214 | Rrcbc41 | CAAGCAGAAGACGGCATACGAGATGAGCCATCTGTAAACACGTTTTAACGTCRTCCCCDCCTTCCTC | Fbc9 | AATGATACGGCGACCACCGAGATCTACACTACAGCGCATACTACCGGGACTTAGGATTAGATACCCBRGTAGTC |
| 215 | Rrcbc42 | CAAGCAGAAGACGGCATACGAGATTTGGGTACACGTAACACGTTTTAACGTCRTCCCCDCCTTCCTC | Fbc9 | AATGATACGGCGACCACCGAGATCTACACTACAGCGCATACTACCGGGACTTAGGATTAGATACCCBRGTAGTC |
| 216 | Rrcbc43 | CAAGCAGAAGACGGCATACGAGATAAGGCGCTCCTTAACACGTTTTAACGTCRTCCCCDCCTTCCTC | Fbc9 | AATGATACGGCGACCACCGAGATCTACACTACAGCGCATACTACCGGGACTTAGGATTAGATACCCBRGTAGTC |
| 217 | Rrcbc32 | CAAGCAGAAGACGGCATACGAGATGCATATGCACTGAACACGTTTTAACGTCRTCCCCDCCTTCCTC | Fbc10 | AATGATACGGCGACCACCGAGATCTACACACCGGTATGTACTACCGGGACTTAGGATTAGATACCCBRGTAGTC |
| 218 | Rrcbc33 | CAAGCAGAAGACGGCATACGAGATCAACTCCCGTGAAACACGTTTTAACGTCRTCCCCDCCTTCCTC | Fbc10 | AATGATACGGCGACCACCGAGATCTACACACCGGTATGTACTACCGGGACTTAGGATTAGATACCCBRGTAGTC |
| 219 | Rrcbc34 | CAAGCAGAAGACGGCATACGAGATTTGCGTTAGCAGAACACGTTTTAACGTCRTCCCCDCCTTCCTC | Fbc10 | AATGATACGGCGACCACCGAGATCTACACACCGGTATGTACTACCGGGACTTAGGATTAGATACCCBRGTAGTC |
| 220 | Rrcbc35 | CAAGCAGAAGACGGCATACGAGATTACGAGCCCTAAAACACGTTTTAACGTCRTCCCCDCCTTCCTC | Fbc10 | AATGATACGGCGACCACCGAGATCTACACACCGGTATGTACTACCGGGACTTAGGATTAGATACCCBRGTAGTC |
| 221 | Rrcbc36 | CAAGCAGAAGACGGCATACGAGATCACTACGCTAGAAACACGTTTTAACGTCRTCCCCDCCTTCCTC | Fbc10 | AATGATACGGCGACCACCGAGATCTACACACCGGTATGTACTACCGGGACTTAGGATTAGATACCCBRGTAGTC |
| 222 | Rrcbc37 | CAAGCAGAAGACGGCATACGAGATTGCAGTCCTCGAAACACGTTTTAACGTCRTCCCCDCCTTCCTC | Fbc10 | AATGATACGGCGACCACCGAGATCTACACACCGGTATGTACTACCGGGACTTAGGATTAGATACCCBRGTAGTC |
| 223 | Rrcbc38 | CAAGCAGAAGACGGCATACGAGATACCATAGCTCCGAACACGTTTTAACGTCRTCCCCDCCTTCCTC | Fbc10 | AATGATACGGCGACCACCGAGATCTACACACCGGTATGTACTACCGGGACTTAGGATTAGATACCCBRGTAGTC |
| 224 | Rrcbc39 | CAAGCAGAAGACGGCATACGAGATTCGACATCTCTTAACACGTTTTAACGTCRTCCCCDCCTTCCTC | Fbc10 | AATGATACGGCGACCACCGAGATCTACACACCGGTATGTACTACCGGGACTTAGGATTAGATACCCBRGTAGTC |
| 225 | Rrcbc40 | CAAGCAGAAGACGGCATACGAGATGAACACTTTGGAAACACGTTTTAACGTCRTCCCCDCCTTCCTC | Fbc10 | AATGATACGGCGACCACCGAGATCTACACACCGGTATGTACTACCGGGACTTAGGATTAGATACCCBRGTAGTC |
| 226 | Rrcbc41 | CAAGCAGAAGACGGCATACGAGATGAGCCATCTGTAAACACGTTTTAACGTCRTCCCCDCCTTCCTC | Fbc10 | AATGATACGGCGACCACCGAGATCTACACACCGGTATGTACTACCGGGACTTAGGATTAGATACCCBRGTAGTC |
| 227 | Rrcbc42 | CAAGCAGAAGACGGCATACGAGATTTGGGTACACGTAACACGTTTTAACGTCRTCCCCDCCTTCCTC | Fbc10 | AATGATACGGCGACCACCGAGATCTACACACCGGTATGTACTACCGGGACTTAGGATTAGATACCCBRGTAGTC |
| 228 | Rrcbc43 | CAAGCAGAAGACGGCATACGAGATAAGGCGCTCCTTAACACGTTTTAACGTCRTCCCCDCCTTCCTC | Fbc10 | AATGATACGGCGACCACCGAGATCTACACACCGGTATGTACTACCGGGACTTAGGATTAGATACCCBRGTAGTC |
| 229 | Rrcbc32 | CAAGCAGAAGACGGCATACGAGATGCATATGCACTGAACACGTTTTAACGTCRTCCCCDCCTTCCTC | Fbc11 | AATGATACGGCGACCACCGAGATCTACACAATTGTGTCGGATACCGGGACTTAGGATTAGATACCCBRGTAGTC |
| 230 | Rrcbc33 | CAAGCAGAAGACGGCATACGAGATCAACTCCCGTGAAACACGTTTTAACGTCRTCCCCDCCTTCCTC | Fbc11 | AATGATACGGCGACCACCGAGATCTACACAATTGTGTCGGATACCGGGACTTAGGATTAGATACCCBRGTAGTC |
| 231 | Rrcbc34 | CAAGCAGAAGACGGCATACGAGATTTGCGTTAGCAGAACACGTTTTAACGTCRTCCCCDCCTTCCTC | Fbc11 | AATGATACGGCGACCACCGAGATCTACACAATTGTGTCGGATACCGGGACTTAGGATTAGATACCCBRGTAGTC |
| 232 | Rrcbc35 | CAAGCAGAAGACGGCATACGAGATTACGAGCCCTAAAACACGTTTTAACGTCRTCCCCDCCTTCCTC | Fbc11 | AATGATACGGCGACCACCGAGATCTACACAATTGTGTCGGATACCGGGACTTAGGATTAGATACCCBRGTAGTC |
| 233 | Rrcbc36 | CAAGCAGAAGACGGCATACGAGATCACTACGCTAGAAACACGTTTTAACGTCRTCCCCDCCTTCCTC | Fbc11 | AATGATACGGCGACCACCGAGATCTACACAATTGTGTCGGATACCGGGACTTAGGATTAGATACCCBRGTAGTC |
| 234 | Rrcbc37 | CAAGCAGAAGACGGCATACGAGATTGCAGTCCTCGAAACACGTTTTAACGTCRTCCCCDCCTTCCTC | Fbc11 | AATGATACGGCGACCACCGAGATCTACACAATTGTGTCGGATACCGGGACTTAGGATTAGATACCCBRGTAGTC |
| 235 | Rrcbc38 | CAAGCAGAAGACGGCATACGAGATACCATAGCTCCGAACACGTTTTAACGTCRTCCCCDCCTTCCTC | Fbc11 | AATGATACGGCGACCACCGAGATCTACACAATTGTGTCGGATACCGGGACTTAGGATTAGATACCCBRGTAGTC |
| 236 | Rrcbc39 | CAAGCAGAAGACGGCATACGAGATTCGACATCTCTTAACACGTTTTAACGTCRTCCCCDCCTTCCTC | Fbc11 | AATGATACGGCGACCACCGAGATCTACACAATTGTGTCGGATACCGGGACTTAGGATTAGATACCCBRGTAGTC |
| 237 | Rrcbc40 | CAAGCAGAAGACGGCATACGAGATGAACACTTTGGAAACACGTTTTAACGTCRTCCCCDCCTTCCTC | Fbc11 | AATGATACGGCGACCACCGAGATCTACACAATTGTGTCGGATACCGGGACTTAGGATTAGATACCCBRGTAGTC |
| 238 | Rrcbc41 | CAAGCAGAAGACGGCATACGAGATGAGCCATCTGTAAACACGTTTTAACGTCRTCCCCDCCTTCCTC | Fbc11 | AATGATACGGCGACCACCGAGATCTACACAATTGTGTCGGATACCGGGACTTAGGATTAGATACCCBRGTAGTC |
| 239 | Rrcbc42 | CAAGCAGAAGACGGCATACGAGATTTGGGTACACGTAACACGTTTTAACGTCRTCCCCDCCTTCCTC | Fbc11 | AATGATACGGCGACCACCGAGATCTACACAATTGTGTCGGATACCGGGACTTAGGATTAGATACCCBRGTAGTC |
| 240 | Rrcbc43 | CAAGCAGAAGACGGCATACGAGATAAGGCGCTCCTTAACACGTTTTAACGTCRTCCCCDCCTTCCTC | Fbc11 | AATGATACGGCGACCACCGAGATCTACACAATTGTGTCGGATACCGGGACTTAGGATTAGATACCCBRGTAGTC |
| 241 | Rrcbc32 | CAAGCAGAAGACGGCATACGAGATGCATATGCACTGAACACGTTTTAACGTCRTCCCCDCCTTCCTC | Fbc12 | AATGATACGGCGACCACCGAGATCTACACTGCATACACTGGTACCGGGACTTAGGATTAGATACCCBRGTAGTC |
| 242 | Rrcbc33 | CAAGCAGAAGACGGCATACGAGATCAACTCCCGTGAAACACGTTTTAACGTCRTCCCCDCCTTCCTC | Fbc12 | AATGATACGGCGACCACCGAGATCTACACTGCATACACTGGTACCGGGACTTAGGATTAGATACCCBRGTAGTC |
| 243 | Rrcbc34 | CAAGCAGAAGACGGCATACGAGATTTGCGTTAGCAGAACACGTTTTAACGTCRTCCCCDCCTTCCTC | Fbc12 | AATGATACGGCGACCACCGAGATCTACACTGCATACACTGGTACCGGGACTTAGGATTAGATACCCBRGTAGTC |
| 244 | Rrcbc35 | CAAGCAGAAGACGGCATACGAGATTACGAGCCCTAAAACACGTTTTAACGTCRTCCCCDCCTTCCTC | Fbc12 | AATGATACGGCGACCACCGAGATCTACACTGCATACACTGGTACCGGGACTTAGGATTAGATACCCBRGTAGTC |
| 245 | Rrcbc36 | CAAGCAGAAGACGGCATACGAGATCACTACGCTAGAAACACGTTTTAACGTCRTCCCCDCCTTCCTC | Fbc12 | AATGATACGGCGACCACCGAGATCTACACTGCATACACTGGTACCGGGACTTAGGATTAGATACCCBRGTAGTC |
| 246 | Rrcbc37 | CAAGCAGAAGACGGCATACGAGATTGCAGTCCTCGAAACACGTTTTAACGTCRTCCCCDCCTTCCTC | Fbc12 | AATGATACGGCGACCACCGAGATCTACACTGCATACACTGGTACCGGGACTTAGGATTAGATACCCBRGTAGTC |
| 247 | Rrcbc38 | CAAGCAGAAGACGGCATACGAGATACCATAGCTCCGAACACGTTTTAACGTCRTCCCCDCCTTCCTC | Fbc12 | AATGATACGGCGACCACCGAGATCTACACTGCATACACTGGTACCGGGACTTAGGATTAGATACCCBRGTAGTC |
| 248 | Rrcbc39 | CAAGCAGAAGACGGCATACGAGATTCGACATCTCTTAACACGTTTTAACGTCRTCCCCDCCTTCCTC | Fbc12 | AATGATACGGCGACCACCGAGATCTACACTGCATACACTGGTACCGGGACTTAGGATTAGATACCCBRGTAGTC |
| 249 | Rrcbc40 | CAAGCAGAAGACGGCATACGAGATGAACACTTTGGAAACACGTTTTAACGTCRTCCCCDCCTTCCTC | Fbc12 | AATGATACGGCGACCACCGAGATCTACACTGCATACACTGGTACCGGGACTTAGGATTAGATACCCBRGTAGTC |
| 250 | Rrcbc41 | CAAGCAGAAGACGGCATACGAGATGAGCCATCTGTAAACACGTTTTAACGTCRTCCCCDCCTTCCTC | Fbc12 | AATGATACGGCGACCACCGAGATCTACACTGCATACACTGGTACCGGGACTTAGGATTAGATACCCBRGTAGTC |
| 251 | Rrcbc42 | CAAGCAGAAGACGGCATACGAGATTTGGGTACACGTAACACGTTTTAACGTCRTCCCCDCCTTCCTC | Fbc12 | AATGATACGGCGACCACCGAGATCTACACTGCATACACTGGTACCGGGACTTAGGATTAGATACCCBRGTAGTC |
| 252 | Rrcbc43 | CAAGCAGAAGACGGCATACGAGATAAGGCGCTCCTTAACACGTTTTAACGTCRTCCCCDCCTTCCTC | Fbc12 | AATGATACGGCGACCACCGAGATCTACACTGCATACACTGGTACCGGGACTTAGGATTAGATACCCBRGTAGTC |
| 253 | Rrcbc32 | CAAGCAGAAGACGGCATACGAGATGCATATGCACTGAACACGTTTTAACGTCRTCCCCDCCTTCCTC | Fbc13 | AATGATACGGCGACCACCGAGATCTACACAGTCGAACGAGGTACCGGGACTTAGGATTAGATACCCBRGTAGTC |
| 254 | Rrcbc33 | CAAGCAGAAGACGGCATACGAGATCAACTCCCGTGAAACACGTTTTAACGTCRTCCCCDCCTTCCTC | Fbc13 | AATGATACGGCGACCACCGAGATCTACACAGTCGAACGAGGTACCGGGACTTAGGATTAGATACCCBRGTAGTC |
| 255 | Rrcbc34 | CAAGCAGAAGACGGCATACGAGATTTGCGTTAGCAGAACACGTTTTAACGTCRTCCCCDCCTTCCTC | Fbc13 | AATGATACGGCGACCACCGAGATCTACACAGTCGAACGAGGTACCGGGACTTAGGATTAGATACCCBRGTAGTC |
| 256 | Rrcbc35 | CAAGCAGAAGACGGCATACGAGATTACGAGCCCTAAAACACGTTTTAACGTCRTCCCCDCCTTCCTC | Fbc13 | AATGATACGGCGACCACCGAGATCTACACAGTCGAACGAGGTACCGGGACTTAGGATTAGATACCCBRGTAGTC |
| 257 | Rrcbc36 | CAAGCAGAAGACGGCATACGAGATCACTACGCTAGAAACACGTTTTAACGTCRTCCCCDCCTTCCTC | Fbc13 | AATGATACGGCGACCACCGAGATCTACACAGTCGAACGAGGTACCGGGACTTAGGATTAGATACCCBRGTAGTC |
| 258 | Rrcbc37 | CAAGCAGAAGACGGCATACGAGATTGCAGTCCTCGAAACACGTTTTAACGTCRTCCCCDCCTTCCTC | Fbc13 | AATGATACGGCGACCACCGAGATCTACACAGTCGAACGAGGTACCGGGACTTAGGATTAGATACCCBRGTAGTC |
| 259 | Rrcbc38 | CAAGCAGAAGACGGCATACGAGATACCATAGCTCCGAACACGTTTTAACGTCRTCCCCDCCTTCCTC | Fbc13 | AATGATACGGCGACCACCGAGATCTACACAGTCGAACGAGGTACCGGGACTTAGGATTAGATACCCBRGTAGTC |
| 260 | Rrcbc39 | CAAGCAGAAGACGGCATACGAGATTCGACATCTCTTAACACGTTTTAACGTCRTCCCCDCCTTCCTC | Fbc13 | AATGATACGGCGACCACCGAGATCTACACAGTCGAACGAGGTACCGGGACTTAGGATTAGATACCCBRGTAGTC |
| 261 | Rrcbc40 | CAAGCAGAAGACGGCATACGAGATGAACACTTTGGAAACACGTTTTAACGTCRTCCCCDCCTTCCTC | Fbc13 | AATGATACGGCGACCACCGAGATCTACACAGTCGAACGAGGTACCGGGACTTAGGATTAGATACCCBRGTAGTC |
| 262 | Rrcbc41 | CAAGCAGAAGACGGCATACGAGATGAGCCATCTGTAAACACGTTTTAACGTCRTCCCCDCCTTCCTC | Fbc13 | AATGATACGGCGACCACCGAGATCTACACAGTCGAACGAGGTACCGGGACTTAGGATTAGATACCCBRGTAGTC |
| 263 | Rrcbc42 | CAAGCAGAAGACGGCATACGAGATTTGGGTACACGTAACACGTTTTAACGTCRTCCCCDCCTTCCTC | Fbc13 | AATGATACGGCGACCACCGAGATCTACACAGTCGAACGAGGTACCGGGACTTAGGATTAGATACCCBRGTAGTC |
| 264 | Rrcbc43 | CAAGCAGAAGACGGCATACGAGATAAGGCGCTCCTTAACACGTTTTAACGTCRTCCCCDCCTTCCTC | Fbc13 | AATGATACGGCGACCACCGAGATCTACACAGTCGAACGAGGTACCGGGACTTAGGATTAGATACCCBRGTAGTC |
| 265 | Rrcbc32 | CAAGCAGAAGACGGCATACGAGATGCATATGCACTGAACACGTTTTAACGTCRTCCCCDCCTTCCTC | Fbc14 | AATGATACGGCGACCACCGAGATCTACACACCAGTGACTCATACCGGGACTTAGGATTAGATACCCBRGTAGTC |
| 266 | Rrcbc33 | CAAGCAGAAGACGGCATACGAGATCAACTCCCGTGAAACACGTTTTAACGTCRTCCCCDCCTTCCTC | Fbc14 | AATGATACGGCGACCACCGAGATCTACACACCAGTGACTCATACCGGGACTTAGGATTAGATACCCBRGTAGTC |
| 267 | Rrcbc34 | CAAGCAGAAGACGGCATACGAGATTTGCGTTAGCAGAACACGTTTTAACGTCRTCCCCDCCTTCCTC | Fbc14 | AATGATACGGCGACCACCGAGATCTACACACCAGTGACTCATACCGGGACTTAGGATTAGATACCCBRGTAGTC |
| 268 | Rrcbc35 | CAAGCAGAAGACGGCATACGAGATTACGAGCCCTAAAACACGTTTTAACGTCRTCCCCDCCTTCCTC | Fbc14 | AATGATACGGCGACCACCGAGATCTACACACCAGTGACTCATACCGGGACTTAGGATTAGATACCCBRGTAGTC |
| 269 | Rrcbc36 | CAAGCAGAAGACGGCATACGAGATCACTACGCTAGAAACACGTTTTAACGTCRTCCCCDCCTTCCTC | Fbc14 | AATGATACGGCGACCACCGAGATCTACACACCAGTGACTCATACCGGGACTTAGGATTAGATACCCBRGTAGTC |
| 270 | Rrcbc37 | CAAGCAGAAGACGGCATACGAGATTGCAGTCCTCGAAACACGTTTTAACGTCRTCCCCDCCTTCCTC | Fbc14 | AATGATACGGCGACCACCGAGATCTACACACCAGTGACTCATACCGGGACTTAGGATTAGATACCCBRGTAGTC |
| 271 | Rrcbc38 | CAAGCAGAAGACGGCATACGAGATACCATAGCTCCGAACACGTTTTAACGTCRTCCCCDCCTTCCTC | Fbc14 | AATGATACGGCGACCACCGAGATCTACACACCAGTGACTCATACCGGGACTTAGGATTAGATACCCBRGTAGTC |
| 272 | Rrcbc39 | CAAGCAGAAGACGGCATACGAGATTCGACATCTCTTAACACGTTTTAACGTCRTCCCCDCCTTCCTC | Fbc14 | AATGATACGGCGACCACCGAGATCTACACACCAGTGACTCATACCGGGACTTAGGATTAGATACCCBRGTAGTC |
| 273 | Rrcbc40 | CAAGCAGAAGACGGCATACGAGATGAACACTTTGGAAACACGTTTTAACGTCRTCCCCDCCTTCCTC | Fbc14 | AATGATACGGCGACCACCGAGATCTACACACCAGTGACTCATACCGGGACTTAGGATTAGATACCCBRGTAGTC |
| 274 | Rrcbc41 | CAAGCAGAAGACGGCATACGAGATGAGCCATCTGTAAACACGTTTTAACGTCRTCCCCDCCTTCCTC | Fbc14 | AATGATACGGCGACCACCGAGATCTACACACCAGTGACTCATACCGGGACTTAGGATTAGATACCCBRGTAGTC |
| 275 | Rrcbc42 | CAAGCAGAAGACGGCATACGAGATTTGGGTACACGTAACACGTTTTAACGTCRTCCCCDCCTTCCTC | Fbc14 | AATGATACGGCGACCACCGAGATCTACACACCAGTGACTCATACCGGGACTTAGGATTAGATACCCBRGTAGTC |
| 276 | Rrcbc43 | CAAGCAGAAGACGGCATACGAGATAAGGCGCTCCTTAACACGTTTTAACGTCRTCCCCDCCTTCCTC | Fbc14 | AATGATACGGCGACCACCGAGATCTACACACCAGTGACTCATACCGGGACTTAGGATTAGATACCCBRGTAGTC |
| 277 | Rrcbc32 | CAAGCAGAAGACGGCATACGAGATGCATATGCACTGAACACGTTTTAACGTCRTCCCCDCCTTCCTC | Fbc15 | AATGATACGGCGACCACCGAGATCTACACGAATACCAAGTCTACCGGGACTTAGGATTAGATACCCBRGTAGTC |
| 278 | Rrcbc33 | CAAGCAGAAGACGGCATACGAGATCAACTCCCGTGAAACACGTTTTAACGTCRTCCCCDCCTTCCTC | Fbc15 | AATGATACGGCGACCACCGAGATCTACACGAATACCAAGTCTACCGGGACTTAGGATTAGATACCCBRGTAGTC |
| 279 | Rrcbc34 | CAAGCAGAAGACGGCATACGAGATTTGCGTTAGCAGAACACGTTTTAACGTCRTCCCCDCCTTCCTC | Fbc15 | AATGATACGGCGACCACCGAGATCTACACGAATACCAAGTCTACCGGGACTTAGGATTAGATACCCBRGTAGTC |
| 280 | Rrcbc35 | CAAGCAGAAGACGGCATACGAGATTACGAGCCCTAAAACACGTTTTAACGTCRTCCCCDCCTTCCTC | Fbc15 | AATGATACGGCGACCACCGAGATCTACACGAATACCAAGTCTACCGGGACTTAGGATTAGATACCCBRGTAGTC |
| 281 | Rrcbc36 | CAAGCAGAAGACGGCATACGAGATCACTACGCTAGAAACACGTTTTAACGTCRTCCCCDCCTTCCTC | Fbc15 | AATGATACGGCGACCACCGAGATCTACACGAATACCAAGTCTACCGGGACTTAGGATTAGATACCCBRGTAGTC |
| 282 | Rrcbc37 | CAAGCAGAAGACGGCATACGAGATTGCAGTCCTCGAAACACGTTTTAACGTCRTCCCCDCCTTCCTC | Fbc15 | AATGATACGGCGACCACCGAGATCTACACGAATACCAAGTCTACCGGGACTTAGGATTAGATACCCBRGTAGTC |
| 283 | Rrcbc38 | CAAGCAGAAGACGGCATACGAGATACCATAGCTCCGAACACGTTTTAACGTCRTCCCCDCCTTCCTC | Fbc15 | AATGATACGGCGACCACCGAGATCTACACGAATACCAAGTCTACCGGGACTTAGGATTAGATACCCBRGTAGTC |
| 284 | Rrcbc39 | CAAGCAGAAGACGGCATACGAGATTCGACATCTCTTAACACGTTTTAACGTCRTCCCCDCCTTCCTC | Fbc15 | AATGATACGGCGACCACCGAGATCTACACGAATACCAAGTCTACCGGGACTTAGGATTAGATACCCBRGTAGTC |
| 285 | Rrcbc40 | CAAGCAGAAGACGGCATACGAGATGAACACTTTGGAAACACGTTTTAACGTCRTCCCCDCCTTCCTC | Fbc15 | AATGATACGGCGACCACCGAGATCTACACGAATACCAAGTCTACCGGGACTTAGGATTAGATACCCBRGTAGTC |
| 286 | Rrcbc41 | CAAGCAGAAGACGGCATACGAGATGAGCCATCTGTAAACACGTTTTAACGTCRTCCCCDCCTTCCTC | Fbc15 | AATGATACGGCGACCACCGAGATCTACACGAATACCAAGTCTACCGGGACTTAGGATTAGATACCCBRGTAGTC |
| 287 | Rrcbc42 | CAAGCAGAAGACGGCATACGAGATTTGGGTACACGTAACACGTTTTAACGTCRTCCCCDCCTTCCTC | Fbc15 | AATGATACGGCGACCACCGAGATCTACACGAATACCAAGTCTACCGGGACTTAGGATTAGATACCCBRGTAGTC |
| 288 | Rrcbc43 | CAAGCAGAAGACGGCATACGAGATAAGGCGCTCCTTAACACGTTTTAACGTCRTCCCCDCCTTCCTC | Fbc15 | AATGATACGGCGACCACCGAGATCTACACGAATACCAAGTCTACCGGGACTTAGGATTAGATACCCBRGTAGTC |
| 289 | Rrcbc44 | CAAGCAGAAGACGGCATACGAGATTAATACGGATCGAACACGTTTTAACGTCRTCCCCDCCTTCCTC | Fbc8 | AATGATACGGCGACCACCGAGATCTACACATCCTTTGGTTCTACCGGGACTTAGGATTAGATACCCBRGTAGTC |
| 290 | Rrcbc45 | CAAGCAGAAGACGGCATACGAGATTCGGAATTAGACAACACGTTTTAACGTCRTCCCCDCCTTCCTC | Fbc8 | AATGATACGGCGACCACCGAGATCTACACATCCTTTGGTTCTACCGGGACTTAGGATTAGATACCCBRGTAGTC |
| 291 | Rrcbc46 | CAAGCAGAAGACGGCATACGAGATTGTGAATTCGGAAACACGTTTTAACGTCRTCCCCDCCTTCCTC | Fbc8 | AATGATACGGCGACCACCGAGATCTACACATCCTTTGGTTCTACCGGGACTTAGGATTAGATACCCBRGTAGTC |
| 292 | Rrcbc47 | CAAGCAGAAGACGGCATACGAGATCATTCGTGGCGTAACACGTTTTAACGTCRTCCCCDCCTTCCTC | Fbc8 | AATGATACGGCGACCACCGAGATCTACACATCCTTTGGTTCTACCGGGACTTAGGATTAGATACCCBRGTAGTC |
| 293 | Rrcbc48 | CAAGCAGAAGACGGCATACGAGATTACTACGTGGCCAACACGTTTTAACGTCRTCCCCDCCTTCCTC | Fbc8 | AATGATACGGCGACCACCGAGATCTACACATCCTTTGGTTCTACCGGGACTTAGGATTAGATACCCBRGTAGTC |
| 294 | Rrcbc49 | CAAGCAGAAGACGGCATACGAGATGGCCAGTTCCTAAACACGTTTTAACGTCRTCCCCDCCTTCCTC | Fbc8 | AATGATACGGCGACCACCGAGATCTACACATCCTTTGGTTCTACCGGGACTTAGGATTAGATACCCBRGTAGTC |
| 295 | Rrcbc50 | CAAGCAGAAGACGGCATACGAGATGATGTTCGCTAGAACACGTTTTAACGTCRTCCCCDCCTTCCTC | Fbc8 | AATGATACGGCGACCACCGAGATCTACACATCCTTTGGTTCTACCGGGACTTAGGATTAGATACCCBRGTAGTC |
| 296 | Rrcbc51 | CAAGCAGAAGACGGCATACGAGATCTATCTCCTGTCAACACGTTTTAACGTCRTCCCCDCCTTCCTC | Fbc8 | AATGATACGGCGACCACCGAGATCTACACATCCTTTGGTTCTACCGGGACTTAGGATTAGATACCCBRGTAGTC |
| 297 | Rrcbc52 | CAAGCAGAAGACGGCATACGAGATACTCACAGGAATAACACGTTTTAACGTCRTCCCCDCCTTCCTC | Fbc8 | AATGATACGGCGACCACCGAGATCTACACATCCTTTGGTTCTACCGGGACTTAGGATTAGATACCCBRGTAGTC |
| 298 | Rrcbc53 | CAAGCAGAAGACGGCATACGAGATATGATGAGCCTCAACACGTTTTAACGTCRTCCCCDCCTTCCTC | Fbc8 | AATGATACGGCGACCACCGAGATCTACACATCCTTTGGTTCTACCGGGACTTAGGATTAGATACCCBRGTAGTC |
| 299 | Rrcbc54 | CAAGCAGAAGACGGCATACGAGATGTCGACAGAGGAAACACGTTTTAACGTCRTCCCCDCCTTCCTC | Fbc8 | AATGATACGGCGACCACCGAGATCTACACATCCTTTGGTTCTACCGGGACTTAGGATTAGATACCCBRGTAGTC |
| 300 | Rrcbc55 | CAAGCAGAAGACGGCATACGAGATTGTCGCAAATAGAACACGTTTTAACGTCRTCCCCDCCTTCCTC | Fbc8 | AATGATACGGCGACCACCGAGATCTACACATCCTTTGGTTCTACCGGGACTTAGGATTAGATACCCBRGTAGTC |
| 301 | Rrcbc44 | CAAGCAGAAGACGGCATACGAGATTAATACGGATCGAACACGTTTTAACGTCRTCCCCDCCTTCCTC | Fbc9 | AATGATACGGCGACCACCGAGATCTACACTACAGCGCATACTACCGGGACTTAGGATTAGATACCCBRGTAGTC |
| 302 | Rrcbc45 | CAAGCAGAAGACGGCATACGAGATTCGGAATTAGACAACACGTTTTAACGTCRTCCCCDCCTTCCTC | Fbc9 | AATGATACGGCGACCACCGAGATCTACACTACAGCGCATACTACCGGGACTTAGGATTAGATACCCBRGTAGTC |
| 303 | Rrcbc46 | CAAGCAGAAGACGGCATACGAGATTGTGAATTCGGAAACACGTTTTAACGTCRTCCCCDCCTTCCTC | Fbc9 | AATGATACGGCGACCACCGAGATCTACACTACAGCGCATACTACCGGGACTTAGGATTAGATACCCBRGTAGTC |
| 304 | Rrcbc47 | CAAGCAGAAGACGGCATACGAGATCATTCGTGGCGTAACACGTTTTAACGTCRTCCCCDCCTTCCTC | Fbc9 | AATGATACGGCGACCACCGAGATCTACACTACAGCGCATACTACCGGGACTTAGGATTAGATACCCBRGTAGTC |
| 305 | Rrcbc48 | CAAGCAGAAGACGGCATACGAGATTACTACGTGGCCAACACGTTTTAACGTCRTCCCCDCCTTCCTC | Fbc9 | AATGATACGGCGACCACCGAGATCTACACTACAGCGCATACTACCGGGACTTAGGATTAGATACCCBRGTAGTC |
| 306 | Rrcbc49 | CAAGCAGAAGACGGCATACGAGATGGCCAGTTCCTAAACACGTTTTAACGTCRTCCCCDCCTTCCTC | Fbc9 | AATGATACGGCGACCACCGAGATCTACACTACAGCGCATACTACCGGGACTTAGGATTAGATACCCBRGTAGTC |
| 307 | Rrcbc50 | CAAGCAGAAGACGGCATACGAGATGATGTTCGCTAGAACACGTTTTAACGTCRTCCCCDCCTTCCTC | Fbc9 | AATGATACGGCGACCACCGAGATCTACACTACAGCGCATACTACCGGGACTTAGGATTAGATACCCBRGTAGTC |
| 308 | Rrcbc51 | CAAGCAGAAGACGGCATACGAGATCTATCTCCTGTCAACACGTTTTAACGTCRTCCCCDCCTTCCTC | Fbc9 | AATGATACGGCGACCACCGAGATCTACACTACAGCGCATACTACCGGGACTTAGGATTAGATACCCBRGTAGTC |
| 309 | Rrcbc52 | CAAGCAGAAGACGGCATACGAGATACTCACAGGAATAACACGTTTTAACGTCRTCCCCDCCTTCCTC | Fbc9 | AATGATACGGCGACCACCGAGATCTACACTACAGCGCATACTACCGGGACTTAGGATTAGATACCCBRGTAGTC |
| 310 | Rrcbc53 | CAAGCAGAAGACGGCATACGAGATATGATGAGCCTCAACACGTTTTAACGTCRTCCCCDCCTTCCTC | Fbc9 | AATGATACGGCGACCACCGAGATCTACACTACAGCGCATACTACCGGGACTTAGGATTAGATACCCBRGTAGTC |
| 311 | Rrcbc54 | CAAGCAGAAGACGGCATACGAGATGTCGACAGAGGAAACACGTTTTAACGTCRTCCCCDCCTTCCTC | Fbc9 | AATGATACGGCGACCACCGAGATCTACACTACAGCGCATACTACCGGGACTTAGGATTAGATACCCBRGTAGTC |
| 312 | Rrcbc55 | CAAGCAGAAGACGGCATACGAGATTGTCGCAAATAGAACACGTTTTAACGTCRTCCCCDCCTTCCTC | Fbc9 | AATGATACGGCGACCACCGAGATCTACACTACAGCGCATACTACCGGGACTTAGGATTAGATACCCBRGTAGTC |
| 313 | Rrcbc44 | CAAGCAGAAGACGGCATACGAGATTAATACGGATCGAACACGTTTTAACGTCRTCCCCDCCTTCCTC | Fbc10 | AATGATACGGCGACCACCGAGATCTACACACCGGTATGTACTACCGGGACTTAGGATTAGATACCCBRGTAGTC |
| 314 | Rrcbc45 | CAAGCAGAAGACGGCATACGAGATTCGGAATTAGACAACACGTTTTAACGTCRTCCCCDCCTTCCTC | Fbc10 | AATGATACGGCGACCACCGAGATCTACACACCGGTATGTACTACCGGGACTTAGGATTAGATACCCBRGTAGTC |
| 315 | Rrcbc46 | CAAGCAGAAGACGGCATACGAGATTGTGAATTCGGAAACACGTTTTAACGTCRTCCCCDCCTTCCTC | Fbc10 | AATGATACGGCGACCACCGAGATCTACACACCGGTATGTACTACCGGGACTTAGGATTAGATACCCBRGTAGTC |
| 316 | Rrcbc47 | CAAGCAGAAGACGGCATACGAGATCATTCGTGGCGTAACACGTTTTAACGTCRTCCCCDCCTTCCTC | Fbc10 | AATGATACGGCGACCACCGAGATCTACACACCGGTATGTACTACCGGGACTTAGGATTAGATACCCBRGTAGTC |
| 317 | Rrcbc48 | CAAGCAGAAGACGGCATACGAGATTACTACGTGGCCAACACGTTTTAACGTCRTCCCCDCCTTCCTC | Fbc10 | AATGATACGGCGACCACCGAGATCTACACACCGGTATGTACTACCGGGACTTAGGATTAGATACCCBRGTAGTC |
| 318 | Rrcbc49 | CAAGCAGAAGACGGCATACGAGATGGCCAGTTCCTAAACACGTTTTAACGTCRTCCCCDCCTTCCTC | Fbc10 | AATGATACGGCGACCACCGAGATCTACACACCGGTATGTACTACCGGGACTTAGGATTAGATACCCBRGTAGTC |
| 319 | Rrcbc50 | CAAGCAGAAGACGGCATACGAGATGATGTTCGCTAGAACACGTTTTAACGTCRTCCCCDCCTTCCTC | Fbc10 | AATGATACGGCGACCACCGAGATCTACACACCGGTATGTACTACCGGGACTTAGGATTAGATACCCBRGTAGTC |
| 320 | Rrcbc51 | CAAGCAGAAGACGGCATACGAGATCTATCTCCTGTCAACACGTTTTAACGTCRTCCCCDCCTTCCTC | Fbc10 | AATGATACGGCGACCACCGAGATCTACACACCGGTATGTACTACCGGGACTTAGGATTAGATACCCBRGTAGTC |
| 321 | Rrcbc52 | CAAGCAGAAGACGGCATACGAGATACTCACAGGAATAACACGTTTTAACGTCRTCCCCDCCTTCCTC | Fbc10 | AATGATACGGCGACCACCGAGATCTACACACCGGTATGTACTACCGGGACTTAGGATTAGATACCCBRGTAGTC |
| 322 | Rrcbc53 | CAAGCAGAAGACGGCATACGAGATATGATGAGCCTCAACACGTTTTAACGTCRTCCCCDCCTTCCTC | Fbc10 | AATGATACGGCGACCACCGAGATCTACACACCGGTATGTACTACCGGGACTTAGGATTAGATACCCBRGTAGTC |
| 323 | Rrcbc54 | CAAGCAGAAGACGGCATACGAGATGTCGACAGAGGAAACACGTTTTAACGTCRTCCCCDCCTTCCTC | Fbc10 | AATGATACGGCGACCACCGAGATCTACACACCGGTATGTACTACCGGGACTTAGGATTAGATACCCBRGTAGTC |
| 324 | Rrcbc55 | CAAGCAGAAGACGGCATACGAGATTGTCGCAAATAGAACACGTTTTAACGTCRTCCCCDCCTTCCTC | Fbc10 | AATGATACGGCGACCACCGAGATCTACACACCGGTATGTACTACCGGGACTTAGGATTAGATACCCBRGTAGTC |
| 325 | Rrcbc44 | CAAGCAGAAGACGGCATACGAGATTAATACGGATCGAACACGTTTTAACGTCRTCCCCDCCTTCCTC | Fbc11 | AATGATACGGCGACCACCGAGATCTACACAATTGTGTCGGATACCGGGACTTAGGATTAGATACCCBRGTAGTC |
| 326 | Rrcbc45 | CAAGCAGAAGACGGCATACGAGATTCGGAATTAGACAACACGTTTTAACGTCRTCCCCDCCTTCCTC | Fbc11 | AATGATACGGCGACCACCGAGATCTACACAATTGTGTCGGATACCGGGACTTAGGATTAGATACCCBRGTAGTC |
| 327 | Rrcbc46 | CAAGCAGAAGACGGCATACGAGATTGTGAATTCGGAAACACGTTTTAACGTCRTCCCCDCCTTCCTC | Fbc11 | AATGATACGGCGACCACCGAGATCTACACAATTGTGTCGGATACCGGGACTTAGGATTAGATACCCBRGTAGTC |
| 328 | Rrcbc47 | CAAGCAGAAGACGGCATACGAGATCATTCGTGGCGTAACACGTTTTAACGTCRTCCCCDCCTTCCTC | Fbc11 | AATGATACGGCGACCACCGAGATCTACACAATTGTGTCGGATACCGGGACTTAGGATTAGATACCCBRGTAGTC |
| 329 | Rrcbc48 | CAAGCAGAAGACGGCATACGAGATTACTACGTGGCCAACACGTTTTAACGTCRTCCCCDCCTTCCTC | Fbc11 | AATGATACGGCGACCACCGAGATCTACACAATTGTGTCGGATACCGGGACTTAGGATTAGATACCCBRGTAGTC |
| 330 | Rrcbc49 | CAAGCAGAAGACGGCATACGAGATGGCCAGTTCCTAAACACGTTTTAACGTCRTCCCCDCCTTCCTC | Fbc11 | AATGATACGGCGACCACCGAGATCTACACAATTGTGTCGGATACCGGGACTTAGGATTAGATACCCBRGTAGTC |
| 331 | Rrcbc50 | CAAGCAGAAGACGGCATACGAGATGATGTTCGCTAGAACACGTTTTAACGTCRTCCCCDCCTTCCTC | Fbc11 | AATGATACGGCGACCACCGAGATCTACACAATTGTGTCGGATACCGGGACTTAGGATTAGATACCCBRGTAGTC |
| 332 | Rrcbc51 | CAAGCAGAAGACGGCATACGAGATCTATCTCCTGTCAACACGTTTTAACGTCRTCCCCDCCTTCCTC | Fbc11 | AATGATACGGCGACCACCGAGATCTACACAATTGTGTCGGATACCGGGACTTAGGATTAGATACCCBRGTAGTC |
| 333 | Rrcbc52 | CAAGCAGAAGACGGCATACGAGATACTCACAGGAATAACACGTTTTAACGTCRTCCCCDCCTTCCTC | Fbc11 | AATGATACGGCGACCACCGAGATCTACACAATTGTGTCGGATACCGGGACTTAGGATTAGATACCCBRGTAGTC |
| 334 | Rrcbc53 | CAAGCAGAAGACGGCATACGAGATATGATGAGCCTCAACACGTTTTAACGTCRTCCCCDCCTTCCTC | Fbc11 | AATGATACGGCGACCACCGAGATCTACACAATTGTGTCGGATACCGGGACTTAGGATTAGATACCCBRGTAGTC |
| 335 | Rrcbc54 | CAAGCAGAAGACGGCATACGAGATGTCGACAGAGGAAACACGTTTTAACGTCRTCCCCDCCTTCCTC | Fbc11 | AATGATACGGCGACCACCGAGATCTACACAATTGTGTCGGATACCGGGACTTAGGATTAGATACCCBRGTAGTC |
| 336 | Rrcbc55 | CAAGCAGAAGACGGCATACGAGATTGTCGCAAATAGAACACGTTTTAACGTCRTCCCCDCCTTCCTC | Fbc11 | AATGATACGGCGACCACCGAGATCTACACAATTGTGTCGGATACCGGGACTTAGGATTAGATACCCBRGTAGTC |
| 337 | Rrcbc44 | CAAGCAGAAGACGGCATACGAGATTAATACGGATCGAACACGTTTTAACGTCRTCCCCDCCTTCCTC | Fbc12 | AATGATACGGCGACCACCGAGATCTACACTGCATACACTGGTACCGGGACTTAGGATTAGATACCCBRGTAGTC |
| 338 | Rrcbc45 | CAAGCAGAAGACGGCATACGAGATTCGGAATTAGACAACACGTTTTAACGTCRTCCCCDCCTTCCTC | Fbc12 | AATGATACGGCGACCACCGAGATCTACACTGCATACACTGGTACCGGGACTTAGGATTAGATACCCBRGTAGTC |
| 339 | Rrcbc46 | CAAGCAGAAGACGGCATACGAGATTGTGAATTCGGAAACACGTTTTAACGTCRTCCCCDCCTTCCTC | Fbc12 | AATGATACGGCGACCACCGAGATCTACACTGCATACACTGGTACCGGGACTTAGGATTAGATACCCBRGTAGTC |
| 340 | Rrcbc47 | CAAGCAGAAGACGGCATACGAGATCATTCGTGGCGTAACACGTTTTAACGTCRTCCCCDCCTTCCTC | Fbc12 | AATGATACGGCGACCACCGAGATCTACACTGCATACACTGGTACCGGGACTTAGGATTAGATACCCBRGTAGTC |
| 341 | Rrcbc48 | CAAGCAGAAGACGGCATACGAGATTACTACGTGGCCAACACGTTTTAACGTCRTCCCCDCCTTCCTC | Fbc12 | AATGATACGGCGACCACCGAGATCTACACTGCATACACTGGTACCGGGACTTAGGATTAGATACCCBRGTAGTC |
| 342 | Rrcbc49 | CAAGCAGAAGACGGCATACGAGATGGCCAGTTCCTAAACACGTTTTAACGTCRTCCCCDCCTTCCTC | Fbc12 | AATGATACGGCGACCACCGAGATCTACACTGCATACACTGGTACCGGGACTTAGGATTAGATACCCBRGTAGTC |
| 343 | Rrcbc50 | CAAGCAGAAGACGGCATACGAGATGATGTTCGCTAGAACACGTTTTAACGTCRTCCCCDCCTTCCTC | Fbc12 | AATGATACGGCGACCACCGAGATCTACACTGCATACACTGGTACCGGGACTTAGGATTAGATACCCBRGTAGTC |
| 344 | Rrcbc51 | CAAGCAGAAGACGGCATACGAGATCTATCTCCTGTCAACACGTTTTAACGTCRTCCCCDCCTTCCTC | Fbc12 | AATGATACGGCGACCACCGAGATCTACACTGCATACACTGGTACCGGGACTTAGGATTAGATACCCBRGTAGTC |
| 345 | Rrcbc52 | CAAGCAGAAGACGGCATACGAGATACTCACAGGAATAACACGTTTTAACGTCRTCCCCDCCTTCCTC | Fbc12 | AATGATACGGCGACCACCGAGATCTACACTGCATACACTGGTACCGGGACTTAGGATTAGATACCCBRGTAGTC |
| 346 | Rrcbc53 | CAAGCAGAAGACGGCATACGAGATATGATGAGCCTCAACACGTTTTAACGTCRTCCCCDCCTTCCTC | Fbc12 | AATGATACGGCGACCACCGAGATCTACACTGCATACACTGGTACCGGGACTTAGGATTAGATACCCBRGTAGTC |
| 347 | Rrcbc54 | CAAGCAGAAGACGGCATACGAGATGTCGACAGAGGAAACACGTTTTAACGTCRTCCCCDCCTTCCTC | Fbc12 | AATGATACGGCGACCACCGAGATCTACACTGCATACACTGGTACCGGGACTTAGGATTAGATACCCBRGTAGTC |
| 348 | Rrcbc55 | CAAGCAGAAGACGGCATACGAGATTGTCGCAAATAGAACACGTTTTAACGTCRTCCCCDCCTTCCTC | Fbc12 | AATGATACGGCGACCACCGAGATCTACACTGCATACACTGGTACCGGGACTTAGGATTAGATACCCBRGTAGTC |
| 349 | Rrcbc44 | CAAGCAGAAGACGGCATACGAGATTAATACGGATCGAACACGTTTTAACGTCRTCCCCDCCTTCCTC | Fbc13 | AATGATACGGCGACCACCGAGATCTACACAGTCGAACGAGGTACCGGGACTTAGGATTAGATACCCBRGTAGTC |
| 350 | Rrcbc45 | CAAGCAGAAGACGGCATACGAGATTCGGAATTAGACAACACGTTTTAACGTCRTCCCCDCCTTCCTC | Fbc13 | AATGATACGGCGACCACCGAGATCTACACAGTCGAACGAGGTACCGGGACTTAGGATTAGATACCCBRGTAGTC |
| 351 | Rrcbc46 | CAAGCAGAAGACGGCATACGAGATTGTGAATTCGGAAACACGTTTTAACGTCRTCCCCDCCTTCCTC | Fbc13 | AATGATACGGCGACCACCGAGATCTACACAGTCGAACGAGGTACCGGGACTTAGGATTAGATACCCBRGTAGTC |
| 352 | Rrcbc47 | CAAGCAGAAGACGGCATACGAGATCATTCGTGGCGTAACACGTTTTAACGTCRTCCCCDCCTTCCTC | Fbc13 | AATGATACGGCGACCACCGAGATCTACACAGTCGAACGAGGTACCGGGACTTAGGATTAGATACCCBRGTAGTC |
| 353 | Rrcbc48 | CAAGCAGAAGACGGCATACGAGATTACTACGTGGCCAACACGTTTTAACGTCRTCCCCDCCTTCCTC | Fbc13 | AATGATACGGCGACCACCGAGATCTACACAGTCGAACGAGGTACCGGGACTTAGGATTAGATACCCBRGTAGTC |
| 354 | Rrcbc49 | CAAGCAGAAGACGGCATACGAGATGGCCAGTTCCTAAACACGTTTTAACGTCRTCCCCDCCTTCCTC | Fbc13 | AATGATACGGCGACCACCGAGATCTACACAGTCGAACGAGGTACCGGGACTTAGGATTAGATACCCBRGTAGTC |
| 355 | Rrcbc50 | CAAGCAGAAGACGGCATACGAGATGATGTTCGCTAGAACACGTTTTAACGTCRTCCCCDCCTTCCTC | Fbc13 | AATGATACGGCGACCACCGAGATCTACACAGTCGAACGAGGTACCGGGACTTAGGATTAGATACCCBRGTAGTC |
| 356 | Rrcbc51 | CAAGCAGAAGACGGCATACGAGATCTATCTCCTGTCAACACGTTTTAACGTCRTCCCCDCCTTCCTC | Fbc13 | AATGATACGGCGACCACCGAGATCTACACAGTCGAACGAGGTACCGGGACTTAGGATTAGATACCCBRGTAGTC |
| 357 | Rrcbc52 | CAAGCAGAAGACGGCATACGAGATACTCACAGGAATAACACGTTTTAACGTCRTCCCCDCCTTCCTC | Fbc13 | AATGATACGGCGACCACCGAGATCTACACAGTCGAACGAGGTACCGGGACTTAGGATTAGATACCCBRGTAGTC |
| 358 | Rrcbc53 | CAAGCAGAAGACGGCATACGAGATATGATGAGCCTCAACACGTTTTAACGTCRTCCCCDCCTTCCTC | Fbc13 | AATGATACGGCGACCACCGAGATCTACACAGTCGAACGAGGTACCGGGACTTAGGATTAGATACCCBRGTAGTC |
| 359 | Rrcbc54 | CAAGCAGAAGACGGCATACGAGATGTCGACAGAGGAAACACGTTTTAACGTCRTCCCCDCCTTCCTC | Fbc13 | AATGATACGGCGACCACCGAGATCTACACAGTCGAACGAGGTACCGGGACTTAGGATTAGATACCCBRGTAGTC |
| 360 | Rrcbc55 | CAAGCAGAAGACGGCATACGAGATTGTCGCAAATAGAACACGTTTTAACGTCRTCCCCDCCTTCCTC | Fbc13 | AATGATACGGCGACCACCGAGATCTACACAGTCGAACGAGGTACCGGGACTTAGGATTAGATACCCBRGTAGTC |
| 361 | Rrcbc44 | CAAGCAGAAGACGGCATACGAGATTAATACGGATCGAACACGTTTTAACGTCRTCCCCDCCTTCCTC | Fbc14 | AATGATACGGCGACCACCGAGATCTACACACCAGTGACTCATACCGGGACTTAGGATTAGATACCCBRGTAGTC |
| 362 | Rrcbc45 | CAAGCAGAAGACGGCATACGAGATTCGGAATTAGACAACACGTTTTAACGTCRTCCCCDCCTTCCTC | Fbc14 | AATGATACGGCGACCACCGAGATCTACACACCAGTGACTCATACCGGGACTTAGGATTAGATACCCBRGTAGTC |
| 363 | Rrcbc46 | CAAGCAGAAGACGGCATACGAGATTGTGAATTCGGAAACACGTTTTAACGTCRTCCCCDCCTTCCTC | Fbc14 | AATGATACGGCGACCACCGAGATCTACACACCAGTGACTCATACCGGGACTTAGGATTAGATACCCBRGTAGTC |
| 364 | Rrcbc47 | CAAGCAGAAGACGGCATACGAGATCATTCGTGGCGTAACACGTTTTAACGTCRTCCCCDCCTTCCTC | Fbc14 | AATGATACGGCGACCACCGAGATCTACACACCAGTGACTCATACCGGGACTTAGGATTAGATACCCBRGTAGTC |
| 365 | Rrcbc48 | CAAGCAGAAGACGGCATACGAGATTACTACGTGGCCAACACGTTTTAACGTCRTCCCCDCCTTCCTC | Fbc14 | AATGATACGGCGACCACCGAGATCTACACACCAGTGACTCATACCGGGACTTAGGATTAGATACCCBRGTAGTC |
| 366 | Rrcbc49 | CAAGCAGAAGACGGCATACGAGATGGCCAGTTCCTAAACACGTTTTAACGTCRTCCCCDCCTTCCTC | Fbc14 | AATGATACGGCGACCACCGAGATCTACACACCAGTGACTCATACCGGGACTTAGGATTAGATACCCBRGTAGTC |
| 367 | Rrcbc50 | CAAGCAGAAGACGGCATACGAGATGATGTTCGCTAGAACACGTTTTAACGTCRTCCCCDCCTTCCTC | Fbc14 | AATGATACGGCGACCACCGAGATCTACACACCAGTGACTCATACCGGGACTTAGGATTAGATACCCBRGTAGTC |
| 368 | Rrcbc51 | CAAGCAGAAGACGGCATACGAGATCTATCTCCTGTCAACACGTTTTAACGTCRTCCCCDCCTTCCTC | Fbc14 | AATGATACGGCGACCACCGAGATCTACACACCAGTGACTCATACCGGGACTTAGGATTAGATACCCBRGTAGTC |
| 369 | Rrcbc52 | CAAGCAGAAGACGGCATACGAGATACTCACAGGAATAACACGTTTTAACGTCRTCCCCDCCTTCCTC | Fbc14 | AATGATACGGCGACCACCGAGATCTACACACCAGTGACTCATACCGGGACTTAGGATTAGATACCCBRGTAGTC |
| 370 | Rrcbc53 | CAAGCAGAAGACGGCATACGAGATATGATGAGCCTCAACACGTTTTAACGTCRTCCCCDCCTTCCTC | Fbc14 | AATGATACGGCGACCACCGAGATCTACACACCAGTGACTCATACCGGGACTTAGGATTAGATACCCBRGTAGTC |
| 371 | Rrcbc54 | CAAGCAGAAGACGGCATACGAGATGTCGACAGAGGAAACACGTTTTAACGTCRTCCCCDCCTTCCTC | Fbc14 | AATGATACGGCGACCACCGAGATCTACACACCAGTGACTCATACCGGGACTTAGGATTAGATACCCBRGTAGTC |
| 372 | Rrcbc55 | CAAGCAGAAGACGGCATACGAGATTGTCGCAAATAGAACACGTTTTAACGTCRTCCCCDCCTTCCTC | Fbc14 | AATGATACGGCGACCACCGAGATCTACACACCAGTGACTCATACCGGGACTTAGGATTAGATACCCBRGTAGTC |
| 373 | Rrcbc44 | CAAGCAGAAGACGGCATACGAGATTAATACGGATCGAACACGTTTTAACGTCRTCCCCDCCTTCCTC | Fbc15 | AATGATACGGCGACCACCGAGATCTACACGAATACCAAGTCTACCGGGACTTAGGATTAGATACCCBRGTAGTC |
| 374 | Rrcbc45 | CAAGCAGAAGACGGCATACGAGATTCGGAATTAGACAACACGTTTTAACGTCRTCCCCDCCTTCCTC | Fbc15 | AATGATACGGCGACCACCGAGATCTACACGAATACCAAGTCTACCGGGACTTAGGATTAGATACCCBRGTAGTC |
| 375 | Rrcbc46 | CAAGCAGAAGACGGCATACGAGATTGTGAATTCGGAAACACGTTTTAACGTCRTCCCCDCCTTCCTC | Fbc15 | AATGATACGGCGACCACCGAGATCTACACGAATACCAAGTCTACCGGGACTTAGGATTAGATACCCBRGTAGTC |
| 376 | Rrcbc47 | CAAGCAGAAGACGGCATACGAGATCATTCGTGGCGTAACACGTTTTAACGTCRTCCCCDCCTTCCTC | Fbc15 | AATGATACGGCGACCACCGAGATCTACACGAATACCAAGTCTACCGGGACTTAGGATTAGATACCCBRGTAGTC |
| 377 | Rrcbc48 | CAAGCAGAAGACGGCATACGAGATTACTACGTGGCCAACACGTTTTAACGTCRTCCCCDCCTTCCTC | Fbc15 | AATGATACGGCGACCACCGAGATCTACACGAATACCAAGTCTACCGGGACTTAGGATTAGATACCCBRGTAGTC |
| 378 | Rrcbc49 | CAAGCAGAAGACGGCATACGAGATGGCCAGTTCCTAAACACGTTTTAACGTCRTCCCCDCCTTCCTC | Fbc15 | AATGATACGGCGACCACCGAGATCTACACGAATACCAAGTCTACCGGGACTTAGGATTAGATACCCBRGTAGTC |
| 379 | Rrcbc50 | CAAGCAGAAGACGGCATACGAGATGATGTTCGCTAGAACACGTTTTAACGTCRTCCCCDCCTTCCTC | Fbc15 | AATGATACGGCGACCACCGAGATCTACACGAATACCAAGTCTACCGGGACTTAGGATTAGATACCCBRGTAGTC |
| 380 | Rrcbc51 | CAAGCAGAAGACGGCATACGAGATCTATCTCCTGTCAACACGTTTTAACGTCRTCCCCDCCTTCCTC | Fbc15 | AATGATACGGCGACCACCGAGATCTACACGAATACCAAGTCTACCGGGACTTAGGATTAGATACCCBRGTAGTC |
| 381 | Rrcbc52 | CAAGCAGAAGACGGCATACGAGATACTCACAGGAATAACACGTTTTAACGTCRTCCCCDCCTTCCTC | Fbc15 | AATGATACGGCGACCACCGAGATCTACACGAATACCAAGTCTACCGGGACTTAGGATTAGATACCCBRGTAGTC |
| 382 | Rrcbc53 | CAAGCAGAAGACGGCATACGAGATATGATGAGCCTCAACACGTTTTAACGTCRTCCCCDCCTTCCTC | Fbc15 | AATGATACGGCGACCACCGAGATCTACACGAATACCAAGTCTACCGGGACTTAGGATTAGATACCCBRGTAGTC |
| 383 | Rrcbc54 | CAAGCAGAAGACGGCATACGAGATGTCGACAGAGGAAACACGTTTTAACGTCRTCCCCDCCTTCCTC | Fbc15 | AATGATACGGCGACCACCGAGATCTACACGAATACCAAGTCTACCGGGACTTAGGATTAGATACCCBRGTAGTC |
| 384 | Rrcbc55 | CAAGCAGAAGACGGCATACGAGATTGTCGCAAATAGAACACGTTTTAACGTCRTCCCCDCCTTCCTC | Fbc15 | AATGATACGGCGACCACCGAGATCTACACGAATACCAAGTCTACCGGGACTTAGGATTAGATACCCBRGTAGTC |
