# Supplementary material for: Bacterial communities found in placental tissues are associated with severe chorioamnionitis and adverse birth outcomes
Source: PLoS One. 2017 Jul 12;12(7):e0180167. doi: 10.1371/journal.pone.0180167 (PMC5507499; doi:10.1371/journal.pone.0180167)
Supplement: S2 Table — (DOCX) [file pone.0180167.s005.docx]

**Table S2. Number of sequenced reads generated from placental and fetal membrane samples.**

| **Sample type** | **Number of samples sequenced** | **Median number of reads per sample (IQR)** | **Total number of reads** |
| --- | --- | --- | --- |
| Placenta | 476 | 11,803 (3800,33561) | 14,001,032 |
| Fetal membrane | 738 | 21,040 (7340,54473) | 30,941,823 |
| Total | 1214 | - | 44,942,855 |
