# Supplementary material for: Bacterial communities found in placental tissues are associated with severe chorioamnionitis and adverse birth outcomes
Source: PLoS One. 2017 Jul 12;12(7):e0180167. doi: 10.1371/journal.pone.0180167 (PMC5507499; doi:10.1371/journal.pone.0180167)
Supplement: S3 Table — (DOCX) [file pone.0180167.s006.docx]

Table S3. The association between OTU relative abundance and the time between delivery and processing of placental tissue.

| O.T.U ID (Custom database and greengenes) | Spearman’s rho | *P* value^1^ | *q* value |
| --- | --- | --- | --- |
| 9041 | 0.140682 | 0.003914 | 0.238725 |
| 10268 | -0.12648 | 0.009586 | 0.292375 |
| 11344 | 0.115514 | 0.018091 | 0.321275 |
| 336454741 | 0.109867 | 0.024625 | 0.321275 |
| 3431 | 0.108607 | 0.026334 | 0.321275 |
| 1412 | -0.10012 | 0.040716 | 0.401834 |
| 5377 | -0.09606 | 0.04966 | 0.401834 |
| 659364527 | 0.09418 | 0.054318 | 0.401834 |
| 5697 | -0.09232 | 0.059287 | 0.401834 |
| CP003293.1 | -0.08142 | 0.096431 | 0.588232 |
| 3677 | 0.075163 | 0.12501 | 0.679969 |
| 588052 | 0.073471 | 0.133764 | 0.679969 |
| 1427 | -0.06648 | 0.17503 | 0.686661 |
| 11485 | 0.063758 | 0.193388 | 0.686661 |
| 12017 | 0.063725 | 0.193621 | 0.686661 |
| 2168 | 0.063093 | 0.198089 | 0.686661 |
| 2287 | -0.06116 | 0.212239 | 0.686661 |
| 6038 | -0.05932 | 0.226315 | 0.686661 |
| 10708 | -0.05885 | 0.230058 | 0.686661 |
| 323575284 | 0.058752 | 0.230817 | 0.686661 |
| 11025 | -0.05806 | 0.236391 | 0.686661 |
| 10002 | 0.055516 | 0.257591 | 0.687616 |
| 517331 | -0.05377 | 0.272863 | 0.687616 |
| 558508579 | 0.052984 | 0.279976 | 0.687616 |
| 10729 | 0.050061 | 0.307413 | 0.687616 |
| 7998 | -0.04955 | 0.312421 | 0.687616 |
| 206975 | 0.049462 | 0.313242 | 0.687616 |
| 296597 | -0.04756 | 0.332255 | 0.687616 |
| 444439626 | -0.04697 | 0.338236 | 0.687616 |
| 42521630 | -0.0453 | 0.355789 | 0.687616 |
| 10374 | -0.04483 | 0.360804 | 0.687616 |
| 10703 | 0.043724 | 0.372766 | 0.687616 |
| 115278 | 0.043499 | 0.375238 | 0.687616 |
| 1340 | -0.04193 | 0.392746 | 0.687616 |
| 6074 | -0.04177 | 0.394534 | 0.687616 |
| 9910 | 0.036835 | 0.452813 | 0.75698 |
| 5119 | 0.036321 | 0.459152 | 0.75698 |
| 7525 | 0.032987 | 0.501425 | 0.790452 |
| 7213 | 0.032684 | 0.505371 | 0.790452 |
| 512134444 | 0.026925 | 0.583252 | 0.88946 |
| 5749 | 0.02466 | 0.615345 | 0.915513 |
| CP003604.1 | -0.02252 | 0.646341 | 0.917925 |
| 8654 | -0.02247 | 0.647062 | 0.917925 |
| 5587 | 0.01953 | 0.6907 | 0.949577 |
| 101445 | 0.015987 | 0.744646 | 0.949577 |
| 5756 | -0.01521 | 0.756701 | 0.949577 |
| 5631 | -0.01515 | 0.757636 | 0.949577 |
| 12130 | 0.014911 | 0.761292 | 0.949577 |
| 10346 | 0.014816 | 0.762775 | 0.949577 |
| CP006768.1 | 0.012989 | 0.791296 | 0.965381 |
| 7307 | 0.010763 | 0.82645 | 0.988499 |
| 3851 | -0.00923 | 0.850921 | 0.98914 |
| 4717 | 0.007728 | 0.8749 | 0.98914 |
| 5266 | -0.00585 | 0.905199 | 0.98914 |
| 7073 | 0.005639 | 0.908551 | 0.98914 |
| 10299 | -0.00499 | 0.918997 | 0.98914 |
| 6955 | -0.0046 | 0.92529 | 0.98914 |
| 7906 | -0.00318 | 0.948391 | 0.98914 |
| 4280 | 0.002344 | 0.961907 | 0.98914 |
| 645321357 | -0.00167 | 0.972925 | 0.98914 |
| 1467 | 0.000351 | 0.994294 | 0.994294 |

^1^ P value calculated using Spearmans’s correlation.
