# Supplementary material for: Bacterial communities found in placental tissues are associated with severe chorioamnionitis and adverse birth outcomes
Source: PLoS One. 2017 Jul 12;12(7):e0180167. doi: 10.1371/journal.pone.0180167 (PMC5507499; doi:10.1371/journal.pone.0180167)
Supplement: S4 Table — (DOCX) [file pone.0180167.s007.docx]

Table S4. The association between OTU relative abundance and the time between delivery and processing of fetal membrane tissue.

| O.T.U ID (Custom database and greengenes) | Spearman’s rho | *P* value^1^ | *q* value |
| --- | --- | --- | --- |
| 9041 | 0.129586 | 0.00073 | 0.047533 |
| 3431 | 0.122792 | 0.001378 | 0.047533 |
| 659364527 | 0.110426 | 0.004049 | 0.070008 |
| 3677 | 0.110399 | 0.004058 | 0.070008 |
| 323575284 | 0.10226 | 0.00781 | 0.107777 |
| 336454741 | 0.090605 | 0.018513 | 0.212896 |
| 12130 | 0.085991 | 0.025439 | 0.250758 |
| 4974 | -0.08019 | 0.037223 | 0.321051 |
| 772 | 0.076012 | 0.048352 | 0.370702 |
| 4717 | 0.065347 | 0.089807 | 0.588802 |
| 4280 | -0.06454 | 0.093867 | 0.588802 |
| 133171 | -0.05682 | 0.14035 | 0.806246 |
| 134528 | -0.05299 | 0.169146 | 0.806246 |
| 10002 | 0.052645 | 0.171945 | 0.806246 |
| 1475 | -0.05169 | 0.179885 | 0.806246 |
| 11025 | -0.05043 | 0.190757 | 0.806246 |
| 10703 | -0.04912 | 0.202528 | 0.806246 |
| 7446 | 0.048285 | 0.210325 | 0.806246 |
| 3316 | -0.04421 | 0.251495 | 0.913323 |
| 288932 | -0.04106 | 0.286879 | 0.93976 |
| 10374 | -0.0366 | 0.342496 | 0.93976 |
| 4058 | 0.036562 | 0.343018 | 0.93976 |
| 206975 | -0.03432 | 0.373425 | 0.93976 |
| 6074 | 0.034214 | 0.374929 | 0.93976 |
| 10091 | 0.033965 | 0.378422 | 0.93976 |
| 12502 | -0.03339 | 0.386591 | 0.93976 |
| 6038 | -0.03312 | 0.390383 | 0.93976 |
| 2287 | -0.03304 | 0.391532 | 0.93976 |
| 2167 | 0.032802 | 0.394972 | 0.93976 |
| 444439626 | -0.03127 | 0.417444 | 0.942912 |
| CP003293.1 | -0.03032 | 0.431752 | 0.942912 |
| 1412 | -0.02886 | 0.454306 | 0.942912 |
| 588052 | -0.0275 | 0.475835 | 0.942912 |
| 5266 | -0.02656 | 0.491033 | 0.942912 |
| 5756 | 0.025801 | 0.503499 | 0.942912 |
| 10268 | -0.02535 | 0.510962 | 0.942912 |
| 11344 | 0.02519 | 0.513667 | 0.942912 |
| 7073 | -0.02486 | 0.519285 | 0.942912 |
| 296597 | -0.0232 | 0.547484 | 0.952637 |
| 558508579 | -0.02206 | 0.56736 | 0.952637 |
| 7906 | 0.021976 | 0.568827 | 0.952637 |
| 10346 | -0.0209 | 0.587904 | 0.952637 |
| 6955 | 0.020578 | 0.593672 | 0.952637 |
| 5631 | 0.018293 | 0.635307 | 0.996278 |
| 3851 | 0.015611 | 0.685686 | 0.997852 |
| 12017 | 0.014779 | 0.701609 | 0.997852 |
| 5377 | 0.013961 | 0.7174 | 0.997852 |
| 7307 | -0.01335 | 0.729315 | 0.997852 |
| 10729 | -0.01167 | 0.762317 | 0.997852 |
| 1340 | -0.01105 | 0.774624 | 0.997852 |
| 1427 | 0.009002 | 0.815485 | 0.997852 |
| 517331 | 0.006699 | 0.862129 | 0.997852 |
| 1283 | 0.006203 | 0.872254 | 0.997852 |
| 10708 | -0.00558 | 0.884983 | 0.997852 |
| CP003604.1 | 0.005243 | 0.891883 | 0.997852 |
| 5749 | -0.00436 | 0.910052 | 0.997852 |
| 5697 | 0.004288 | 0.911497 | 0.997852 |
| 10299 | 0.003715 | 0.923274 | 0.997852 |
| 137258 | -0.00352 | 0.927324 | 0.997852 |
| 1467 | 0.003102 | 0.935918 | 0.997852 |
| 42521630 | -0.0024 | 0.950342 | 0.997852 |
| 7998 | -0.00205 | 0.957603 | 0.997852 |
| 338950 | -0.00187 | 0.961287 | 0.997852 |
| 7213 | -0.0009 | 0.981415 | 0.997852 |
| 12049 | -0.00083 | 0.982866 | 0.997852 |
| 631251895 | -0.00079 | 0.983617 | 0.997852 |
| CP006768.1 | 0.000732 | 0.984864 | 0.997852 |
| 12240234 | 0.000382 | 0.992094 | 0.997852 |
| 645321357 | -0.0001 | 0.997852 | 0.997852 |

^1^ P value calculated using Spearmans’s correlation.
