# Supplementary material for: Bacterial communities found in placental tissues are associated with severe chorioamnionitis and adverse birth outcomes
Source: PLoS One. 2017 Jul 12;12(7):e0180167. doi: 10.1371/journal.pone.0180167 (PMC5507499; doi:10.1371/journal.pone.0180167)
Supplement: S6 Table — (DOCX) [file pone.0180167.s009.docx]

Table S4. OTUs isolated from fetal membranes significantly associated with differences in birth weight.

|  |  |  | Unadjusted analysis | | | Adjusted analysis | | |
| --- | --- | --- | --- | --- | --- | --- | --- | --- |
| O.T.U ID (Custom database and greengenes) | Greengenes Taxonomy | Genbank BLASTN result | Correlation coefficient | *P* value^1^ | N | Regression coefficient (95%CI) | *P* value^2^ | *q* value |
| 645321357 | g__Sneathia; s__ | *Sneathia sanguinegens* | -0.08 | 0.016 | 1005 | -22 (-40, -5) | 0.011 | 0.341 |
| 288932 | g__Prevotella; s__copri | *Prevotella copri* | -0.08 | 0.014 | 1005 | -48 (-90, -8) | 0.020 | 0.310 |
| 631251895 | g__Prevotella; s__ | *Prevotella amnii* | -0.07 | 0.037 | 1005 | -22 (-54, 9) | 0.158 | 1.000 |

^1^ P value calculated using Pearson’s correlation.

^2^ Adjusted p-values were calculated using linear regression models. Regression coefficient shows the change in head circumference-for-age Z-score in relation to an increase in bacterial load of one Log10 genome/µl. All models were adjusted for the intervention, maternal BMI at enrolment, maternal age, proxy for socioeconomic status, number of previous pregnancies, anaemia, site of enrolment, mode of delivery and time between delivery and placenta sampling.
